# The efficacy and safety of Chinese herbal medicine Guizhi Fuling capsule combined with low dose mifepristone in the treatment of uterine fibroids: a systematic review and meta-analysis of 28 randomized controlled trials

**DOI:** 10.1186/s12906-023-03842-y

**Published:** 2023-02-18

**Authors:** Yiming Lei, Lili Yang, Honglian Yang, Min Li, Li Ou, Yang Bai, Taiwei Dong, Feng Gao, Peifeng Wei

**Affiliations:** 1 Shaanxi University of Chinese Medicine, Xianyang, China; 2 The Second Affiliated Hospital of Shaanxi University of Chinese Medicine, Xianyang, China

**Keywords:** Guizhi Fuling capsule, Uterine fibroids, Mifepristone, Systematic review, Meta-analysis

## Abstract

**Objective:**

Guizhi Fuling Capsule (GZFL), a classic traditional Chinese medicine prescription, is often recommended for the treatment of uterine fibroids (UFs). However, the efficacy and safety of GZFL in combination with low-dose mifepristone (MFP) remains controversial.

**Materials and methods:**

We searched eight literature databases and two clinical trial registries for randomized controlled trials (RCTs) of the efficacy and safety of GZFL combined with low-dose MFP in the treatment of UFs from database inception to April 24, 2022. Data analysis was performed using the Meta package in RStudio and RevMan 5.4. GRADE pro3.6.1 software was used for the assessment of evidence quality.

**Results:**

Twenty-eight RCTs were included in this study, including a total of 2813 patients. The meta-analysis showed that compared with low-dose MFP alone, GZFL combined with low-dose MFP significantly reduced follicle stimulating hormone (*p* < 0.001), estradiol (*p* < 0.001), progesterone (*p* < 0.001), luteinizing hormone (*p* < 0.001), uterine fibroids volume (*p* < 0.001), uterine volume (*p* < 0.001), menstrual flow (*p* < 0.001) and increased clinical efficiency rate (*p* < 0.001). Meanwhile, GZFL combined with low-dose MFP did not significantly increase the incidence of adverse drug reactions compared with low-dose MFP alone (*p* = 0.16). The quality of the evidence for the outcomes ranged from “very low” to “moderate.”

**Conclusion:**

This study suggests that GZFL combined with low-dose MFP is more effective and safe in the treatment of UFs, and it is a potential treatment for UFs. However, due to the poor quality of the included RCTs formulations, we recommend a rigorous, high-quality, large-sample trial to confirm our findings.

**Supplementary Information:**

The online version contains supplementary material available at 10.1186/s12906-023-03842-y.

## Introduction

Uterine fibroids (UFs, also known as leiomyomas or fibroids) are monoclonal tumors that originate in the smooth muscle cells of the myometrium and are most common in women of reproductive age, with a worldwide prevalence of over 75%, is a benign uterine tumor [1–4]. Among women of reproductive age, the incidence of UFs is 50 to 60%, rising to 70% by age 50 [5, 6]. However, many UFs may be asymptomatic [7, 8], but in 25 to 50% of cases, they display a variety of symptoms depending on location and size [6, 9, 10]. Evidence suggests that leiomyomas that are closer to the endometrial cavity (submucosal) are more likely to cause heavy menstrual bleeding, which can lead to anemia; dysmenorrhea and pelvic pain also occur frequently, affecting the quality of life and daily activities. Infertility and recurrent miscarriages may also occur when UFs are distributed in submucosal and intramural fibroids that twist the uterine cavity. Larger diameter fibroids may lead to bowel and bladder dysfunction, including frequent urination, constipation, or distorted abdominal wall contours [2, 6, 11–15].

Hysterectomy remains the primary treatment to date [12, 16]. However, a growing body of research has found that even with the preservation of both ovaries, a hysterectomy carries considerable health risks, including increased cardiovascular risk, mood disturbance, and urinary dysfunction [2, 17]. Gonadotropin-releasing hormone analog (GnRHa) can reduce tumor size and improve other symptoms, but decreased bone mineral density and the development of vasomotor symptoms may be side effects of this treatment [5, 18, 19]. Clinically, selective progesterone receptor modulators (SPRMs) are attractive because of their reduced side effects on non-target tissues such as the breast and brain. Depending on the cell type and molecular context, SPRMs can act as agonists or antagonists of progesterone receptors (PR) [5]. By combining with PR, SPRMs block the stimulating effect of progesterone, thereby achieving the effect of promoting fibroid shrinkage and reducing fibroids volume [20], and are widely used in the treatment of UFs. Mifepristone, an anabolic steroid with antiprogesterone and antiglucocorticoid activity, is a commonly used SPRM. MFP is effective and well-tolerated in the treatment of UFs [19, 21, 22]. Multiple past studies have demonstrated that low-dose MFP maintains the same efficacy and has fewer adverse effects than high-dose MFP [20, 23–25]. Although various treatment regimens for UFs have been investigated, unfortunately, none of these regimens have proven to be a perfect solution for leiomyoma management in the majority of patients with UFs, and remission of UFs remains suboptimal [26].

Therefore, the need to seek new medical treatments remains a reality, and a safer, cost-effective approach is highly warranted. So new treatment options or drugs must be found so that treatment can be provided. In recent years, Chinese herbal medicine, as a representative of complementary and alternative medicine, has attracted extensive attention in the treatment of UFs [27, 28]. GZFL is a Chinese herbal formula widely used in gynecological diseases in China. GZFL is exclusively produced by Jiangsu Kangyuan Meiyu Biopharmaceutical Co., Ltd. It consists of five herbs *Cinnamomum cassia* (L.) J.Presl, Lauraceae; *Paeonia* × *suffruticosa* Andrews, Paeoniaceae; *Poria cocos*(Schw.)Wolf, Polyporaceae; *Paeonia lactiflora* Pall., Paeoniaceae; and *Prunus persica* (L.) Batsch or *Prunus davidiana* (CarriŠre) Franch., Rosaceae. The details are provided in Table 1. Modern pharmacological experiments show that GZFL can inhibit the proliferation, migration, and invasion of UFs, and increase the levels of CD3^+^, CD4^+^/CD8^+^. In addition, GZFL can induce the apoptosis of UFs and inhibit cell proliferation by down-regulating the expression of Wnt/β-Catenin signaling pathway-related proteins and mRNAs [29, 30]. A randomized controlled trial showed that after a one-year follow-up of GZFL treatment, there was no recurrence in the GZFL group, and the incidence of adverse reactions was significantly lower than that in the control group. The GZFL group showed good efficacy and safety [31]. In addition, the application guidelines of GZFL have been published in China, and GZFL is recommended for UFs [32]. Systematic reviews and a meta-analysis are at the top of the clinical evidence hierarchy [33]. However, there is no clinical evidence to evaluate the safety and efficacy of GZFL for UFs in the current evidence-based medical studies. Therefore, a meta-analysis of the safety and efficacy of GZFL supplementation in patients with UFs is critical. Recently, an increasing number of high-quality randomized controlled trials have documented the safety and efficacy of combined GZFL and MFP regimens for the treatment of UFs. However, the sample sizes of these trials are generally small, and results based on small sample data make it difficult to convince the public that GZFL is significantly effective in the treatment of patients with UFs, which has limited the use and promotion of GZFL to some extent. Therefore, we conducted a high-quality, large-sample systematic review and meta-analysis to evaluate the efficacy and safety of randomized controlled trials of GZFL combined with low-dose MFP in the treatment of UFs. It is expected to provide a research basis for clinical practice.

**Table 1:** The composition of GZFL

Study	Ingredients of herb prescription	Source	Quality control Reported? (Y/N)	Chemical analysis reported (Y/N)
All the included studies	*Cinnamomum cassia* (L.) J.Presl*,* Lauraceae (240 g); *Paeonia* × *suffruticosa* Andrews, Paeoniaceae (240 g); *Poria cocos* (Schw.) Wolf, Polyporaceae (240 g); *Paeonia lactiflora* Pall., Paeoniaceae (240 g); *Prunus persica* (L.) Batsch or *Prunus davidiana* (CarriŠre) Franch., Rosaceae (240 g)	Jiangsu Kangyuan Pharmaceutical Co., Ltd	Y-According to Chinese Pharmacopoeia	Y-HPLC

## Materials and methods

### Protocol registration

This study was conducted by the Preferred Reporting Items for Systematic Reviews and Meta-Analyses (PRISMA) 2020 Statement, and a Measurement Tool to Assess Systematic Reviews 2 (AMSTAR 2) [34, 35]. The PRISMA 2020 checklist is provided in Supplementary Material S1. The overall methodological quality of this meta-analysis was high, as evidenced by the AMSTAR 2 assessment form in Supplementary Material S2. This study has been registered in PROSPERO (https://www.crd.york.ac.uk/prospero/), registration number: CRD42022326351. We do not collect any primary personal data; therefore, ethical approval is not required.

### Search strategy

We searched a total of 8 databases, including PubMed, Embase, Cochrane Library, Web of Science, China National Knowledge Infrastructure (CNKI), Wanfang, Chinese Scientific Journals Database (VIP), and Chinese Biological Medical Database (CBM). The retrieval time of each database is from the establishment to April 24, 2022, with no language and release status restrictions. The ClinicalTrials.gov database and Chinese Clinical Trial Registry (CHiCTR) were also searched from creation to April 24, 2022, for ongoing or unpublished clinical trials. The detailed retrieval strategy is provided in Supplementary Material S3.

### Inclusion and exclusion criteria

#### Types of studies

We only included RCTs that studied GZFL in combination with low-dose MFP in UFs, regardless of publication status or language. If we found relevant studies with three treatment groups, only data involving the GZFL and low-dose MFP groups were extracted. We excluded quasi-randomized trials, including studies in which participants were assigned sequentially by date of birth and admission number.

#### Types of participants

Eligible studies included adults (18 years and older) with subjects diagnosed with UFs. There were no restrictions on the course, severity, race, and age of the participant’s disease.

#### Types of interventions

In traditional Chinese medicine (TCM), there are significant differences in the formulations of different medicines for treating diseases. In order to reduce the influence of different drugs in TCM on the results, the composition of GZFL in our included studies must be unmodified 5 drugs. Authors should report the composition of the drugs they use or report any data that can be queried about the composition of the drug. According to the Chinese expert consensus on the diagnosis and treatment of uterine fibroids and the recommendations of gynecological experts, we only included studies in which the dose of MFP was 2.5–12.5 mg/day, and there was no restriction on the dose of GZFL; regardless of the administration time, administration How is the way. In the included studies, all patients in the combination treatment group were GZFL combined with low-dose MFP, and the control group was treated with MFP alone. The MFP used in the combination treatment group and the control group must be the same (such as administration dose, administration time, etc.).

#### Types of outcome measures

- 1) Primary Outcome: Clinical Efficiency Rate (CER): The sum of the percentage reduction in uterine fibroids in patients who achieved complete or partial remission. Fibroids are considered effective when they shrink by at least 25 percent; Adverse drug reactions (ADR): Any adverse events; Uterine Fibroids Volume (UFV), Uterine Volume (UV).
- 2) Secondary Outcome: Estradiol (E_2_), Progesterone (P), Follicle Stimulating Hormone (FSH), Luteinizing Hormone (LH), Menstrual Flow (MF).Outcomes of included studies included at least one primary outcome and one secondary outcome.
  Outcomes of included studies included at least one primary outcome and one secondary outcome.

#### Exclusion criteria

1) Conference abstracts, reviews, and in vitro and animal studies; 2) Duplicate publications; 3) Chinese non-herbal remedies such as acupuncture, cupping, or acupoint application are excluded; 4) Studies with incomplete data: the target results cannot be obtained without reporting or contacting the authors; 5) Other dosage forms of GZFL are excluded (such as decoction, pill, etc.).

## Data collection and analysis

### Research selection

We imported the literature results into the NoteExpress 3.6.0 software, and two authors (YL and LY) first deduplicated the imported literature and then evaluated potentially eligible articles by reading titles and abstracts to remove irrelevant studies or RCTs outside the inclusion criteria. Studies that met the inclusion criteria were obtained for further screening. Any disagreements between the two authors were resolved by discussion with the third author (YB).

### Data extraction

Data extraction was performed independently by two authors (YL and FG), and a third author (LY) reviewed the results of the extracted data. Based on research needs, we produced a standard table recording general information for each study: first author, year of publication, study design, participant age (Treatment/Control; years), number of participants (Treatment/control), details of GZFL combined with low-dose MFP group and low-dose MFP group (such as route of administration, dosage, etc.), treatment duration, funding, and outcomes. If necessary, we contacted study authors by email for additional unpublished information.

### Risk of bias assessment

A risk of bias assessment was performed using the 2019 Cochrane Randomized Trials Risk of Bias Tool 2.0 (RoB2.0) [36]. This risk of bias assessment includes the following five domains: bias arising from the randomization process, bias arising from deviations from the intended intervention, bias arising from the omission of outcome data, bias in measuring outcomes, bias in choosing to report outcomes, and Overall risk of bias judgment. Any disagreements are discussed with the third author (LY).

### Quality assessment of the evidence

The quality of the evidence was assessed using the GRADE criteria [37] according to the website (https://www.gradepro.org/). The quality of evidence for meta-analysis results was rated as very low, low, moderate, or high. Initially, the RCT results were classified as high-quality evidence. The quality of each result can decrease due to factors such as the risk of bias, imprecision, inconsistency, indirectness, and publication bias. GRADE pro3.6.1 software was used for data analysis and synthesis.

### Data synthesis

Data synthesis was performed using the Meta package (version 4.11) of RStudio (https://www.rstudio.com/) and RevMan 5.4. For dichotomous data, relative risk (RR) and 95% confidence intervals were used. For continuous data, mean difference (MD) and 95%, CI were used to represent effect sizes when the same units were used for the same outcome indicators; otherwise, standardized mean difference (SMD) and 95% CI were used.

Heterogeneity was evaluated with the *χ*2 test and the *I*^2^ test. If *p* > 0.1, *I*^*2*^ < 50%, the heterogeneity between studies was small and the fixed effects model was used to calculate the pooled effect size. If *p* ≤ 0.1, *I*^*2*^ ≥ 50%, this suggests statistically significant heterogeneity among studies; therefore, a random-effects model was used. Subgroup analysis was used to explore the sources of heterogeneity. The subgroup analysis was according to the age of UFs patients, the dosage of GZFL, the dosage of MFP, and the treatment period. We performed a sensitivity analysis to test the stability of the pooled outcome data. In addition, when the number of trials for a pooled outcome measure was ≥ 10, we used funnel plots to analyze the publication bias of included studies; we also used Egger’s test and Peter’s test to examine the effect of publication bias. For all results, data were considered statistically significant at *p* < 0.05.

## Results

### Database search

**Fig. 1:**
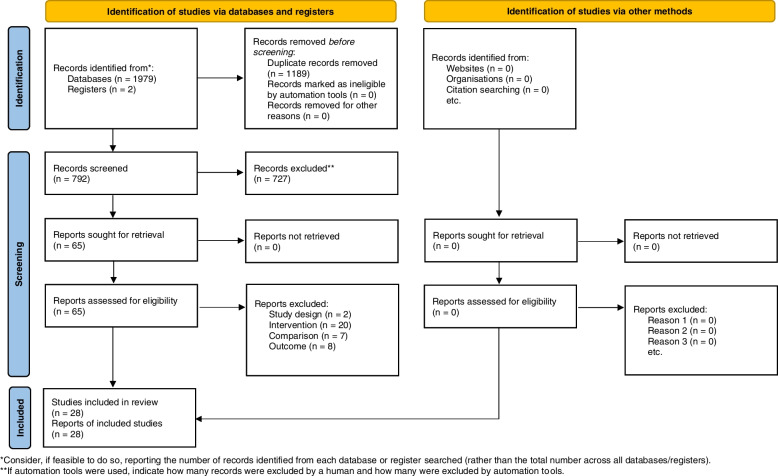
Flow diagram of study selection and identification

A total of 1981 citations were retrieved from relevant databases and clinical trial registries. 1189 citations were excluded due to duplication. After reading the title and abstract, 727 citations were excluded. After reading the full text, 37 citations were excluded. No other publications were found by searching references, relevant reviews, and meta-analyses of included studies. Finally, 28 studies were included for quantitative analysis [38–65]. Detailed reasons for excluding citations are provided in Supplementary Material S4. A flowchart of study selection is shown in Fig. 1.

### Characteristics of the included studies

Baseline characteristics of all included studies are shown in Table 2. All studies were randomized controlled trials. A total of 28 trials including 2813 patients participated in this study [54–81], Among them, the experimental group (*n* = 1407) and the control group (*n* = 1406). Patients ranged in age from 35 to 46 years, treatment cycles ranged from three to six months, and four studies reported follow-up, all for six months.

**Table 2:** The characteristics of the included studies

Study	Sample(T/C)	Study design	Age (T/C, years)	Interventions		Duration	Follow-up	Outcomes	Funding
				Treatment	Comparator				
Cao (2016) [38]	40/40	RCT	37.1 ± 2.5/36.8 ± 2.3	GZFL (3.72 g/d, qid) + MFP (12.5 mg/d, qd)	MFP(12.5 mg/d, qd)	3 months	NR	CER, FSH, LH, E_2_, P	NR
Chen et al. (2008) [39]	35/35	RCT	36.4 ± 4.2/38.3 ± 5.1	GZFL (3.72 g/d, qid) + MFP (12.5 mg/d, qd)	MFP(12.5 mg/d, qd)	3 months	6 months	CER, UFV, FSH, LH, E_2_, P, ADR	NR
Deng and Li (2010) [40]	33/36	RCT	38.9 ± 8.1/39.5 ± 8.9	GZFL (2.79 g/d, tid) + MFP (12.5 mg/d, qd)	MFP(12.5 mg/d, qd)	3 months	6 months	CER, UFV, UV, FSH, LH, E_2_, P, ADR	NR
Fei (2017) [41]	39/39	RCT	34.19 ± 5.65/35.69 ± 6.35	GZFL (3.72 g/d, qid) + MFP (12.5 mg/d, qd)	MFP(12.5 mg/d, qd)	3 months	NR	CER, UFV, UV, FSH, LH, E_2_, P	NR
Gu and Hu (2011) [42]	67/67	RCT	34.2 ± 7.2/35.5 ± 6.6	GZFL (3.72 g/d, qid) + MFP (12.5 mg/d, qd)	MFP(12.5 mg/d, qd)	3 months	NR	CER, UFV, E_2_, P	NR
Hu (2013) [43]	69/69	RCT	43 ± 9/43 ± 8	GZFL (2.79 g/d, tid) + MFP (12.5 mg/d, qd)	MFP(12.5 mg/d, qd)	3 months	NR	CER, FSH, LH, E_2_, P	NR
Li and Gao (2015) [44]	135/135	RCT	37.2/37.4 (years)	GZFL (3.72 g/d, qid) + MFP (12.5 mg/d, qd)	MFP(12.5 mg/d, qd)	3 months	NR	CER, UFV, UV, FSH, LH, E_2_, P, ADR	NR
Li (2017) [45]	50/50	RCT	37.2 ± 4.1/36.7 ± 3.9	GZFL (2.79 g/d, tid) + MFP (12.5 mg/d, qd)	MFP(12.5 mg/d, qd)	4 months	NR	CER, UFV, UV, FSH, LH, E_2_, ADR	NR
Liang (2017) [46]	49/49	RCT	37.31 ± 5.19	GZFL (3.72 g/d, qid) + MFP (10 mg/d, qd)	MFP(10 mg/d, qd)	3 months	NR	CER, UFV, UV, FSH, LH, E_2_, P	NR
Lin (2019) [47]	44/43	RCT	36.5 ± 5.0/36.0 ± 5.0	GZFL (2.79 g/d, tid) + MFP (12.5 mg/d, qd)	MFP(12.5 mg/d, qd)	3 months	NR	CER, UFV, FSH, LH, E_2_, P	NR
Liu (2016) [48]	59/59	RCT	34.8 ± 4.2/35.5 ± 4.7	GZFL (2.79 g/d, tid) + MFP (12.5 mg/d, qd)	MFP(12.5 mg/d, qd)	6 months	NR	CER, UFV, UV, FSH, LH, E_2_, ADR, MF	NR
Luo (2012) [49]	39/39	RCT	43.38 ± 4.69/43.33 ± 4.78	GZFL (3.72 g/d, qid) + MFP (10 mg/d, qd)	MFP (10 mg/d, qd)	3 months	NR	CER, FSH, LH, E_2_, P	NR
OU and Lan (2015) [50]	35/35	RCT	36.21 ± 6.19/36.55 ± 6.35	GZFL (2.79 g/d, tid) + MFP (12.5 mg/d, qd)	MFP (12.5 mg/d, qd)	4 months	NR	CER, UFV, UV, FSH, LH, E_2_, ADR	NR
Qin (2015) [54]	54/54	RCT	41.9 ± 6.2/43.1 ± 5.8	GZFL (3.72 g/d, qid) + MFP (10 mg/d, qd)	MFP (10 mg/d, qd)	3 months	NR	CER, FSH, LH, E_2_, P	NR
Sha and Zhu (2016) [51]	40/40	RCT	45 ± 1.4/44 ± 1.5	GZFL (3.72 g/d, qid) + MFP (12.5 mg/d, qd)	MFP (12.5 mg/d, qd)	6 months	6 months	CER, UFV, FSH, LH, E_2_, P	NR
Si et al. (2019) [52]	50/50	RCT	38.76 ± 3.80/38.99 ± 3.46	GZFL (2.79 g/d, tid) + MFP (12.5 mg/d, qd)	MFP (12.5 mg/d, qd)	3 months	NR	CER, UFV, UV, FSH, LH, E_2_, P	Henan Province Traditional Chinese Medicine Scientific Research Special Project (2015ZY02108)
Su (2013) [53]	61/61	RCT	34.6 ± 6.8	GZFL (3.72 g/d, qid) + MFP (12.5 mg/d, qd)	MFP (12.5 mg/d, qd)	3 months	NR	CER, UFV, FSH, LH, E_2_, P	NR
Wang and Zhang (2021) [55]	41/41	RCT	38.97 ± 2.88/38.95 ± 2.86	GZFL (2.79 g/d, tid) + MFP (12.5 mg/d, qd)	MFP (12.5 mg/d, qd)	3 months	NR	CER, UFV, FSH, LH, E_2_, P, ADR, MF	NR
Wei (2019) [57]	50/50	RCT	40.32 ± 0.58/40.03 ± 0.13	GZFL (2.79 g/d, tid) + MFP (12.5 mg/d, qd)	MFP (12.5 mg/d, qd)	3 months	NR	CER, FSH, E_2_	NR
Wei (2014) [58]	43/43	RCT	35.3 ± 4.2/33.8 ± 6.8	GZFL (2.79 g/d, tid) + MFP (12.5 mg/d, qd)	MFP (12.5 mg/d, qd)	3 months	NR	CER, FSH, LH, E_2_, P	NR
Xu (2017) [59]	70/70	RCT	38.42 ± 3.85/38.76 ± 3.92	GZFL (3.72 g/d, qid) + MFP (12.5 mg/d, qd)	MFP (12.5 mg/d, qd)	3 months	NR	CER, FSH, LH, E_2_	NR
Yang (2018) [60]	54/54	RCT	40.1 ± 2.3/40.3 ± 2.5	GZFL (2.79 g/d, tid) + MFP (10 mg/d, qd)	MFP (10 mg/d, qd)	3 months	NR	CER, UFV, FSH, LH, E_2_, P, ADR	NR
Yuan et al. (2021) [61]	54/54	RCT	40.02 ± 2.94/39.22 ± 3.86	GZFL (2.79 g/d, tid) + MFP (10 mg/d, qd)	MFP (10 mg/d, qd)	3 months	NR	CER, UFV, FSH, LH, E_2_, P	NR
Zhang and Yi (2014) [62]	36/36	RCT	39.6 ± 4.2	GZFL (3.72 g/d, qid) + MFP (10 mg/d, qd)	MFP (10 mg/d, qd)	3 months	NR	CER, UFV, UV, FSH, LH, E_2_, P, ADR	NR
Zhang et al. (2019) [63]	42/42	RCT	39.53 ± 7.15/38.13 ± 6.15	GZFL (2.79 g/d, tid) + MFP (10 mg/d, qd)	MFP (10 mg/d, qd)	3 months	NR	CER, UFV, UV, E_2_, P, ADR	NR
Zhong (2020) [64]	26/26	RCT	42.5 ± 12.5/43.5 ± 11.5	GZFL (2.79 g/d, tid) + MFP (12.5 mg/d, qd)	MFP (12.5 mg/d, qd)	3 months	NR	CER, UFV, FSH, LH, E_2_, ADR, MF, HE4	NR
Zhou (2016) [65]	28/27	RCT	36.54 ± 3.27	GZFL (3.72 g/d, qid) + MFP (12.5 mg/d, qd)	MFP (12.5 mg/d, qd)	3 months	NR	CER, E_2_, P	NR
Wang (2004) [56]	64/62	RCT	40–55/41–53	GZFL (2.79 g/d, tid) + MFP (12.5 mg/d, qd)	MFP (12.5 mg/d, qd)	6 months	6 months	CER, FSH, LH, E_2_, P	NR

*T* Treatment group, *C* Control group, *NR* Not report, *GZFL* Guizhi fuling capsule, *MFP* Mifepristone, *t.i.d* three times a day, *qd* One a day, *CER* Clinical efficiency rate, *ADR* Adverse drug reactions, *UFV* Uterine fibroids volume, *UV* Uterine volume, *E*_*2*_ Estradiol, *P* Progesterone, *FSH* Follicle stimulating hormone, *LH* Luteinizing hormone, *MF* Menstrual flow

### Risk of bias assessment

**Fig. 2:**
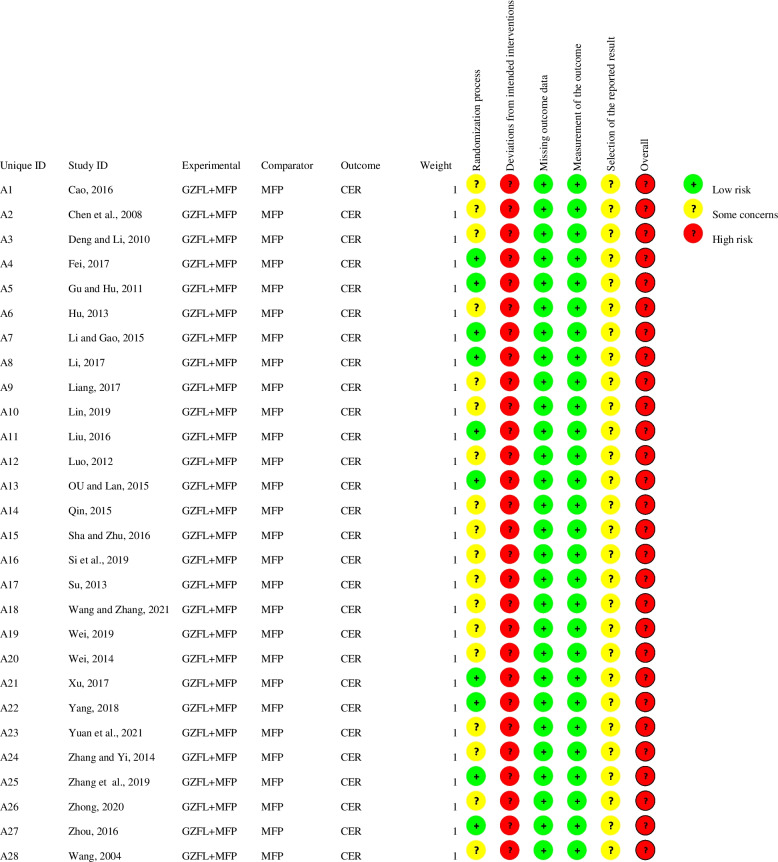
Risk-of-bias graph

**Fig. 3:**
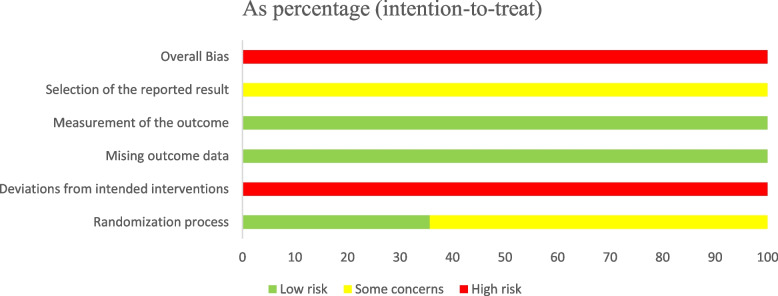
Risk-of-bias summary

All 28 included studies were judged to be at moderate risk of bias. The results of the risk of bias assessment are shown in Figs. 2 and 3, and details are provided in Supplementary Material S5.

### Primary outcome measures

#### Clinical efficiency rate

**Fig. 4:**
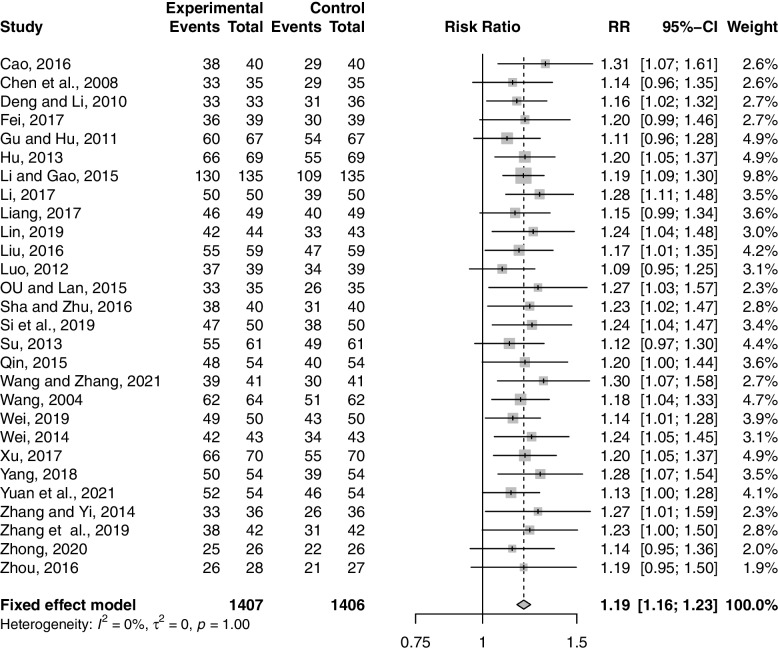
Forest plot of the clinical efficiency rate

Twenty-eight studies including 2813 participants, reported the effect of combination therapy compared with MFP alone [38–65] on clinical efficacy rate. The heterogeneity of the pooled analysis was low (*p* = 1.00, *I*^*2*^ = 0%), so a fixed-effects model was used for the meta-analysis. The results showed that the combined treatment significantly improved the clinical efficacy rate (RR = 1.19; 95% CI, 1.16 to 1.23; *p* < 0.0001; *I*^*2*^ = 0%) (Fig. 4). Sensitivity analysis showed that the results were robust (Supplementary Material S6.1). Subgroup analysis based on age, GZFL dose, MFP dose, and treatment duration showed that there was no significant interaction between these factors (Supplementary Material S6.2-S6.5).

#### UFV

**Fig. 5:**
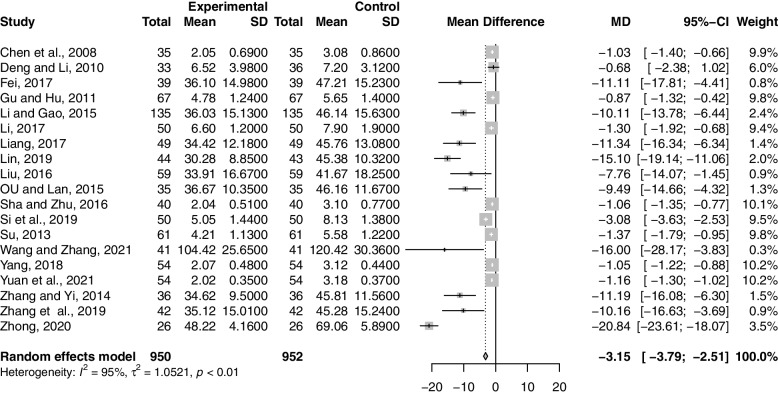
Forest plot of uterine fibroids volume

Nineteen studies including 1902 participants reported the effect of combination therapy on UFV compared with MFP alone [39–42, 44–48, 50–53, 55, 60–64]. The heterogeneity of the pooled analysis was high (*p* < 0.01, *I*^*2*^ = 91%), so a random-effects model was used for the meta-analysis. Pooled results showed that combination therapy reduced UFV (MD = -3.15; 95% CI, -3.79 to -2.51; *p* < 0.0001) (Fig. 5). Sensitivity analysis showed that the results were robust (Supplementary Material S6.6). Due to the large heterogeneity in the studies, we performed subgroup analyses according to age, GZFL dose, MFP dose, and duration of treatment. However, subgroup analysis showed that these factors had no significant interaction (Supplementary Material S6.7-S6.10).

#### UV

**Fig. 6:**
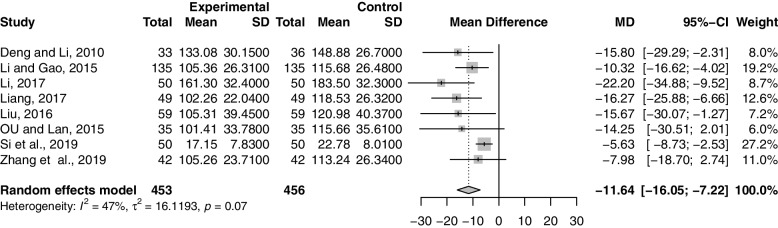
Forest plot of uterine volume

Eight studies including 909 participants reported the effect of combination therapy on UV compared with MFP alone [40, 44–46, 48, 50, 52, 63]. The data were heterogeneous (*p* = 0.07, *I*^*2*^ = 47%), so a random-effects model was used for the meta-analysis. Pooled results showed that combination therapy reduced UV (MD = -11.64; 95% CI, -16.05 to -7.22; *p* < 0.0001) (Fig. 6). Sensitivity analysis showed that the heterogeneity was reduced to 0% when [52] were excluded, but interestingly this did not reverse our conclusion that the result of combined treatment in reducing UV was robust (Supplementary Material S6.11). We reviewed the full text again and concluded that the main source of reduced heterogeneity may be differences in pre-treatment UV. Improvement in UV with combination therapy may be closely related to disease severity. After excluding [52], we performed subgroup analysis according to GZFL dose, MFP dose, and duration of treatment. However, subgroup analysis showed that these factors had no significant interaction (Supplementary Material S6.12-S6.15).

#### ADR

**Fig. 7:**
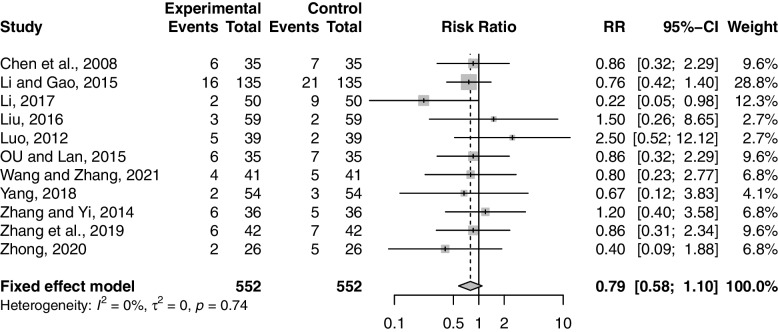
Forest plot of adverse drug reactions

Eleven studies including 1104 participants reported the effect of combination therapy on ADR compared with MFP alone [39, 44, 45, 48–50, 55, 60, 62–64]. The heterogeneity of the pooled analysis was low (*p* = 0.74, *I*^*2*^ = 0%), so a fixed-effects model was used for the meta-analysis. Pooled results showed that combination therapy did not increase the incidence of ADR (RR = 0.79; 95% CI, 0.58 to 1.10; *p* = 0.16) (Fig. 7). Sensitivity analysis showed that the results were robust (Supplementary Material S6.16). Subgroup analyses were performed according to age, GZFL dose, MFP dose, and duration of treatment. Subgroup analysis showed that these factors had no significant interaction (Supplementary Material S6.17-S6.20).

### Secondary outcomes

#### FSH

**Fig. 8:**
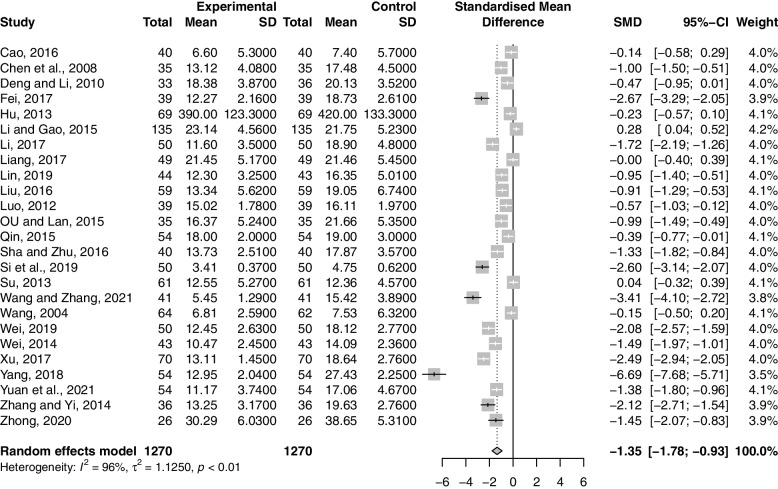
Forest plot of follicle stimulating hormone

Twenty-five studies including 2540 participants reported the effect of combination therapy on FSH compared with MFP alone [38–41, 43–62, 64]. The heterogeneity of the pooled analysis was high (*p* < 0.01, *I*^*2*^ = 96%), so a random-effects model was used for the meta-analysis. The results showed that combination therapy reduced FSH (SMD = -1.35; 95% CI, -1.78 to -0.93; *p* < 0.0001) (Fig. 8). Sensitivity analysis showed that the results were robust (Supplementary Material S6.21). Due to the large heterogeneity in the studies, we performed subgroup analyses according to age, GZFL dose, MFP dose, and treatment duration. However, subgroup analysis showed that these factors had no significant interaction (Supplementary Material S6.22-S6.25).

#### E_2_

**Fig. 9:**
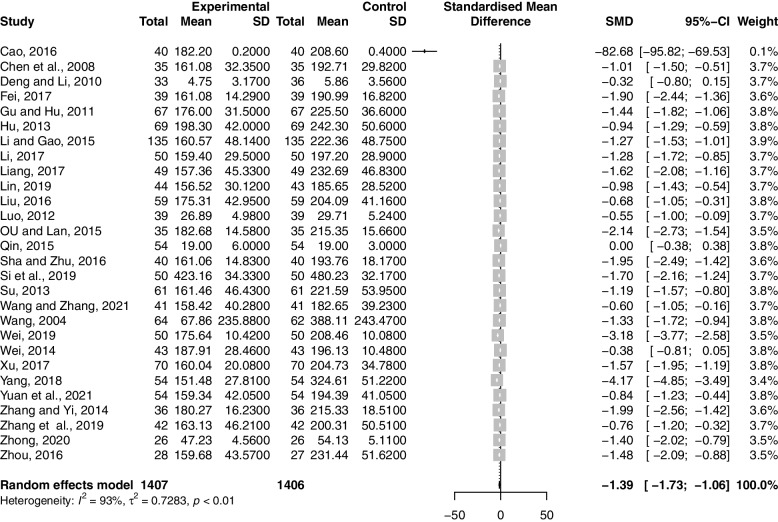
Forest plot of estradiol

Twenty-eight studies including 2813 participants reported the effect of combination therapy on E_2_ compared with MFP alone [38–65]. The heterogeneity of the pooled analysis was high (*p* < 0.01, *I*^*2*^ = 93%), so a random-effects model was used for the meta-analysis. Pooled results showed that combination therapy reduced E_2_ (SMD = -1.39; 95% CI, -1.73 to -1.06; *p* < 0.0001) (**Fig. ****9**). Sensitivity analysis showed that the results were robust (Supplementary Material S6.26). Due to the large heterogeneity in the studies, we performed subgroup analyses according to age, GZFL dose, MFP dose, and duration of treatment. However, subgroup analysis showed that these factors had no significant interaction (Supplementary Material S6.27-S6.30).

#### P

**Fig. 10:**
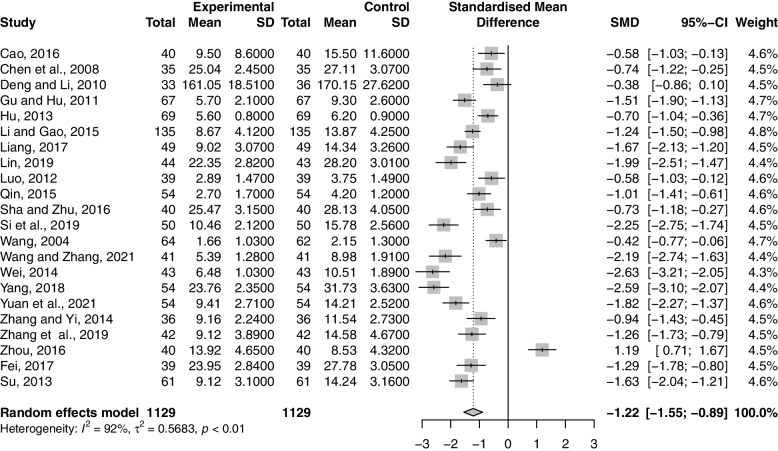
Forest plot of progesterone

Twenty-two studies including 2258 participants reported the effect of combination therapy on P compared with MFP alone [38–44, 46, 47, 49, 51–56, 58, 60–63, 65]. The heterogeneity of the pooled analysis was high (*p* < 0.01, *I*^*2*^ = 92%), so a random-effects model was used for the meta-analysis. Pooled results showed that combination therapy reduced P (SMD = -1.22; 95% CI, -1.55 to -0.89; *p* < 0.0001) (Fig. 10). Sensitivity analysis showed that the results were robust (Supplementary Material S6.31). Due to the large heterogeneity in the studies, we performed subgroup analyses according to age, GZFL dose, MFP dose, and duration of treatment. However, subgroup analysis showed that these factors had no significant interaction (Supplementary Material S6.32-S6.35).

#### LH

**Fig. 11:**
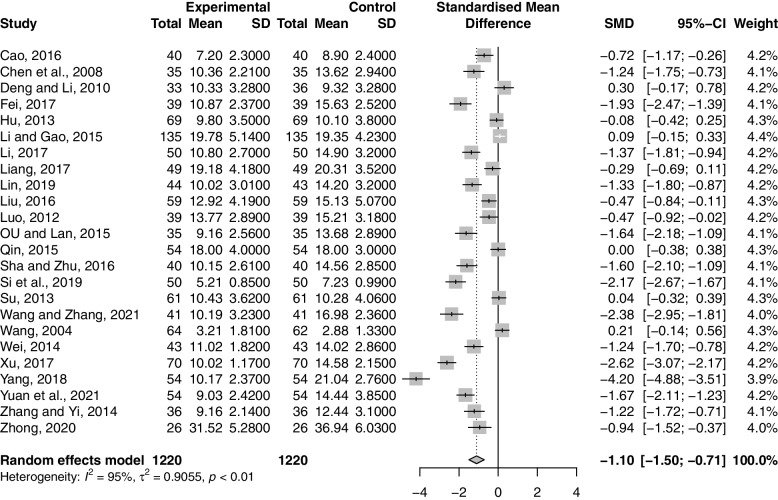
Forest plot of luteinizing hormone

Twenty-four studies including 2440 participants reported the effect of combination therapy on LH compared with MFP alone [38–41, 43–56, 58–62, 64]. The heterogeneity of the pooled analysis was high (*p* < 0.01, *I*^*2*^ = 95%), so a random-effects model was used for the meta-analysis. Pooled results showed that combination therapy reduced LH (SMD = -1.10; 95% CI, -1.50 to -0.71; *p* < 0.0001) (Fig. 11). Sensitivity analysis showed that the results were robust (Supplementary Material S6.36). Due to the large heterogeneity in the studies, we performed subgroup analyses according to age, GZFL dose, MFP dose, and duration of treatment. However, subgroup analysis showed that these factors had no significant interaction (Supplementary Material S6.37-S6.40).

#### MF

**Fig. 12:**
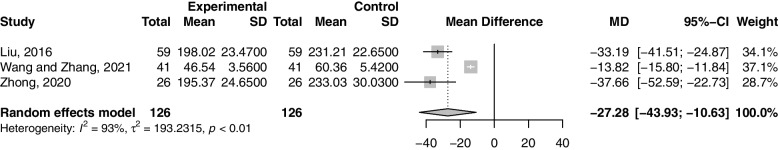
Forest plot of menstrual flow

Three studies including 252 participants reported the effect of combination therapy on MF compared with MFP alone [48, 55, 64]. The heterogeneity of the pooled analysis was high (*p* < 0.01, *I*^*2*^ = 93%), so a random-effects model was used for the meta-analysis. Pooled results showed that combination therapy reduced MF (MD = -27.28; 95% CI, -43.93 to -10.63; *p* = 0.0013) (Fig. 12). Sensitivity analysis showed that when [55] were excluded, the heterogeneity was reduced to 0%, but interestingly this did not reverse our conclusion that the results of combined treatment in reducing MF were robust (Supplementary Material S6.41). We reviewed the full text again and concluded that the main source of reduced heterogeneity may be differences in pre-treatment menstrual flow.

#### Publication bias

In this meta-analysis, we used funnel plots, Egger’s test, and Peter’s test to examine publication bias for CER, FSH, E_2_, P, LH, UFV, and ADR. The symmetrical shape of the funnel plot, and the *p*-values for Egger’s and Peter’s tests, revealed no significant publication bias for CER, ADR, and P (*p* = 0.26; *p* = 0.91; *p* = 0.28). However, FSH, E_2_, LH, and UFV may have publication bias (*p* < 0.001), and trim and fill were used to analyze the effect of publication bias on pooled results. (Supplementary Material S6.42- S6.52).

#### GRADE assessment

The quality of the evidence for the outcomes ranged from “very low” to “moderate”. The reasons for the downgrade were the flawed methodology of the selected studies, high risk of bias, inconsistent results due to significant heterogeneity, and imprecise results due to wide confidence intervals and small sample sizes (Table 3).

**Table 3:** Results of meta-analysis and quality of evidence

Outcomes	No. participants (RCTs)	Anticipated absolute effects (95% CI)		Relative effect (95% CI)	*I*^*2*^ value	Quality of evidence (GRADE)	Comments
		**Risk with MFP group**	**Risk with GZFL + MFP group**				
Clinical Efficiency Rate	2813 (28)	791 per 1000	941 per 1000 (917 to 973)	RR 1.19 (1.16 to 1.23)	0	⨁⨁⨁◯ MODERATE	Risk of bias (-1) ^a^
Uterine Fibroids Volume	1902 (19)	-	MD 3.15 lower (3.79 to 2.51 lower)	-	95	⨁◯◯◯ VERY LOW	Risk of bias (-1) ^a^ Inconsistency (-1) ^b^ Publication bias (-1) ^c^
Uterine Volume	909 (8)	-	MD 8.56 lower (10.99 to 6.12 lower)	-	47	⨁⨁⨁◯ MODERATE	Risk of bias (-1) ^a^
Adverse drug reactions	1104 (11)	132 per 1000	104 per 1000 (77 to 145)	RR 0.79 (0.58 to 1.1)	0	⨁⨁◯◯ LOW	Risk of bias (-1) ^a^ Imprecision (-1) ^d^
Estradiol	2813 (28)	-	SMD 1.39 lower (1.73 to 1.06 lower)	-	93	⨁◯◯◯ VERY LOW	Risk of bias (-1) ^a^ Inconsistency (-1) ^b^ Publication bias (-1) ^c^
Progesterone	2258 (22)	-	SMD 1.22 lower (1.55 to 0.89 lower)	-	92	⨁⨁◯◯ LOW	Risk of bias (-1) ^a^ Inconsistency (-1) ^b^
Follicle Stimulating Hormone	2540 (25)	-	SMD 1.35 lower (1.78 to 0.93 lower)	-	96	⨁◯◯◯ VERY LOW	Risk of bias (-1) ^a^ Inconsistency (-1) ^b^ Publication bias (-1) ^c^
Luteinizing Hormone	2440 (24)	-	SMD 1.1 lower (1.5 to 0.71 lower)	-	95	⨁◯◯◯ VERY LOW	Risk of bias (-1) ^a^ Inconsistency (-1) ^b^ Publication bias (-1) ^c^
Menstrual Flow	252 (3)	-	MD 27.28 lower (43.93 to 10.63 lower)	-	93	⨁⨁◯◯ LOW	Risk of bias (-1) ^a^ Inconsistency (-1) ^b^

*GZFL* Guizhi Fuling capsule, *MFP* Mifepristone, *CI* Confidence interval, *RR* Relative risks, *MD* Mean difference, *SMD* Standardized mean difference

^a^ Most studies lack allocation concealment and blinding

^b^ Heterogeneity (I^2^ > 50%, *p* < 0.05) was found

^c^ Publication bias

^d^ wide confidence intervals

## Discussion

With the increasing incidence of UFs, the cost of treatment plays a crucial role in the world’s health and economic burden [4, 66–68]. However, current treatment options are still unsatisfactory. Therefore, it is important to develop effective drugs or methods for the treatment of UFs. Traditional herbal complementary alternative therapies for UFs are receiving increasing attention. In China, Chinese herbal medicine has a long history of use as an adjunctive treatment for UFs and is recognized by clinicians [69]. GZFL capsules are a traditional herbal medicine for the treatment of UFs and they are effective. Therefore, the purpose of this meta-analysis was to evaluate the clinical efficacy and safety of GZFL in the treatment of UFs.

### Main results of this research

This study is the first systematic review and meta-analysis of the efficacy and safety of GZFL combined with low-dose MFP in the treatment of UFs. We performed a comprehensive literature search to include and analyze 28 randomized controlled trials including 2813 patients with UFs. Providing evidence-based guidelines and recommendations based on research findings is critical to clinical decision-making, so the findings of systematic reviews and meta-analyses are meaningful. Under normal circumstances, treatment of UFs is usually given priority to improving the size of UFs, and safety evaluation are equally important, while changes in hormones and MF can reflect whether disease control is stable. Therefore, in this study, CER, ADR, UV, and UFV were used as the primary outcome, and FSH, LH, P, E2, and MF were used as the secondary outcome. The above indicators were used as clinical trial observation and efficacy evaluation indicators. Our results show that GZFL combined with low-dose MFP has advantages in reducing FSH, E2, P, LH, UFV, UV, and MF and improving the CER in patients with UFs, and the sensitivity analysis also supports this result.

The evidence presented here suggests that GZFL combined with low-dose MFP is superior to MFP alone, supporting an additional role for GZFL in the treatment of UFs. GZFL combined with low-dose MFP may have the potential to be developed as a new standard combination therapy, complementing existing international guidelines for UFs. In addition, to discuss the effects of GZFL and MFP administration dose, treatment duration, and patient age on the treatment of UFs, we performed subgroup analyses. Interestingly, the results showed that GZFL combined with low-dose MFP improved CER and decreased FSH, E2, P, LH, UFV, UV, and MF, regardless of dose, treatment time, or patient age. The benefits persist. However, in this systematic review, three studies analyzed MF in patients and although the results were positive, future studies still need to focus on its validity due to the small number of participants included. Meanwhile, FSH, E2, P, LH, and UFV were limited by undetermined heterogeneity in our study, and this uncertainty may affect the clinical application of our results. Therefore, identifying sources of heterogeneity among study results is of concern. However, the source of heterogeneity could not be identified by subgroup analysis of dose, duration of treatment, and age of patients. From an analysis of the data we found that, in most of the studies we included, data on the type of UFs (intramural myoma, subserous myoma, submucous myoma, mixed myoma) and pre-treatment fibroids were missing Data on the volume of UFs. Pre-treatment disease severity had a significant impact on the efficacy of treatment, therefore, we speculated that pre-treatment fibroid volume and fibroid type might be one of the reasons for the unresolved heterogeneity. In addition, the determination of biochemical results is susceptible to different factors. At the same time, not all included studies reported random assignment generation and assignment concealment. The blind method has not been reported. This may also be a source of unresolved heterogeneity, and more high-quality RCTs are needed in the future to address this.

The results of the clinical safety evaluation showed that there was no significant difference in the incidence of ADR between the two groups, and there was no obvious liver toxicity and other side effects mentioned in previous reports of MFP treatment of UFs [70], suggesting that GZFL combined with low-dose MFP in the treatment of UFs patients is relatively safe. Meanwhile, we also conducted a subgroup analysis of ADR, and the results showed that there was no significant interaction between ADR and administration dose, treatment time, or patient age. Therefore, we provide supportive evidence that, to a large extent, GZFL may be recommended for planned use in patients with UFs.

Finally, publication bias testing revealed possible publication bias in FSH, E2, LH, and UFV, and FSH, E2, LH, and UFV were treated for trimming and filling. The results indicated that E2 and UFV were statistically significant before and after repair, meaning that publication bias had no significant effect on the pooled results and the results were robust and credible. FSH and LH were not statistically significant before and after repair, indicating that the results were not robust and that further research is needed. Due to the small number of studies reporting UV and MF, we did not perform a publication bias test for these results. Given the low statistical power of funnel plots, Egger’s test, and Peter’s test, and the fact that none of the original trials provided information on clinical trial registration, we still cannot completely rule out the possibility of publication bias.

### Comparison with previous studies

Several systematic reviews and meta-analyses have demonstrated the efficacy and safety of Chinese herbal medicine in the treatment of UFs. A meta-analysis consisting of nine RCTs involving 844 patients showed that Chinese herbal medicine was safe and effective in reducing UFV and reducing ADR [71]. However, after TCM treatment, UV, E_2_, and P were superior to controls, which is inconsistent with our results. The results of our study showed that UV, E_2_, and P were all superior to the TCM group and that ADR was not statistically different between the two groups. Furthermore, most of our findings are consistent with the data from Shi et al. [72]. They conducted a meta-analysis involving 11 trials focusing on 902 patients and showed that TCM combined with MFP treatment significantly reduced UFV, and UV as well as improved CER. It also reduced FSH, LH, E_2_, and P levels in treated patients. However, the analysis of symptom indicators related to UFs was not mentioned in their study, and the low ADR in the TCM group was not consistent with our findings. Also, the results of the subgroup analysis of FSH and E_2_ showed no significant difference between the two groups at 6 months, whereas our results suggested better efficacy than the TCM group at 6 months. These contradictory findings may arise from differences in search strategies as well as selection criteria. The different TCM and control groups resulted in high heterogeneity, which may be the main reason for the contradictions.

### Strengths and limitations

This study is the first to evaluate the efficacy and safety of GZFL in the treatment of UFs. The methodological quality of this study was assessed using AMSTAR 2 and was determined to be of a high standard. In addition, we provide comprehensive supplementary materials that allow the work to be reproduced and reviewed. Impressively from our results, if patients with UFs cannot tolerate the effects of conventional therapy, they may have another option, a GZFL combined with a low-dose MFP regimen, to improve symptoms of UFs. At the same time, all the studies included in this study were randomized controlled trials with high quality and large quantities. This helps overcome the pitfalls that come with non-random and quasi-random research. In addition, the number of trials and total sample size included in this study was relatively large (28 trials, 2813 patients). Furthermore, we performed subgroup analyses to explore the possible impact of clinical and methodological heterogeneity on statistical heterogeneity, to identify sources of heterogeneity, and to explore how this difference might affect treatment. We also used funnel plots, Egger’s test, and Peter’s test to detect publication bias. Results with publication bias were also trimmed and filled in to see how this bias might affect the results of the study. Sensitivity analyses were performed to explore the degree of stability of the findings, which showed that the results of this meta-analysis were relatively stable.

Although comprehensive and high-quality techniques are employed, this study inevitably has certain limitations. First, although RCTs were included, the original studies included had some inherent and methodological shortcomings. Of these, only 12 trials provided sufficient information for random sequence generation. Blinding was not reported in the included studies, and although it may be difficult to achieve, this bias may challenge the findings. In future studies, proper quality control is necessary. In addition, UFs are chronic disease that requires long-term treatment, and the safety and efficacy of long-term medication are the keys to determining the clinical efficacy of drugs. However, the trials included in this study had treatment periods of 3 to 6 months, follow-up was only mentioned in individual trials, and the remaining trials did not investigate the long-term outcomes of GZFL. Therefore, we have not established the long-term safety of GZFL in our treatment of us. Second, although we did not restrict the language of included trials, the search strategy for screening potential studies was only used in English or Chinese databases; therefore, relevant studies published in other languages may be excluded, which may lead to some selection bias. In addition, all trials included in this study were conducted in China, but the frequency of UFs varies by ethnicity and region. For example, African-American women and African-European women have a higher risk of uterine fibroids than white women [1]. Based on these differences, it may be necessary to compare the efficacy of different ethnic groups and different regions in the future. Finally, the quality of the included clinical studies is of average quality, suggesting that high-quality multi-center RCTs of GZFL combined with low-dose MFP in the treatment of UFs needs to be further carried out on a global scale, to achieve the global promotion of the data and include more trustworthy clinical evidence, to support the rational use of GZFL.

### Implications for research

We raise expectations that may facilitate the development of research in this area. Firstly, improving the design of randomized controlled trial methodology, such as clarifying the generation of randomized sequences, allocation concealment, blinding, and the use of sample size estimates. Secondly, trial protocols should be registered in advance and posted on websites for use by researchers, to prevent duplication of studies and selective publication and reporting of expected study results. We recommend reporting randomized controlled trials on TCM by the CONSORT 2010 Statement [73]. We recommend that any adverse events regarding the course of the trial be closely monitored and recorded according to the standard reporting format to provide a basis for the safe use of TCM [74]. Patients should be followed up at the end of the clinical trial to assess and document the long-term efficacy of TCM. Studies should be evaluated in different countries and ethnicities to determine the broad applicability of GZFL. Although we attempted to evaluate each outcome included in the study with MD, we used SMD to evaluate these outcomes because the investigators used different units in the trial. However, these outcomes may have been overestimated or underestimated. Therefore, we recommend standardizing the units used to measure outcomes, which will facilitate the synthesis and comparison of data.

The results of the studies in our review show that GZFL has the potential to help improve certain outcomes in patients with UFs, providing clinical evidence for the effectiveness and safety of GZFL as a potential candidate for the management of UFs. However, the inclusion of GZFL in clinical practice guidelines for the management of UFs remains a challenge. Therefore, we recommend that clinicians give more consideration to this when prescribing. In addition, clinicians and patients should closely monitor medication use.

## Conclusion

The current evidence shows that GZFL combined with low-dose MFP has more advantages in improving the CER and reducing FSH, E2, P, LH, UFV, UV, and MF; GZFL combined with low-dose MFP does not increase the incidence of ADR, suggesting that GZFL is a potential treatment for UFs. However, given the low quality of the included studies and the large variability between eligible trials, we should approach the results with caution. In the future, multi-sample, multi-center, and high-quality RCT studies are still needed to prove this conclusion.

## Supplementary Information

**Additional file 1:** **Supplementary File S1.** PRISMA 2020 checklist. **Supplementary File S2.** AMSTAR 2 checklist. **Supplementary File S3.** Search strategies for databases. **Supplementary File S4.** Excluded studies and reasons for exclusion after reading the full text. **Supplementary File S5.** risk of bias assessment.

**Additional file 2:** **Supplementary File S6.** Results of subgroup analyses, sensitivity analyses and publication bias.

## Data Availability

The original contributions presented in the study are included in the article/Supplementary Material, further inquiries can be directed to the corresponding authors.

## Abbreviations

UFs: Uterine fibroids
MFP: Mifepristone
GZFL: Guizhi Fuling capsule
TCM: Traditional Chinese medicine
ADR: Adverse drug reactions
CER: Clinical efficiency rate
UFV: Uterine fibroids volume
UV: Uterine volume
MF: Menstrual flow
E_2_: Estradiol
P: Progesterone
FSH: Follicle stimulating hormone
LH: Luteinizing hormone
