# Supplementary material for: The efficacy and safety of Chinese herbal medicine Guizhi Fuling capsule combined with low dose mifepristone in the treatment of uterine fibroids: a systematic review and meta-analysis of 28 randomized controlled trials
Source: BMC Complement Med Ther. 2023 Feb 18;23:54. doi: 10.1186/s12906-023-03842-y (PMC9938629; doi:10.1186/s12906-023-03842-y)
Supplement: Supplementary file 1 — Additional file 1: Supplementary File S1. PRISMA 2020 checklist. Supplementary File S2. AMSTAR 2 checklist. Supplementary File S3. Search strategies for databases. Supplementary File S4. Excluded studies and reasons for exclusion after reading the full text. Supplementary File S5. risk of bias assessment. [file 12906_2023_3842_MOESM1_ESM.docx]

Supplementary Material

**Table of contents**

Supplementary File S1. PRISMA 2020 checklist2

Supplementary File S2. AMSTAR 2 checklist 5

Supplementary File S3. Search strategies for databases 11

Supplementary File S4. Excluded studies and reasons for exclusion after reading the full text 15

Supplementary File S5. risk of bias assessment 19

**Supplementary File S1. PRISMA 2020 checklist**

| **Section and Topic** | **Item #** | **Checklist item** | **Location where item is reported** |
| --- | --- | --- | --- |
| **TITLE** | | |  |
| Title | 1 | Identify the report as a systematic review. | P1 |
| **ABSTRACT** | | |  |
| Abstract | 2 | See the PRISMA 2020 for Abstracts checklist. | P1 |
| **INTRODUCTION** | | |  |
| Rationale | 3 | Describe the rationale for the review in the context of existing knowledge. | P2-3 |
| Objectives | 4 | Provide an explicit statement of the objective(s) or question(s) the review addresses. | P4 |
| **METHODS** | | |  |
| Eligibility criteria | 5 | Specify the inclusion and exclusion criteria for the review and how studies were grouped for the syntheses. | P4-5 |
| Information sources | 6 | Specify all databases, registers, websites, organisations, reference lists and other sources searched or consulted to identify studies. Specify the date when each source was last searched or consulted. | P4 |
| Search strategy | 7 | Present the full search strategies for all databases, registers and websites, including any filters and limits used. | Supplementary  material |
| Selection process | 8 | Specify the methods used to decide whether a study met the inclusion criteria of the review, including how many reviewers screened each record and each report retrieved, whether they worked independently, and if applicable, details of automation tools used in the process. | P5 |
| Data collection process | 9 | Specify the methods used to collect data from reports, including how many reviewers collected data from each report, whether they worked independently, any processes for obtaining or confirming data from study investigators, and if applicable, details of automation tools used in the process. | P5 |
| Data items | 10a | List and define all outcomes for which data were sought. Specify whether all results that were compatible with each outcome domain in each study were sought (e.g. for all measures, time points, analyses), and if not, the methods used to decide which results to collect. | P5 |
|  | 10b | List and define all other variables for which data were sought (e.g. participant and intervention characteristics, funding sources). Describe any assumptions made about any missing or unclear information. | P5 |
| Study risk of bias assessment | 11 | Specify the methods used to assess risk of bias in the included studies, including details of the tool(s) used, how many reviewers assessed each study and whether they worked independently, and if applicable, details of automation tools used in the process. | P5 |
| Effect measures | 12 | Specify for each outcome the effect measure(s) (e.g. risk ratio, mean difference) used in the synthesis or presentation of results. | P5-6 |
| Synthesis methods | 13a | Describe the processes used to decide which studies were eligible for each synthesis (e.g. tabulating the study intervention characteristics and comparing against the planned groups for each synthesis (item #5)). | P5-6 |
|  | 13b | Describe any methods required to prepare the data for presentation or synthesis, such as handling of missing summary statistics, or data conversions. | P5-6 |
|  | 13c | Describe any methods used to tabulate or visually display results of individual studies and syntheses. | P5-6 |
|  | 13d | Describe any methods used to synthesize results and provide a rationale for the choice(s). If meta-analysis was performed, describe the model(s), method(s) to identify the presence and extent of statistical heterogeneity, and software package(s) used. | P5-6 |
|  | 13e | Describe any methods used to explore possible causes of heterogeneity among study results (e.g. subgroup analysis, meta-regression). | P5-6 |
|  | 13f | Describe any sensitivity analyses conducted to assess robustness of the synthesized results. | P5-6 |
| Reporting bias assessment | 14 | Describe any methods used to assess risk of bias due to missing results in a synthesis (arising from reporting biases). | P5-6 |
| Certainty assessment | 15 | Describe any methods used to assess certainty (or confidence) in the body of evidence for an outcome. | P5 |
| **RESULTS** | | |  |
| Study selection | 16a | Describe the results of the search and selection process, from the number of records identified in the search to the number of studies included in the review, ideally using a flow diagram. | P6 |
|  | 16b | Cite studies that might appear to meet the inclusion criteria, but which were excluded, and explain why they were excluded. | Supplementary  material |
| Study characteristics | 17 | Cite each included study and present its characteristics. | P6 |
| Risk of bias in studies | 18 | Present assessments of risk of bias for each included study. | P6 |
| Results of individual studies | 19 | For all outcomes, present, for each study: (a) summary statistics for each group (where appropriate) and (b) an effect estimate and its precision (e.g. confidence/credible interval), ideally using structured tables or plots. | P6-9 |
| Results of syntheses | 20a | For each synthesis, briefly summarise the characteristics and risk of bias among contributing studies. | P6-9 |
|  | 20b | Present results of all statistical syntheses conducted. If meta-analysis was done, present for each the summary estimate and its precision (e.g. confidence/credible interval) and measures of statistical heterogeneity. If comparing groups, describe the direction of the effect. | P6-9 |
|  | 20c | Present results of all investigations of possible causes of heterogeneity among study results. | P6-9 |
|  | 20d | Present results of all sensitivity analyses conducted to assess the robustness of the synthesized results. | P6-9 |
| Reporting biases | 21 | Present assessments of risk of bias due to missing results (arising from reporting biases) for each synthesis assessed. | P9 |
| Certainty of evidence | 22 | Present assessments of certainty (or confidence) in the body of evidence for each outcome assessed. | P9 |
| **DISCUSSION** | | |  |
| Discussion | 23a | Provide a general interpretation of the results in the context of other evidence. | P10-11 |
|  | 23b | Discuss any limitations of the evidence included in the review. | P10-11 |
|  | 23c | Discuss any limitations of the review processes used. | P10-12 |
|  | 23d | Discuss implications of the results for practice, policy, and future research. | P10-12 |
| **OTHER INFORMATION** | | |  |
| Registration and protocol | 24a | Provide registration information for the review, including register name and registration number, or state that the review was not registered. | P3-4 |
|  | 24b | Indicate where the review protocol can be accessed, or state that a protocol was not prepared. | P3-4 |
|  | 24c | Describe and explain any amendments to information provided at registration or in the protocol. | P3-4 |
| Support | 25 | Describe sources of financial or non-financial support for the review, and the role of the funders or sponsors in the review. | P13 |
| Competing interests | 26 | Declare any competing interests of review authors. | P13 |
| Availability of data, code and other materials | 27 | Report which of the following are publicly available and where they can be found: template data collection forms; data extracted from included studies; data used for all analyses; analytic code; any other materials used in the review. | P13 |

*From:*  Page MJ, McKenzie JE, Bossuyt PM, Boutron I, Hoffmann TC, Mulrow CD, et al. The PRISMA 2020 statement: an updated guideline for reporting systematic reviews. BMJ 2021;372:n71. doi: 10.1136/bmj.n71

For more information, visit: <http://www.prisma-statement.org/>

**Supplementary File S2. AMSTAR 2 checklist**

|  |
| --- |
| **1. Did the research questions and inclusion criteria for the review include the components of PICO?** |
| \| For Yes: \| Optional (recommended) \|  \| \| --- \| --- \| --- \| \|  Population \|  Timeframe for follow up \|  Yes   No \| \|  Intervention \|  \| \|  Comparator group \|  \| \|  Outcome \|  \| |
|  |
| \| **2. Did the report of the review contain an explicit statement that the review methods were established prior to the conduct of the review and did the report justify any significant deviations from the protocol?** \| \| --- \| \| \| For Partial Yes: The authors state that they had a written protocol or guide that included ALL the following: \| For Yes: As for partial yes, plus the protocol should be registered and should also have specified: \|  \| \| --- \| --- \| --- \| \|  review question(s) \|  a meta-analysis/synthesis plan, if appropriate, and \|  Yes   Partial Yes   No \| \|  a search strategy \|  a plan for investigating causes of heterogeneity \| \|  inclusion/exclusion criteria \|  a plan for investigating causes of heterogeneity \| \|  a risk of bias assessment \|  \| \| |
|  |
| \| **3. Did the review authors explain their selection of the study designs for inclusion in the review?** \| \| --- \| \| \| For Yes, the review should satisfy ONE of the following: \|  \| \| --- \| --- \| \|  Explanation for including only RCTs \|  Yes   No \| \|  OR Explanation for including only NRSI \| \|  OR Explanation for including both RCTs and NRSI \| \| |
|  |
| \| **4. Did the review authors use a comprehensive literature search strategy?** \| \| --- \| \| \| For Partial Yes (all the following): \| For Yes, should also have (all the following): \|  \| \| --- \| --- \| --- \| \|  searched at least 2 databases (relevant to research question) \|  searched the reference lists / bibliographies of included studies \|  Yes   Partial Yes   No \| \|  provided key word and/or search strategy \|  searched trial/study registries \| \|  justified publication restrictions (e.g. language) \|  included/consulted content experts in the field \| \|  \|  where relevant, searched for grey literature \| \|  \|  conducted search within 24 months of completion of the review \| \| |
|  |
| \| **5. Did the review authors perform study selection in duplicate?** \| \| --- \| \| \| For Yes, either ONE of the following: \|  \| \| --- \| --- \| \|  at least two reviewers independently agreed on selection of eligible studies and achieved consensus on which studies to include \|  Yes   No \| \|  OR two reviewers selected a sample of eligible studies and achieved good agreement (at least 80 percent), with the remainder selected by one reviewer. \| \| |
|  |
| \| **6. Did the review authors perform data extraction in duplicate?** \| \| --- \| \| \| For Yes, either ONE of the following: \|  \| \| --- \| --- \| \|  at least two reviewers achieved consensus on which data to extract from included studies \|  Yes   No \| \|  OR two reviewers extracted data from a sample of eligible studies and achieved good agreement (at least 80 percent), with the remainder extracted by one reviewer. \| \| |
|  |
| \| **7. Did the review authors provide a list of excluded studies and justify the exclusions?** \| \| --- \| \| \| For Partial Yes: \| For Yes, must also have: \|  \| \| --- \| --- \| --- \| \| provided a list of all potentially relevant studies that were read in full-text form but excluded from the review \| Justified the exclusion from the review of each potentially relevant study \|  Yes   Partial Yes   No \| \| |
|  |
| \| **8. Did the review authors describe the included studies in adequate detail?** \| \| --- \| \| \| For Partial Yes (ALL the following): \| For Yes, should also have ALL the following: \|  \| \| --- \| --- \| --- \| \|  described populations \|  described population in detail \|  Yes   Partial Yes   No \| \|  described interventions \|  described intervention in detail (including doses where relevant) \| \|  described comparators \|  described comparator in detail (including doses where relevant) \| \|  described outcomes \|  described study’s setting \| \|  described research designs \|  timeframe for follow-up \| \| |
|  |
| \| **9. Did the review authors use a satisfactory technique for assessing the risk of bias (RoB) in individual studies that were included in the review?** \| \| --- \| \| \| **RCTs** \|  \|  \| \| --- \| --- \| --- \| \| For Partial Yes, must have assessed RoB from \| For Yes, must also have assessed RoB from: \|  \| \|  unconcealed allocation, and \|  allocation sequence that was not truly random, and \|  Yes   Partial Yes   No   Includes only NRSI \| \|  lack of blinding of patients and assessors when assessing outcomes (unnecessary for objective outcomes such as all-cause mortality) \|  selection of the reported result from among multiple measurements or analyses of a specified outcome \| \| **NRSI** \|  \|  \| \| For Partial Yes, must have assessed RoB: \| For Yes, must also have assessed RoB: \|  \| \|  from confounding, and \|  methods used to ascertain exposures and outcomes, and \|  Yes   Partial Yes   No   Includes only RCTs \| \|  from selection bias \|  selection of the reported result from among multiple measurements or analyses of a specified outcome \| \| |
|  |
| \| **10. Did the review authors report on the sources of funding for the studies included in the review?** \| \| --- \| \| \| For Yes \|  \| \| --- \| --- \| \|  Must have reported on the sources of funding for individual studies included in the review. Note: Reporting that the reviewers looked for this information but it was not reported by study authors also qualifies \|  Yes   No \| \| |
|  |
| \| **11. If meta-analysis was performed did the review authors use appropriate methods for statistical combination of results?** \| \| --- \| \| \| **RCTs** \|  \| \| --- \| --- \| \| For Yes: \|  \| \|  The authors justified combining the data in a meta-analysis \|  Yes   No   No meta-analysis conducted \| \|  AND they used an appropriate weighted technique to combine study results and adjusted for heterogeneity if present. \| \|  AND investigated the causes of any heterogeneity \| \|  \|  \| \| **For NRSI** For Yes: \|  \| \|  The authors justified combining the data in a meta-analysis \|  Yes   No   No meta-analysis conducted \| \|  AND they used an appropriate weighted technique to combine study results, adjusting for heterogeneity if present \| \|  AND they statistically combined effect estimates from NRSI that were adjusted for confounding, rather than combining raw data, or justified combining raw data when adjusted effect estimates were not available \| \|  AND they reported separate summary estimates for RCTs and NRSI separately when both were included in the review \| \| |
|  |
| \| **12. If meta-analysis was performed, did the review authors assess the potential impact of RoB in individual studies on the results of the meta-analysis or other evidence synthesis?** \| \| --- \| \| \| For Yes: \|  \| \| --- \| --- \| \|  included only low risk of bias RCTs \|  Yes  No   No meta-analysis conducted \| \|  OR, if the pooled estimate was based on RCTs and/or NRSI at variable RoB, the authors performed analyses to investigate possible impact of RoB on summary estimates of effect. \| \| |
|  |
| \| **13. Did the review authors account for RoB in individual studies when interpreting/ discussing the results of the review?** \| \| --- \| \| \| For Yes: \|  \| \| --- \| --- \| \|  included only low risk of bias RCTs \|  Yes   No \| \|  OR, if RCTs with moderate or high RoB, or NRSI were included the review provided a discussion of the likely impact of RoB on the results \|  \| \| |
|  |
| \| **14. Did the review authors provide a satisfactory explanation for, and discussion of, any heterogeneity observed in the results of the review?** \| \| --- \| \| \| For Yes: \|  \| \| --- \| --- \| \| There was no significant heterogeneity in the results \| Yes   No \| \| OR if heterogeneity was present the authors performed an investigation of sources of any heterogeneity in the results and discussed the impact of this on the results of the review \|  \| \| |
|  |
| \| **15. If they performed quantitative synthesis did the review authors carry out an adequate investigation of publication bias (small study bias) and discuss its likely impact on the results of the review?** \| \| --- \| \| \| For Yes: \|  \| \| --- \| --- \| \| performed graphical or statistical tests for publication bias and discussed the likelihood and magnitude of impact of publication bias \| Yes  No  No meta-analysis conducted \| \| |
|  |
| \| **16. Did the review authors report any potential sources of conflict of interest, including any funding they received for conducting the review?** \| \| --- \| \| \| For Yes: \|  \| \| --- \| --- \| \| The authors reported no competing interests OR \| Yes   No \| \| The authors described their funding sources and how they managed potential conflicts of interest \| \| |
|  |
| To cite this tool: Shea BJ, Reeves BC, Wells G, Thuku M, Hamel C, Moran J, Moher D, Tugwell P, Welch V, Kristjansson E, Henry DA. AMSTAR 2: a critical appraisal tool for systematic reviews that include randomised or non-randomised studies of healthcare interventions, or both. BMJ. 2017 Sep 21;358:j4008. |

**Supplementary File S3. Search strategies for databases.**

CNKI:

A total of 411 articles were retrieved from the CNKI database on April 24, 2022, Beijing time.

Wanfang:

A total of 676 articles were retrieved from the Wanfang database on April 24, 2022, Beijing time.

Cqvip:

A total of 371 articles were retrieved from the Cqvip database on April 24, 2022, Beijing time.

CBM:

A total of 487 articles were retrieved from the CBM database on April 24, 2022, Beijing time.

CHiCTR:

A total of 2 articles were retrieved from the CHiCTR database on April 24, 2022, Beijing time.

We have provided the search strategy in English only in the supplementary file. If you require a Chinese search strategy for any of the five databases listed above, please contact the first author.

PubMed (<https://pubmed.ncbi.nlm.nih.gov/advanced/>):

Search strategies:

A total of 7 articles were retrieved from the PubMed database on April 24, 2022, Beijing time.


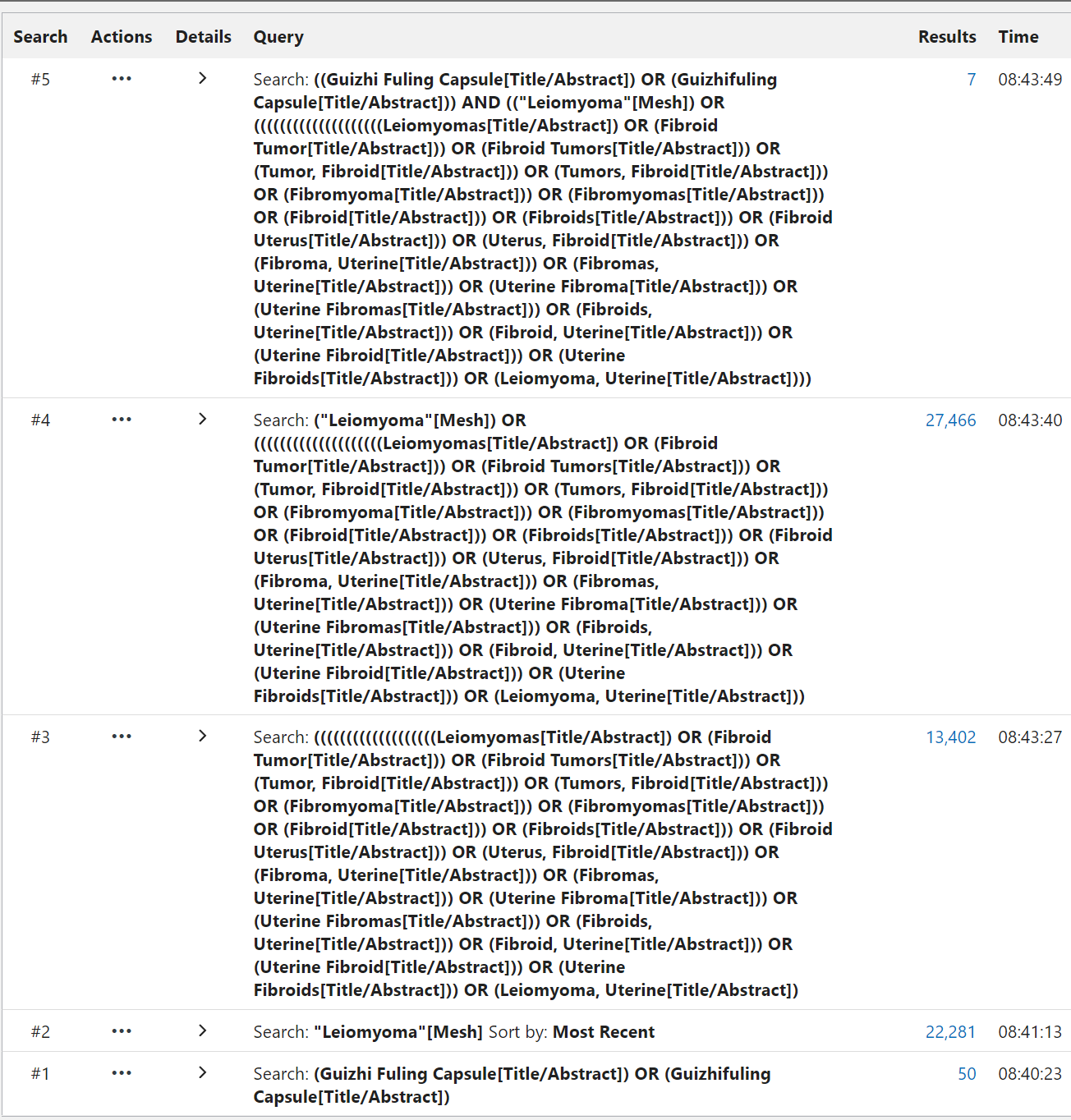


Embase (<https://www.embase.com/>):

Search strategies:

A total of 8 articles were retrieved from Embase database on April 24, 2022, Beijing time.


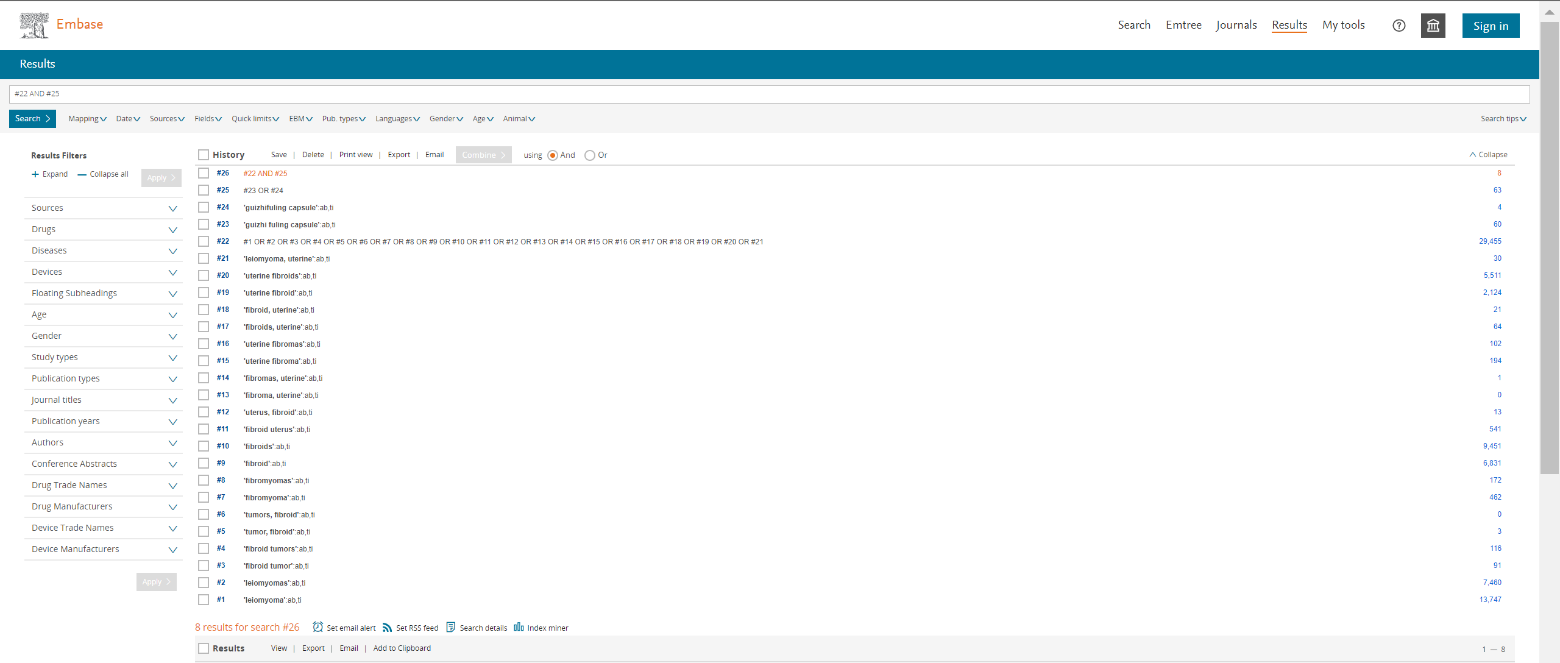


Cochrane Library (<https://www.cochranelibrary.com/>)

Search strategies:

A total of 14 articles were retrieved from the Cochrane Library database on April 24, 2022, Beijing time.


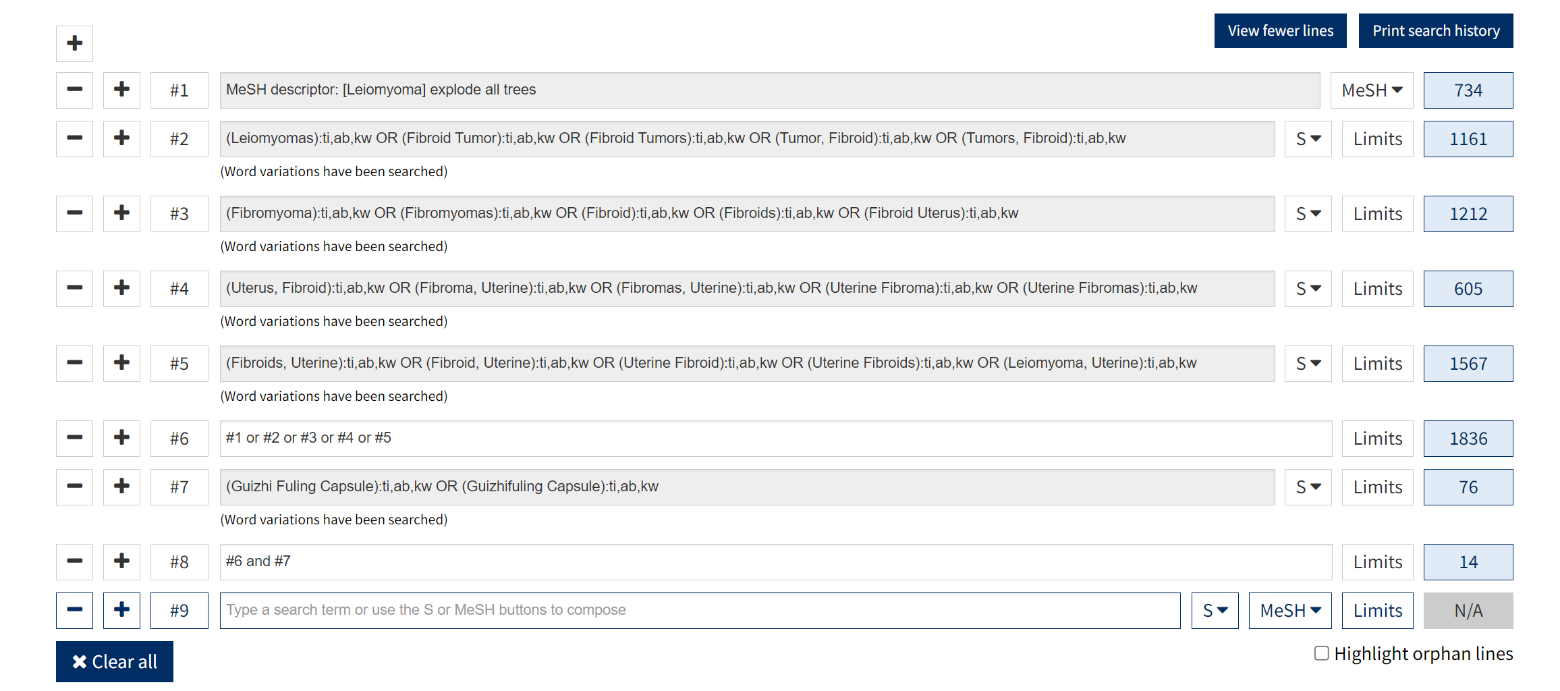


Web of science (<https://www.webofscience.com/wos>):

Search strategies:

A total of 5 articles were retrieved from the Web of science database on April 24, 2022, Beijing time.


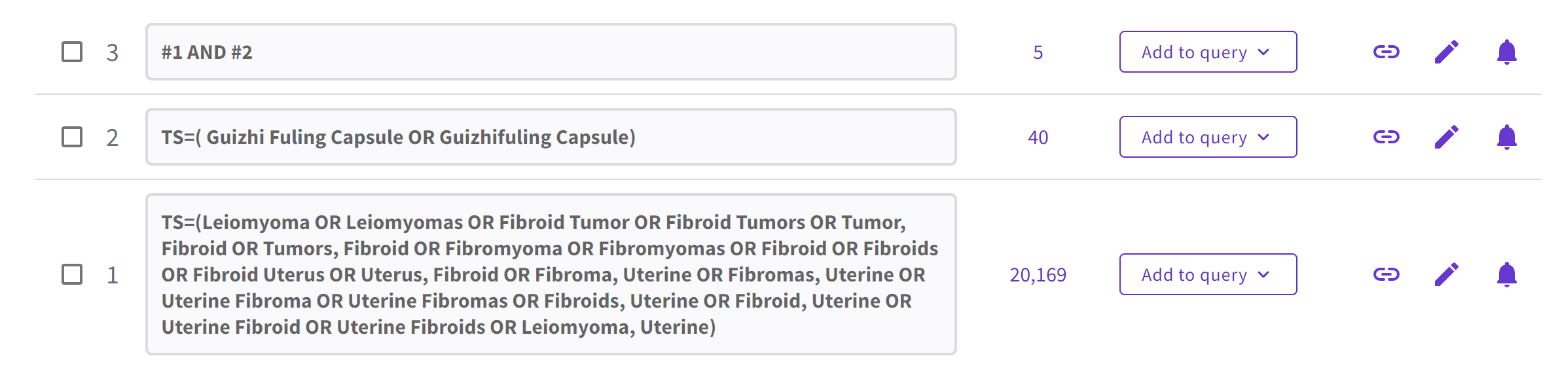


ClinicalTrials.gov (<https://clinicaltrials.gov/>):

Search strategies:

A total of 0 articles were retrieved from the ClinicalTrials.gov database on April 24, 2022, Beijing time.


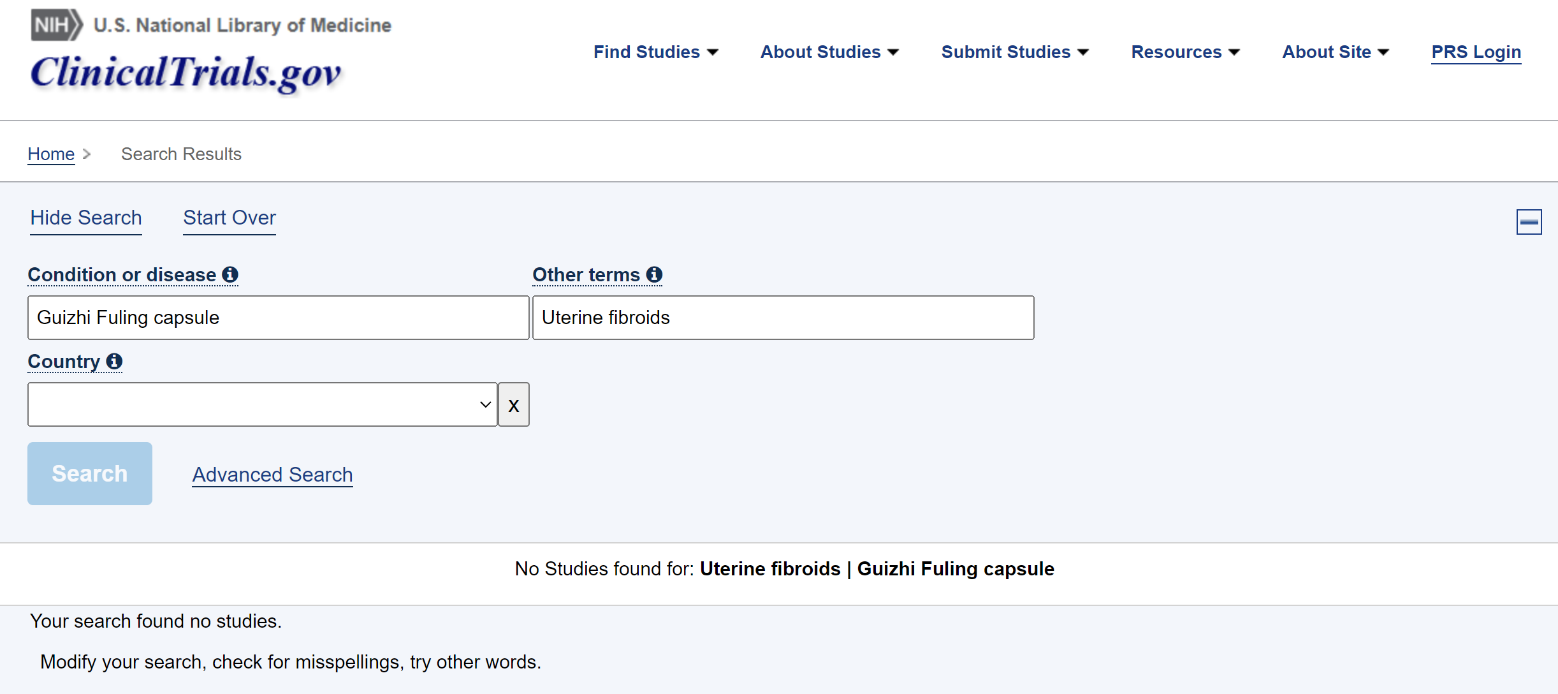


**Supplementary File S4. Excluded studies and reasons for exclusion after reading the full text.**

**Study design:**

The following studies are not RCTs [1-2].

**Intervention:**

The following studies were excluded because they did not use GZFL+MFP [3-22].

**Comparison:**

The following studies were excluded because they did not use MFP [23-29].

**Outcome:**

The following studies were excluded due to insufficient data [30-37].

**References**

1. Gao, C, H. Efficacy observation of 60 cases of uterine fibroids treated with different drugs. Chin Ethnic Folk Med. 2012;21(20),73-74.

2. Wang, R, S., and Zhou, P. Efficacy analysis of Guizhi Fuling capsule combined with low-dose mifepristone in the treatment of uterine fibroids. Chin Prim Med. 2018;25(06),745-748.

3. Huang, M, Q., Yao, R, J., and Wang, W, M. Guizhi Fuling Capsules combined with high-intensity ultrasound focusing in the treatment of 57 cases of premenopausal uterine fibroids. J Clin Chin Med. 2006;(06),612.

4. Zhou, H, W. Clinical observation of Xiaozheng Decoction in the treatment of uterine fibroids. TCM Clin Res. 2017;9(26),105-106.

5. Huang Q, Liu, C, X., Lin, H., and Xu, H. Clinical study of Yang's Xiaozheng Decoction in the treatment of uterine fibroids. TCM Clin Res. 2011;3(14), 25-26.

6. Li, L, H. Clinical comparative study of Guizhi Fuling capsule and Xuefu Zhuyu capsule in adjuvant treatment of uterine fibroids. Chin Med Pharm Clin. 2015;31(06),152-153.

7. Liu, Z, B., and Li, P. Efficacy of Danbie Capsules in the treatment of uterine fibroids. Chin Foreign Med. 2012;31(23),112-113.

8. Song, W, M., and Liu, P. Analysis of the effect of Pingxiao Capsule on the treatment of patients with uterine fibroids and mammary gland hyperplasia. Chin Foreign Women Health Res. 2018;(13),79-80.

9. Fang, R, D., Zhang, X, H., Wang, Q, Y., Lin, Y, Z., Yang, S, P., Rao, W, N., and Jiang, M, Z. Clinical study of Xiaoliuyin iontophoresis in the treatment of uterine fibroids. J Tradit Chin Med. 2001;(12),47-49.

10. Wang, S, L. Self-made Xiaoliu Capsules in the treatment of 50 cases of uterine fibroids. Tradit Chin Med Sci Technol. 2011;18(01),77.

11. Dong, Y, C. Observation on the curative effect of Gongluning in the treatment of uterine fibroids. Chin Community Phys. 2008;(15),136.

12. Yang, X, Q., Jian, L, H., and Yin, J, P. Clinical observation of Danbie capsules. Chin Community Phys. 2011;13(27),176.

13. Wang, S, F. Efficacy observation of compound gossypol acetate tablets combined with Guizhi Fuling capsules in the treatment of uterine fibroids. Chin Prim Med. 2007;14(10),1597-1598.

14. Cao, H, K., Chen W, T., and Zong, F. Clinical observation of high-intensity polymeric ultrasound combined with Guizhi Fuling capsules in the treatment of uterine fibroids. Chin Matern Child Health. 2020;35(20),3895-3897.

15. Miao, X, L. Clinical observation of Xiaoliu Granule in the treatment of 130 cases of uterine fibroids. Yunnan J Tradit Chin Med. 2002;(05),9-10.

16. Xu, L, K. Efficacy analysis of Danqihuaizheng capsule in the treatment of 70 cases of uterine fibroids of Qi stagnation and blood stasis type. Asia Pac Tradit Med. 2015;11(01),126-127.

17. Qi, W, F. Clinical observation of Guizhi Fuling Capsule combined with the method of promoting blood circulation and removing blood stasis in the treatment of uterine fibroids. New Chin Med. 2015;47(09),136-138.

18. Wang, X, H., Sun, J., and Sun, Q. Clinical observation on the treatment of uterine fibroids with the method of nourishing qi and promoting blood circulation and removing blood stasis and Guizhi Fuling capsules combined with mifepristone. New Chin Med. 2015;47(06),158-159.

19. Li, W., Tian, Q., and Hu, X, X. Clinical observation of Gongluning capsule in the treatment of 90 cases of uterine fibroids. New Chin Med. 2009;41(04),30-31.

20. Long, X., Li, X, H., and Liu, Y. Clinical observation of Gongluning in the treatment of uterine fibroids. Western Med. 2011;23(05),860-861+864.

21. Li, L, Z., Li, G, H., and Chen, P. Clinical observation of Sanjie Xiaoliu Decoction in the treatment of uterine fibroids. Tianjin Chin Med. 2003;(05),22-23.

22. Tian, L, J. Clinical observation of Fuliuqing Ⅰ in the treatment of perimenopausal uterine fibroids. Sichuan Tradit Chin Med. 2005;(06),69-70.

23. Jiang, Y, P., Zhang, Q, D., Wang, C, P., Wan, S., Yin, X, G., and Zhou, Y. Clinical observation of Guizhi Fuling Capsules combined with different doses of mifepristone in the treatment of uterine fibroids. Sinopharm. 2019;28(03),45-47.

24. Peng, F, P., Ke, Y., Song, X, L., Chen, H., Zong, Y., Zheng, W, Q., and Chen, M. Clinical efficacy and safety observation of different doses of mifepristone combined with Guizhi Fuling capsules in the treatment of uterine fibroids. Mod drug appl Chin. 2020;14(15),117-119.

25. Mao, M. Clinical study of Guizhi Fuling capsules combined with methyl testosterone in the treatment of uterine fibroids. Chin J Nat Med. 2003;(02),86-87.

26. Xiang, M., and Yang, L, J. Effects of Guizhi Fuling Capsules combined with triptorelin in the treatment of uterine fibroids and their effects on hormone and hemorheology levels. Chin Med Innov. 2019;16(29),19-22.

27. Dong, M. Clinical study of Guizhi Fuling Capsules combined with leuprolide in the treatment of premenopausal uterine fibroids. Chin Med Front. 2011;6(19),69-70.

28. Xu, Q. Effects of Guizhi Fuling Capsules combined with uterine artery embolization on hormone levels and therapeutic effects in middle-aged and elderly patients with uterine fibroids. Chin Matern Child Health. 2016;31(24),5526-5529.

29. Zhuang, T, Y., and Tang, Y, L. Clinical study of Guizhi Fuling Capsules combined with Danazol in the treatment of uterine fibroids. Mod Med Clin. 2019; 34(05),1534-1537.

30. Kuang, Y. To explore the effect of mifepristone in the treatment of uterine fibroids. Health Care Gu. 2021;(19),26.

31. Luo, H, L. Observation on the clinical effect of integrated traditional Chinese and western medicine in the treatment of uterine fibroids. Health Vis. 2013;21(8),180-181.

32. Pan, Q., and Gu, K, B. Observation on the curative effect of Chinese and Western medicine combined with psychological intervention on uterine fibroids. Minkang Chin Med. 2009;21(10),1131-1132,1137.

33. Tao, X, F., Liu, Y, X., Wang, Y, Q., and Wang, Y, Y. Efficacy of Guizhi Fuqin Pills combined with mifepristone in the treatment of uterine fibroids and their effects on serum progesterone receptors, estrogen receptors and vascular endothelial growth factor. Drugs Evaluat Res. 2018;41(4),607-610.

34. Wan, N., Wei, Z, H., and Li, L, G. Analysis of the clinical efficacy of combined traditional Chinese and western medicine in the treatment of uterine fibroids. Health Horiz. 2013;21(18),248-249.

35. Wang, J, Z., and Ma, Y. Clinical Observation of 43 Cases of Uterine Fibroids Treated by Twenty-Nine Tastes Can Dissipate. Chin Health Nutr. 2013;(2),448-448.

36. Xu, H, X. Observation on the efficacy of mifepristone in the treatment of uterine fibroids. Massage Rehabil Med. 2011;2(6),97-97.

37. Yu, Z, H. Observation of curative effect of 69 cases of uterine fibroids treated with integrated traditional Chinese and western medicine. Chin Tradit Med Inform. 2012;4(4),296.

**Supplementary File S5. risk of bias assessment**

| **Unique ID** | A1 | **Study ID** | Cao, 2016 | **Assessor** |  |
| --- | --- | --- | --- | --- | --- |
| **Ref or Label** |  | **Aim** | assignment to intervention (the 'intention-to-treat' effect) | |  |
| **Experimental** | GZFL+MFP | **Comparator** | MFP | **Source** | Journal article(s) with results of the trial |
| **Outcome** | CER | **Results** |  | **Weight** | 1 |
| **Domain** | **Signalling question** |  |  | **Response** | **Comments** |
| **Bias arising from the randomization process** | 1.1 Was the allocation sequence random? | |  | NI | The authors did not describe in detail the generation and allocation concealment of random sequences |
|  | 1.2 Was the allocation sequence concealed until participants were enrolled and assigned to interventions? | | | NI |  |
|  | 1.3 Did baseline differences between intervention groups suggest a problem with the randomization process? | | | N | There is no difference between baseline data |
|  | **Risk of bias judgement** |  |  | **Some concerns** |  |
| **Bias due to deviations from intended interventions** | 2.1.Were participants aware of their assigned intervention during the trial? | | | Y | Does not mention whether blinding is used |
|  | 2.2.Were carers and people delivering the interventions aware of participants' assigned intervention during the trial? | | | Y |  |
|  | 2.3. If Y/PY/NI to 2.1 or 2.2: Were there deviations from the intended intervention that arose because of the experimental context? | | | NI | No relevant information reported |
|  | 2.4 If Y/PY to 2.3: Were these deviations likely to have affected the outcome? | | | NA |  |
|  | 2.5. If Y/PY/NI to 2.4: Were these deviations from intended intervention balanced between groups? | | | NA |  |
|  | 2.6 Was an appropriate analysis used to estimate the effect of assignment to intervention? | | | NI | not enough information |
|  | 2.7 If N/PN/NI to 2.6: Was there potential for a substantial impact (on the result) of the failure to analyse participants in the group to which they were randomized? | | | NI | not enough information |
|  | **Risk of bias judgement** |  |  | **High** |  |
| **Bias due to missing outcome data** | 3.1 Were data for this outcome available for all, or nearly all, participants randomized? | | | Y | The authors report data on all patients involved in randomization |
|  | 3.2 If N/PN/NI to 3.1: Is there evidence that result was not biased by missing outcome data? | | | NA |  |
|  | 3.3 If N/PN to 3.2: Could missingness in the outcome depend on its true value? | | | NA |  |
|  | 3.4 If Y/PY/NI to 3.3: Is it likely that missingness in the outcome depended on its true value? | | | NA |  |
|  | **Risk of bias judgement** |  |  | **Low** |  |
| **Bias in measurement of the outcome** | 4.1 Was the method of measuring the outcome inappropriate? | | | N | method is appropriate |
|  | 4.2 Could measurement or ascertainment of the outcome have differed between intervention groups? | | | N | Outcome measures (data collection) involving the same measurement methods and thresholds, used at comparable time points |
|  | 4.3 Were outcome assessors aware of the intervention received by study participants? | | | NI | not enough information |
|  | 4.4 If Y/PY/NI to 4.3: Could assessment of the outcome have been influenced by knowledge of intervention received? | | | N | Results do not involve judgment |
|  | 4.5 If Y/PY/NI to 4.4: Is it likely that assessment of the outcome was influenced by knowledge of intervention received? | | | NA |  |
|  | **Risk of bias judgement** |  |  | **Low** |  |
| **Bias in selection of the reported result** | 5.1 Were the data that produced this result analysed in accordance with a pre-specified analysis plan that was finalized before unblinded outcome data were available for analysis? | | | NI | No mention of pre-specified analyses or protocols |
|  | 5.2 ... multiple eligible outcome measurements (e.g. scales, definitions, time points) within the outcome domain? | | | NI | not enough information |
|  | 5.3 ... multiple eligible analyses of the data? | |  | NI | not enough information |
|  | **Risk of bias judgement** |  |  | **Some concerns** |  |
| **Overall bias** | **Risk of bias judgement** |  |  | **High** |  |
|  |  |  |  |  |  |
|  |  |  |  |  |  |
| **Unique ID** | A2 | **Study ID** | Chen et al., 2008 | **Assessor** |  |
| **Ref or Label** |  | **Aim** | assignment to intervention (the 'intention-to-treat' effect) | |  |
| **Experimental** | GZFL+MFP | **Comparator** | MFP | **Source** | Journal article(s) with results of the trial |
| **Outcome** | CER | **Results** |  | **Weight** | 1 |
| **Domain** | **Signalling question** |  |  | **Response** | **Comments** |
| **Bias arising from the randomization process** | 1.1 Was the allocation sequence random? | |  | NI | The authors did not describe in detail the generation and allocation concealment of random sequences |
|  | 1.2 Was the allocation sequence concealed until participants were enrolled and assigned to interventions? | | | NI |  |
|  | 1.3 Did baseline differences between intervention groups suggest a problem with the randomization process? | | | N | There is no difference between baseline data |
|  | **Risk of bias judgement** |  |  | **Some concerns** |  |
| **Bias due to deviations from intended interventions** | 2.1.Were participants aware of their assigned intervention during the trial? | | | Y | Does not mention whether blinding is used |
|  | 2.2.Were carers and people delivering the interventions aware of participants' assigned intervention during the trial? | | | Y |  |
|  | 2.3. If Y/PY/NI to 2.1 or 2.2: Were there deviations from the intended intervention that arose because of the experimental context? | | | NI | No relevant information reported |
|  | 2.4 If Y/PY to 2.3: Were these deviations likely to have affected the outcome? | | | NA |  |
|  | 2.5. If Y/PY/NI to 2.4: Were these deviations from intended intervention balanced between groups? | | | NA |  |
|  | 2.6 Was an appropriate analysis used to estimate the effect of assignment to intervention? | | | NI | not enough information |
|  | 2.7 If N/PN/NI to 2.6: Was there potential for a substantial impact (on the result) of the failure to analyse participants in the group to which they were randomized? | | | NI | not enough information |
|  | **Risk of bias judgement** |  |  | **High** |  |
| **Bias due to missing outcome data** | 3.1 Were data for this outcome available for all, or nearly all, participants randomized? | | | Y | The authors report data on all patients involved in randomization |
|  | 3.2 If N/PN/NI to 3.1: Is there evidence that result was not biased by missing outcome data? | | | NA |  |
|  | 3.3 If N/PN to 3.2: Could missingness in the outcome depend on its true value? | | | NA |  |
|  | 3.4 If Y/PY/NI to 3.3: Is it likely that missingness in the outcome depended on its true value? | | | NA |  |
|  | **Risk of bias judgement** |  |  | **Low** |  |
| **Bias in measurement of the outcome** | 4.1 Was the method of measuring the outcome inappropriate? | | | N | method is appropriate |
|  | 4.2 Could measurement or ascertainment of the outcome have differed between intervention groups? | | | N | Outcome measures (data collection) involving the same measurement methods and thresholds, used at comparable time points |
|  | 4.3 Were outcome assessors aware of the intervention received by study participants? | | | NI | not enough information |
|  | 4.4 If Y/PY/NI to 4.3: Could assessment of the outcome have been influenced by knowledge of intervention received? | | | N | Results do not involve judgment |
|  | 4.5 If Y/PY/NI to 4.4: Is it likely that assessment of the outcome was influenced by knowledge of intervention received? | | | NA |  |
|  | **Risk of bias judgement** |  |  | **Low** |  |
| **Bias in selection of the reported result** | 5.1 Were the data that produced this result analysed in accordance with a pre-specified analysis plan that was finalized before unblinded outcome data were available for analysis? | | | NI | No mention of pre-specified analyses or protocols |
|  | 5.2 ... multiple eligible outcome measurements (e.g. scales, definitions, time points) within the outcome domain? | | | NI | not enough information |
|  | 5.3 ... multiple eligible analyses of the data? | |  | NI | not enough information |
|  | **Risk of bias judgement** |  |  | **Some concerns** |  |
| **Overall bias** | **Risk of bias judgement** |  |  | **High** |  |
|  |  |  |  |  |  |
|  |  |  |  |  |  |
| **Unique ID** | A3 | **Study ID** | Deng and Li, 2010 | **Assessor** |  |
| **Ref or Label** |  | **Aim** | assignment to intervention (the 'intention-to-treat' effect) | |  |
| **Experimental** | GZFL+MFP | **Comparator** | MFP | **Source** | Journal article(s) with results of the trial |
| **Outcome** | CER | **Results** |  | **Weight** | 1 |
| **Domain** | **Signalling question** |  |  | **Response** | **Comments** |
| **Bias arising from the randomization process** | 1.1 Was the allocation sequence random? | |  | NI | The authors did not describe in detail the generation and allocation concealment of random sequences |
|  | 1.2 Was the allocation sequence concealed until participants were enrolled and assigned to interventions? | | | NI |  |
|  | 1.3 Did baseline differences between intervention groups suggest a problem with the randomization process? | | | N | There is no difference between baseline data |
|  | **Risk of bias judgement** |  |  | **Some concerns** |  |
| **Bias due to deviations from intended interventions** | 2.1.Were participants aware of their assigned intervention during the trial? | | | Y | Does not mention whether blinding is used |
|  | 2.2.Were carers and people delivering the interventions aware of participants' assigned intervention during the trial? | | | Y |  |
|  | 2.3. If Y/PY/NI to 2.1 or 2.2: Were there deviations from the intended intervention that arose because of the experimental context? | | | NI | No relevant information reported |
|  | 2.4 If Y/PY to 2.3: Were these deviations likely to have affected the outcome? | | | NA |  |
|  | 2.5. If Y/PY/NI to 2.4: Were these deviations from intended intervention balanced between groups? | | | NA |  |
|  | 2.6 Was an appropriate analysis used to estimate the effect of assignment to intervention? | | | NI | not enough information |
|  | 2.7 If N/PN/NI to 2.6: Was there potential for a substantial impact (on the result) of the failure to analyse participants in the group to which they were randomized? | | | NI | not enough information |
|  | **Risk of bias judgement** |  |  | **High** |  |
| **Bias due to missing outcome data** | 3.1 Were data for this outcome available for all, or nearly all, participants randomized? | | | Y | The authors report data on all patients involved in randomization |
|  | 3.2 If N/PN/NI to 3.1: Is there evidence that result was not biased by missing outcome data? | | | NA |  |
|  | 3.3 If N/PN to 3.2: Could missingness in the outcome depend on its true value? | | | NA |  |
|  | 3.4 If Y/PY/NI to 3.3: Is it likely that missingness in the outcome depended on its true value? | | | NA |  |
|  | **Risk of bias judgement** |  |  | **Low** |  |
| **Bias in measurement of the outcome** | 4.1 Was the method of measuring the outcome inappropriate? | | | N | method is appropriate |
|  | 4.2 Could measurement or ascertainment of the outcome have differed between intervention groups? | | | N | Outcome measures (data collection) involving the same measurement methods and thresholds, used at comparable time points |
|  | 4.3 Were outcome assessors aware of the intervention received by study participants? | | | NI | not enough information |
|  | 4.4 If Y/PY/NI to 4.3: Could assessment of the outcome have been influenced by knowledge of intervention received? | | | N | Results do not involve judgment |
|  | 4.5 If Y/PY/NI to 4.4: Is it likely that assessment of the outcome was influenced by knowledge of intervention received? | | | NA |  |
|  | **Risk of bias judgement** |  |  | **Low** |  |
| **Bias in selection of the reported result** | 5.1 Were the data that produced this result analysed in accordance with a pre-specified analysis plan that was finalized before unblinded outcome data were available for analysis? | | | NI | No mention of pre-specified analyses or protocols |
|  | 5.2 ... multiple eligible outcome measurements (e.g. scales, definitions, time points) within the outcome domain? | | | NI | not enough information |
|  | 5.3 ... multiple eligible analyses of the data? | |  | NI | not enough information |
|  | **Risk of bias judgement** |  |  | **Some concerns** |  |
| **Overall bias** | **Risk of bias judgement** |  |  | **High** |  |
|  |  |  |  |  |  |
|  |  |  |  |  |  |
| **Unique ID** | A4 | **Study ID** | Fei, 2017 | **Assessor** |  |
| **Ref or Label** |  | **Aim** | assignment to intervention (the 'intention-to-treat' effect) | |  |
| **Experimental** | GZFL+MFP | **Comparator** | MFP | **Source** | Journal article(s) with results of the trial |
| **Outcome** | CER | **Results** |  | **Weight** | 1 |
| **Domain** | **Signalling question** |  |  | **Response** | **Comments** |
| **Bias arising from the randomization process** | 1.1 Was the allocation sequence random? | |  | Y | The author uses the random number table method |
|  | 1.2 Was the allocation sequence concealed until participants were enrolled and assigned to interventions? | | | Y |  |
|  | 1.3 Did baseline differences between intervention groups suggest a problem with the randomization process? | | | N | There is no difference between baseline data |
|  | **Risk of bias judgement** |  |  | **Low** |  |
| **Bias due to deviations from intended interventions** | 2.1.Were participants aware of their assigned intervention during the trial? | | | Y | Does not mention whether blinding is used |
|  | 2.2.Were carers and people delivering the interventions aware of participants' assigned intervention during the trial? | | | Y |  |
|  | 2.3. If Y/PY/NI to 2.1 or 2.2: Were there deviations from the intended intervention that arose because of the experimental context? | | | NI | No relevant information reported |
|  | 2.4 If Y/PY to 2.3: Were these deviations likely to have affected the outcome? | | | NA |  |
|  | 2.5. If Y/PY/NI to 2.4: Were these deviations from intended intervention balanced between groups? | | | NA |  |
|  | 2.6 Was an appropriate analysis used to estimate the effect of assignment to intervention? | | | NI | not enough information |
|  | 2.7 If N/PN/NI to 2.6: Was there potential for a substantial impact (on the result) of the failure to analyse participants in the group to which they were randomized? | | | NI | not enough information |
|  | **Risk of bias judgement** |  |  | **High** |  |
| **Bias due to missing outcome data** | 3.1 Were data for this outcome available for all, or nearly all, participants randomized? | | | Y | The authors report data on all patients involved in randomization |
|  | 3.2 If N/PN/NI to 3.1: Is there evidence that result was not biased by missing outcome data? | | | NA |  |
|  | 3.3 If N/PN to 3.2: Could missingness in the outcome depend on its true value? | | | NA |  |
|  | 3.4 If Y/PY/NI to 3.3: Is it likely that missingness in the outcome depended on its true value? | | | NA |  |
|  | **Risk of bias judgement** |  |  | **Low** |  |
| **Bias in measurement of the outcome** | 4.1 Was the method of measuring the outcome inappropriate? | | | N | method is appropriate |
|  | 4.2 Could measurement or ascertainment of the outcome have differed between intervention groups? | | | N | Outcome measures (data collection) involving the same measurement methods and thresholds, used at comparable time points |
|  | 4.3 Were outcome assessors aware of the intervention received by study participants? | | | NI | not enough information |
|  | 4.4 If Y/PY/NI to 4.3: Could assessment of the outcome have been influenced by knowledge of intervention received? | | | N | Results do not involve judgment |
|  | 4.5 If Y/PY/NI to 4.4: Is it likely that assessment of the outcome was influenced by knowledge of intervention received? | | | NA |  |
|  | **Risk of bias judgement** |  |  | **Low** |  |
| **Bias in selection of the reported result** | 5.1 Were the data that produced this result analysed in accordance with a pre-specified analysis plan that was finalized before unblinded outcome data were available for analysis? | | | NI | No mention of pre-specified analyses or protocols |
|  | 5.2 ... multiple eligible outcome measurements (e.g. scales, definitions, time points) within the outcome domain? | | | NI | not enough information |
|  | 5.3 ... multiple eligible analyses of the data? | |  | NI | not enough information |
|  | **Risk of bias judgement** |  |  | **Some concerns** |  |
| **Overall bias** | **Risk of bias judgement** |  |  | **High** |  |
|  |  |  |  |  |  |
|  |  |  |  |  |  |
| **Unique ID** | A5 | **Study ID** | Gu and Hu, 2011 | **Assessor** |  |
| **Ref or Label** |  | **Aim** | assignment to intervention (the 'intention-to-treat' effect) | |  |
| **Experimental** | GZFL+MFP | **Comparator** | MFP | **Source** | Journal article(s) with results of the trial |
| **Outcome** | CER | **Results** |  | **Weight** | 1 |
| **Domain** | **Signalling question** |  |  | **Response** | **Comments** |
| **Bias arising from the randomization process** | 1.1 Was the allocation sequence random? | |  | Y | The author uses the random number table method |
|  | 1.2 Was the allocation sequence concealed until participants were enrolled and assigned to interventions? | | | Y |  |
|  | 1.3 Did baseline differences between intervention groups suggest a problem with the randomization process? | | | N | There is no difference between baseline data |
|  | **Risk of bias judgement** |  |  | **Low** |  |
| **Bias due to deviations from intended interventions** | 2.1.Were participants aware of their assigned intervention during the trial? | | | Y | Does not mention whether blinding is used |
|  | 2.2.Were carers and people delivering the interventions aware of participants' assigned intervention during the trial? | | | Y |  |
|  | 2.3. If Y/PY/NI to 2.1 or 2.2: Were there deviations from the intended intervention that arose because of the experimental context? | | | NI | No relevant information reported |
|  | 2.4 If Y/PY to 2.3: Were these deviations likely to have affected the outcome? | | | NA |  |
|  | 2.5. If Y/PY/NI to 2.4: Were these deviations from intended intervention balanced between groups? | | | NA |  |
|  | 2.6 Was an appropriate analysis used to estimate the effect of assignment to intervention? | | | NI | not enough information |
|  | 2.7 If N/PN/NI to 2.6: Was there potential for a substantial impact (on the result) of the failure to analyse participants in the group to which they were randomized? | | | NI | not enough information |
|  | **Risk of bias judgement** |  |  | **High** |  |
| **Bias due to missing outcome data** | 3.1 Were data for this outcome available for all, or nearly all, participants randomized? | | | Y | The authors report data on all patients involved in randomization |
|  | 3.2 If N/PN/NI to 3.1: Is there evidence that result was not biased by missing outcome data? | | | NA |  |
|  | 3.3 If N/PN to 3.2: Could missingness in the outcome depend on its true value? | | | NA |  |
|  | 3.4 If Y/PY/NI to 3.3: Is it likely that missingness in the outcome depended on its true value? | | | NA |  |
|  | **Risk of bias judgement** |  |  | **Low** |  |
| **Bias in measurement of the outcome** | 4.1 Was the method of measuring the outcome inappropriate? | | | N | method is appropriate |
|  | 4.2 Could measurement or ascertainment of the outcome have differed between intervention groups? | | | N | Outcome measures (data collection) involving the same measurement methods and thresholds, used at comparable time points |
|  | 4.3 Were outcome assessors aware of the intervention received by study participants? | | | NI | not enough information |
|  | 4.4 If Y/PY/NI to 4.3: Could assessment of the outcome have been influenced by knowledge of intervention received? | | | N | Results do not involve judgment |
|  | 4.5 If Y/PY/NI to 4.4: Is it likely that assessment of the outcome was influenced by knowledge of intervention received? | | | NA |  |
|  | **Risk of bias judgement** |  |  | **Low** |  |
| **Bias in selection of the reported result** | 5.1 Were the data that produced this result analysed in accordance with a pre-specified analysis plan that was finalized before unblinded outcome data were available for analysis? | | | NI | No mention of pre-specified analyses or protocols |
|  | 5.2 ... multiple eligible outcome measurements (e.g. scales, definitions, time points) within the outcome domain? | | | NI | not enough information |
|  | 5.3 ... multiple eligible analyses of the data? | |  | NI | not enough information |
|  | **Risk of bias judgement** |  |  | **Some concerns** |  |
| **Overall bias** | **Risk of bias judgement** |  |  | **High** |  |
|  |  |  |  |  |  |
|  |  |  |  |  |  |
| **Unique ID** | A6 | **Study ID** | Hu, 2013 | **Assessor** |  |
| **Ref or Label** |  | **Aim** | assignment to intervention (the 'intention-to-treat' effect) | |  |
| **Experimental** | GZFL+MFP | **Comparator** | MFP | **Source** | Journal article(s) with results of the trial |
| **Outcome** | CER | **Results** |  | **Weight** | 1 |
| **Domain** | **Signalling question** |  |  | **Response** | **Comments** |
| **Bias arising from the randomization process** | 1.1 Was the allocation sequence random? | |  | NI | The authors did not describe in detail the generation and allocation concealment of random sequences |
|  | 1.2 Was the allocation sequence concealed until participants were enrolled and assigned to interventions? | | | NI |  |
|  | 1.3 Did baseline differences between intervention groups suggest a problem with the randomization process? | | | N | There is no difference between baseline data |
|  | **Risk of bias judgement** |  |  | **Some concerns** |  |
| **Bias due to deviations from intended interventions** | 2.1.Were participants aware of their assigned intervention during the trial? | | | Y | Does not mention whether blinding is used |
|  | 2.2.Were carers and people delivering the interventions aware of participants' assigned intervention during the trial? | | | Y |  |
|  | 2.3. If Y/PY/NI to 2.1 or 2.2: Were there deviations from the intended intervention that arose because of the experimental context? | | | NI | No relevant information reported |
|  | 2.4 If Y/PY to 2.3: Were these deviations likely to have affected the outcome? | | | NA |  |
|  | 2.5. If Y/PY/NI to 2.4: Were these deviations from intended intervention balanced between groups? | | | NA |  |
|  | 2.6 Was an appropriate analysis used to estimate the effect of assignment to intervention? | | | NI | not enough information |
|  | 2.7 If N/PN/NI to 2.6: Was there potential for a substantial impact (on the result) of the failure to analyse participants in the group to which they were randomized? | | | NI | not enough information |
|  | **Risk of bias judgement** |  |  | **High** |  |
| **Bias due to missing outcome data** | 3.1 Were data for this outcome available for all, or nearly all, participants randomized? | | | Y | The authors report data on all patients involved in randomization |
|  | 3.2 If N/PN/NI to 3.1: Is there evidence that result was not biased by missing outcome data? | | | NA |  |
|  | 3.3 If N/PN to 3.2: Could missingness in the outcome depend on its true value? | | | NA |  |
|  | 3.4 If Y/PY/NI to 3.3: Is it likely that missingness in the outcome depended on its true value? | | | NA |  |
|  | **Risk of bias judgement** |  |  | **Low** |  |
| **Bias in measurement of the outcome** | 4.1 Was the method of measuring the outcome inappropriate? | | | N | method is appropriate |
|  | 4.2 Could measurement or ascertainment of the outcome have differed between intervention groups? | | | N | Outcome measures (data collection) involving the same measurement methods and thresholds, used at comparable time points |
|  | 4.3 Were outcome assessors aware of the intervention received by study participants? | | | NI | not enough information |
|  | 4.4 If Y/PY/NI to 4.3: Could assessment of the outcome have been influenced by knowledge of intervention received? | | | N | Results do not involve judgment |
|  | 4.5 If Y/PY/NI to 4.4: Is it likely that assessment of the outcome was influenced by knowledge of intervention received? | | | NA |  |
|  | **Risk of bias judgement** |  |  | **Low** |  |
| **Bias in selection of the reported result** | 5.1 Were the data that produced this result analysed in accordance with a pre-specified analysis plan that was finalized before unblinded outcome data were available for analysis? | | | NI | No mention of pre-specified analyses or protocols |
|  | 5.2 ... multiple eligible outcome measurements (e.g. scales, definitions, time points) within the outcome domain? | | | NI | not enough information |
|  | 5.3 ... multiple eligible analyses of the data? | |  | NI | not enough information |
|  | **Risk of bias judgement** |  |  | **Some concerns** |  |
| **Overall bias** | **Risk of bias judgement** |  |  | **High** |  |
|  |  |  |  |  |  |
|  |  |  |  |  |  |
| **Unique ID** | A7 | **Study ID** | Li and Gao, 2015 | **Assessor** |  |
| **Ref or Label** |  | **Aim** | assignment to intervention (the 'intention-to-treat' effect) | |  |
| **Experimental** | GZFL+MFP | **Comparator** | MFP | **Source** | Journal article(s) with results of the trial |
| **Outcome** | CER | **Results** |  | **Weight** | 1 |
| **Domain** | **Signalling question** |  |  | **Response** | **Comments** |
| **Bias arising from the randomization process** | 1.1 Was the allocation sequence random? | |  | Y | The author uses the random number table method |
|  | 1.2 Was the allocation sequence concealed until participants were enrolled and assigned to interventions? | | | Y |  |
|  | 1.3 Did baseline differences between intervention groups suggest a problem with the randomization process? | | | N | There is no difference between baseline data |
|  | **Risk of bias judgement** |  |  | **Low** |  |
| **Bias due to deviations from intended interventions** | 2.1.Were participants aware of their assigned intervention during the trial? | | | Y | Does not mention whether blinding is used |
|  | 2.2.Were carers and people delivering the interventions aware of participants' assigned intervention during the trial? | | | Y |  |
|  | 2.3. If Y/PY/NI to 2.1 or 2.2: Were there deviations from the intended intervention that arose because of the experimental context? | | | NI | No relevant information reported |
|  | 2.4 If Y/PY to 2.3: Were these deviations likely to have affected the outcome? | | | NA |  |
|  | 2.5. If Y/PY/NI to 2.4: Were these deviations from intended intervention balanced between groups? | | | NA |  |
|  | 2.6 Was an appropriate analysis used to estimate the effect of assignment to intervention? | | | NI | not enough information |
|  | 2.7 If N/PN/NI to 2.6: Was there potential for a substantial impact (on the result) of the failure to analyse participants in the group to which they were randomized? | | | NI | not enough information |
|  | **Risk of bias judgement** |  |  | **High** |  |
| **Bias due to missing outcome data** | 3.1 Were data for this outcome available for all, or nearly all, participants randomized? | | | Y | The authors report data on all patients involved in randomization |
|  | 3.2 If N/PN/NI to 3.1: Is there evidence that result was not biased by missing outcome data? | | | NA |  |
|  | 3.3 If N/PN to 3.2: Could missingness in the outcome depend on its true value? | | | NA |  |
|  | 3.4 If Y/PY/NI to 3.3: Is it likely that missingness in the outcome depended on its true value? | | | NA |  |
|  | **Risk of bias judgement** |  |  | **Low** |  |
| **Bias in measurement of the outcome** | 4.1 Was the method of measuring the outcome inappropriate? | | | N | method is appropriate |
|  | 4.2 Could measurement or ascertainment of the outcome have differed between intervention groups? | | | N | Outcome measures (data collection) involving the same measurement methods and thresholds, used at comparable time points |
|  | 4.3 Were outcome assessors aware of the intervention received by study participants? | | | NI | not enough information |
|  | 4.4 If Y/PY/NI to 4.3: Could assessment of the outcome have been influenced by knowledge of intervention received? | | | N | Results do not involve judgment |
|  | 4.5 If Y/PY/NI to 4.4: Is it likely that assessment of the outcome was influenced by knowledge of intervention received? | | | NA |  |
|  | **Risk of bias judgement** |  |  | **Low** |  |
| **Bias in selection of the reported result** | 5.1 Were the data that produced this result analysed in accordance with a pre-specified analysis plan that was finalized before unblinded outcome data were available for analysis? | | | NI | No mention of pre-specified analyses or protocols |
|  | 5.2 ... multiple eligible outcome measurements (e.g. scales, definitions, time points) within the outcome domain? | | | NI | not enough information |
|  | 5.3 ... multiple eligible analyses of the data? | |  | NI | not enough information |
|  | **Risk of bias judgement** |  |  | **Some concerns** |  |
| **Overall bias** | **Risk of bias judgement** |  |  | **High** |  |
|  |  |  |  |  |  |
|  |  |  |  |  |  |
| **Unique ID** | A8 | **Study ID** | Li, 2017 | **Assessor** |  |
| **Ref or Label** |  | **Aim** | assignment to intervention (the 'intention-to-treat' effect) | |  |
| **Experimental** | GZFL+MFP | **Comparator** | MFP | **Source** | Journal article(s) with results of the trial |
| **Outcome** | CER | **Results** |  | **Weight** | 1 |
| **Domain** | **Signalling question** |  |  | **Response** | **Comments** |
| **Bias arising from the randomization process** | 1.1 Was the allocation sequence random? | |  | Y | The author uses the random number table method |
|  | 1.2 Was the allocation sequence concealed until participants were enrolled and assigned to interventions? | | | Y |  |
|  | 1.3 Did baseline differences between intervention groups suggest a problem with the randomization process? | | | N | There is no difference between baseline data |
|  | **Risk of bias judgement** |  |  | **Low** |  |
| **Bias due to deviations from intended interventions** | 2.1.Were participants aware of their assigned intervention during the trial? | | | Y | Does not mention whether blinding is used |
|  | 2.2.Were carers and people delivering the interventions aware of participants' assigned intervention during the trial? | | | Y |  |
|  | 2.3. If Y/PY/NI to 2.1 or 2.2: Were there deviations from the intended intervention that arose because of the experimental context? | | | NI | No relevant information reported |
|  | 2.4 If Y/PY to 2.3: Were these deviations likely to have affected the outcome? | | | NA |  |
|  | 2.5. If Y/PY/NI to 2.4: Were these deviations from intended intervention balanced between groups? | | | NA |  |
|  | 2.6 Was an appropriate analysis used to estimate the effect of assignment to intervention? | | | NI | not enough information |
|  | 2.7 If N/PN/NI to 2.6: Was there potential for a substantial impact (on the result) of the failure to analyse participants in the group to which they were randomized? | | | NI | not enough information |
|  | **Risk of bias judgement** |  |  | **High** |  |
| **Bias due to missing outcome data** | 3.1 Were data for this outcome available for all, or nearly all, participants randomized? | | | Y | The authors report data on all patients involved in randomization |
|  | 3.2 If N/PN/NI to 3.1: Is there evidence that result was not biased by missing outcome data? | | | NA |  |
|  | 3.3 If N/PN to 3.2: Could missingness in the outcome depend on its true value? | | | NA |  |
|  | 3.4 If Y/PY/NI to 3.3: Is it likely that missingness in the outcome depended on its true value? | | | NA |  |
|  | **Risk of bias judgement** |  |  | **Low** |  |
| **Bias in measurement of the outcome** | 4.1 Was the method of measuring the outcome inappropriate? | | | N | method is appropriate |
|  | 4.2 Could measurement or ascertainment of the outcome have differed between intervention groups? | | | N | Outcome measures (data collection) involving the same measurement methods and thresholds, used at comparable time points |
|  | 4.3 Were outcome assessors aware of the intervention received by study participants? | | | NI | not enough information |
|  | 4.4 If Y/PY/NI to 4.3: Could assessment of the outcome have been influenced by knowledge of intervention received? | | | N | Results do not involve judgment |
|  | 4.5 If Y/PY/NI to 4.4: Is it likely that assessment of the outcome was influenced by knowledge of intervention received? | | | NA |  |
|  | **Risk of bias judgement** |  |  | **Low** |  |
| **Bias in selection of the reported result** | 5.1 Were the data that produced this result analysed in accordance with a pre-specified analysis plan that was finalized before unblinded outcome data were available for analysis? | | | NI | No mention of pre-specified analyses or protocols |
|  | 5.2 ... multiple eligible outcome measurements (e.g. scales, definitions, time points) within the outcome domain? | | | NI | not enough information |
|  | 5.3 ... multiple eligible analyses of the data? | |  | NI | not enough information |
|  | **Risk of bias judgement** |  |  | **Some concerns** |  |
| **Overall bias** | **Risk of bias judgement** |  |  | **High** |  |
|  |  |  |  |  |  |
|  |  |  |  |  |  |
| **Unique ID** | A9 | **Study ID** | Liang, 2017 | **Assessor** |  |
| **Ref or Label** |  | **Aim** | assignment to intervention (the 'intention-to-treat' effect) | |  |
| **Experimental** | GZFL+MFP | **Comparator** | MFP | **Source** | Journal article(s) with results of the trial |
| **Outcome** | CER | **Results** |  | **Weight** | 1 |
| **Domain** | **Signalling question** |  |  | **Response** | **Comments** |
| **Bias arising from the randomization process** | 1.1 Was the allocation sequence random? | |  | NI | The authors did not describe in detail the generation and allocation concealment of random sequences |
|  | 1.2 Was the allocation sequence concealed until participants were enrolled and assigned to interventions? | | | NI |  |
|  | 1.3 Did baseline differences between intervention groups suggest a problem with the randomization process? | | | N | There is no difference between baseline data |
|  | **Risk of bias judgement** |  |  | **Some concerns** |  |
| **Bias due to deviations from intended interventions** | 2.1.Were participants aware of their assigned intervention during the trial? | | | Y | Does not mention whether blinding is used |
|  | 2.2.Were carers and people delivering the interventions aware of participants' assigned intervention during the trial? | | | Y |  |
|  | 2.3. If Y/PY/NI to 2.1 or 2.2: Were there deviations from the intended intervention that arose because of the experimental context? | | | NI | No relevant information reported |
|  | 2.4 If Y/PY to 2.3: Were these deviations likely to have affected the outcome? | | | NA |  |
|  | 2.5. If Y/PY/NI to 2.4: Were these deviations from intended intervention balanced between groups? | | | NA |  |
|  | 2.6 Was an appropriate analysis used to estimate the effect of assignment to intervention? | | | NI | not enough information |
|  | 2.7 If N/PN/NI to 2.6: Was there potential for a substantial impact (on the result) of the failure to analyse participants in the group to which they were randomized? | | | NI | not enough information |
|  | **Risk of bias judgement** |  |  | **High** |  |
| **Bias due to missing outcome data** | 3.1 Were data for this outcome available for all, or nearly all, participants randomized? | | | Y | The authors report data on all patients involved in randomization |
|  | 3.2 If N/PN/NI to 3.1: Is there evidence that result was not biased by missing outcome data? | | | NA |  |
|  | 3.3 If N/PN to 3.2: Could missingness in the outcome depend on its true value? | | | NA |  |
|  | 3.4 If Y/PY/NI to 3.3: Is it likely that missingness in the outcome depended on its true value? | | | NA |  |
|  | **Risk of bias judgement** |  |  | **Low** |  |
| **Bias in measurement of the outcome** | 4.1 Was the method of measuring the outcome inappropriate? | | | N | method is appropriate |
|  | 4.2 Could measurement or ascertainment of the outcome have differed between intervention groups? | | | N | Outcome measures (data collection) involving the same measurement methods and thresholds, used at comparable time points |
|  | 4.3 Were outcome assessors aware of the intervention received by study participants? | | | NI | not enough information |
|  | 4.4 If Y/PY/NI to 4.3: Could assessment of the outcome have been influenced by knowledge of intervention received? | | | N | Results do not involve judgment |
|  | 4.5 If Y/PY/NI to 4.4: Is it likely that assessment of the outcome was influenced by knowledge of intervention received? | | | NA |  |
|  | **Risk of bias judgement** |  |  | **Low** |  |
| **Bias in selection of the reported result** | 5.1 Were the data that produced this result analysed in accordance with a pre-specified analysis plan that was finalized before unblinded outcome data were available for analysis? | | | NI | No mention of pre-specified analyses or protocols |
|  | 5.2 ... multiple eligible outcome measurements (e.g. scales, definitions, time points) within the outcome domain? | | | NI | not enough information |
|  | 5.3 ... multiple eligible analyses of the data? | |  | NI | not enough information |
|  | **Risk of bias judgement** |  |  | **Some concerns** |  |
| **Overall bias** | **Risk of bias judgement** |  |  | **High** |  |
|  |  |  |  |  |  |
|  |  |  |  |  |  |
| **Unique ID** | A10 | **Study ID** | Lin, 2019 | **Assessor** |  |
| **Ref or Label** |  | **Aim** | assignment to intervention (the 'intention-to-treat' effect) | |  |
| **Experimental** | GZFL+MFP | **Comparator** | MFP | **Source** | Journal article(s) with results of the trial |
| **Outcome** | CER | **Results** |  | **Weight** | 1 |
| **Domain** | **Signalling question** |  |  | **Response** | **Comments** |
| **Bias arising from the randomization process** | 1.1 Was the allocation sequence random? | |  | NI | The authors did not describe in detail the generation and allocation concealment of random sequences |
|  | 1.2 Was the allocation sequence concealed until participants were enrolled and assigned to interventions? | | | NI |  |
|  | 1.3 Did baseline differences between intervention groups suggest a problem with the randomization process? | | | N | There is no difference between baseline data |
|  | **Risk of bias judgement** |  |  | **Some concerns** |  |
| **Bias due to deviations from intended interventions** | 2.1.Were participants aware of their assigned intervention during the trial? | | | Y | Does not mention whether blinding is used |
|  | 2.2.Were carers and people delivering the interventions aware of participants' assigned intervention during the trial? | | | Y |  |
|  | 2.3. If Y/PY/NI to 2.1 or 2.2: Were there deviations from the intended intervention that arose because of the experimental context? | | | NI | No relevant information reported |
|  | 2.4 If Y/PY to 2.3: Were these deviations likely to have affected the outcome? | | | NA |  |
|  | 2.5. If Y/PY/NI to 2.4: Were these deviations from intended intervention balanced between groups? | | | NA |  |
|  | 2.6 Was an appropriate analysis used to estimate the effect of assignment to intervention? | | | NI | not enough information |
|  | 2.7 If N/PN/NI to 2.6: Was there potential for a substantial impact (on the result) of the failure to analyse participants in the group to which they were randomized? | | | NI | not enough information |
|  | **Risk of bias judgement** |  |  | **High** |  |
| **Bias due to missing outcome data** | 3.1 Were data for this outcome available for all, or nearly all, participants randomized? | | | Y | The authors report data on all patients involved in randomization |
|  | 3.2 If N/PN/NI to 3.1: Is there evidence that result was not biased by missing outcome data? | | | NA |  |
|  | 3.3 If N/PN to 3.2: Could missingness in the outcome depend on its true value? | | | NA |  |
|  | 3.4 If Y/PY/NI to 3.3: Is it likely that missingness in the outcome depended on its true value? | | | NA |  |
|  | **Risk of bias judgement** |  |  | **Low** |  |
| **Bias in measurement of the outcome** | 4.1 Was the method of measuring the outcome inappropriate? | | | N | method is appropriate |
|  | 4.2 Could measurement or ascertainment of the outcome have differed between intervention groups? | | | N | Outcome measures (data collection) involving the same measurement methods and thresholds, used at comparable time points |
|  | 4.3 Were outcome assessors aware of the intervention received by study participants? | | | NI | not enough information |
|  | 4.4 If Y/PY/NI to 4.3: Could assessment of the outcome have been influenced by knowledge of intervention received? | | | N | Results do not involve judgment |
|  | 4.5 If Y/PY/NI to 4.4: Is it likely that assessment of the outcome was influenced by knowledge of intervention received? | | | NA |  |
|  | **Risk of bias judgement** |  |  | **Low** |  |
| **Bias in selection of the reported result** | 5.1 Were the data that produced this result analysed in accordance with a pre-specified analysis plan that was finalized before unblinded outcome data were available for analysis? | | | NI | No mention of pre-specified analyses or protocols |
|  | 5.2 ... multiple eligible outcome measurements (e.g. scales, definitions, time points) within the outcome domain? | | | NI | not enough information |
|  | 5.3 ... multiple eligible analyses of the data? | |  | NI | not enough information |
|  | **Risk of bias judgement** |  |  | **Some concerns** |  |
| **Overall bias** | **Risk of bias judgement** |  |  | **High** |  |
|  |  |  |  |  |  |
|  |  |  |  |  |  |
| **Unique ID** | A11 | **Study ID** | Liu, 2016 | **Assessor** |  |
| **Ref or Label** |  | **Aim** | assignment to intervention (the 'intention-to-treat' effect) | |  |
| **Experimental** | GZFL+MFP | **Comparator** | MFP | **Source** | Journal article(s) with results of the trial |
| **Outcome** | CER | **Results** |  | **Weight** | 1 |
| **Domain** | **Signalling question** |  |  | **Response** | **Comments** |
| **Bias arising from the randomization process** | 1.1 Was the allocation sequence random? | |  | Y | The author uses the random number table method |
|  | 1.2 Was the allocation sequence concealed until participants were enrolled and assigned to interventions? | | | Y |  |
|  | 1.3 Did baseline differences between intervention groups suggest a problem with the randomization process? | | | N | There is no difference between baseline data |
|  | **Risk of bias judgement** |  |  | **Low** |  |
| **Bias due to deviations from intended interventions** | 2.1.Were participants aware of their assigned intervention during the trial? | | | Y | Does not mention whether blinding is used |
|  | 2.2.Were carers and people delivering the interventions aware of participants' assigned intervention during the trial? | | | Y |  |
|  | 2.3. If Y/PY/NI to 2.1 or 2.2: Were there deviations from the intended intervention that arose because of the experimental context? | | | NI | No relevant information reported |
|  | 2.4 If Y/PY to 2.3: Were these deviations likely to have affected the outcome? | | | NA |  |
|  | 2.5. If Y/PY/NI to 2.4: Were these deviations from intended intervention balanced between groups? | | | NA |  |
|  | 2.6 Was an appropriate analysis used to estimate the effect of assignment to intervention? | | | NI | not enough information |
|  | 2.7 If N/PN/NI to 2.6: Was there potential for a substantial impact (on the result) of the failure to analyse participants in the group to which they were randomized? | | | NI | not enough information |
|  | **Risk of bias judgement** |  |  | **High** |  |
| **Bias due to missing outcome data** | 3.1 Were data for this outcome available for all, or nearly all, participants randomized? | | | Y | The authors report data on all patients involved in randomization |
|  | 3.2 If N/PN/NI to 3.1: Is there evidence that result was not biased by missing outcome data? | | | NA |  |
|  | 3.3 If N/PN to 3.2: Could missingness in the outcome depend on its true value? | | | NA |  |
|  | 3.4 If Y/PY/NI to 3.3: Is it likely that missingness in the outcome depended on its true value? | | | NA |  |
|  | **Risk of bias judgement** |  |  | **Low** |  |
| **Bias in measurement of the outcome** | 4.1 Was the method of measuring the outcome inappropriate? | | | N | method is appropriate |
|  | 4.2 Could measurement or ascertainment of the outcome have differed between intervention groups? | | | N | Outcome measures (data collection) involving the same measurement methods and thresholds, used at comparable time points |
|  | 4.3 Were outcome assessors aware of the intervention received by study participants? | | | NI | not enough information |
|  | 4.4 If Y/PY/NI to 4.3: Could assessment of the outcome have been influenced by knowledge of intervention received? | | | N | Results do not involve judgment |
|  | 4.5 If Y/PY/NI to 4.4: Is it likely that assessment of the outcome was influenced by knowledge of intervention received? | | | NA |  |
|  | **Risk of bias judgement** |  |  | **Low** |  |
| **Bias in selection of the reported result** | 5.1 Were the data that produced this result analysed in accordance with a pre-specified analysis plan that was finalized before unblinded outcome data were available for analysis? | | | NI | No mention of pre-specified analyses or protocols |
|  | 5.2 ... multiple eligible outcome measurements (e.g. scales, definitions, time points) within the outcome domain? | | | NI | not enough information |
|  | 5.3 ... multiple eligible analyses of the data? | |  | NI | not enough information |
|  | **Risk of bias judgement** |  |  | **Some concerns** |  |
| **Overall bias** | **Risk of bias judgement** |  |  | **High** |  |
|  |  |  |  |  |  |
|  |  |  |  |  |  |
| **Unique ID** | A12 | **Study ID** | Luo, 2012 | **Assessor** |  |
| **Ref or Label** |  | **Aim** | assignment to intervention (the 'intention-to-treat' effect) | |  |
| **Experimental** | GZFL+MFP | **Comparator** | MFP | **Source** | Journal article(s) with results of the trial |
| **Outcome** | CER | **Results** |  | **Weight** | 1 |
| **Domain** | **Signalling question** |  |  | **Response** | **Comments** |
| **Bias arising from the randomization process** | 1.1 Was the allocation sequence random? | |  | NI | The authors did not describe in detail the generation and allocation concealment of random sequences |
|  | 1.2 Was the allocation sequence concealed until participants were enrolled and assigned to interventions? | | | NI |  |
|  | 1.3 Did baseline differences between intervention groups suggest a problem with the randomization process? | | | N | There is no difference between baseline data |
|  | **Risk of bias judgement** |  |  | **Some concerns** |  |
| **Bias due to deviations from intended interventions** | 2.1.Were participants aware of their assigned intervention during the trial? | | | Y | Does not mention whether blinding is used |
|  | 2.2.Were carers and people delivering the interventions aware of participants' assigned intervention during the trial? | | | Y |  |
|  | 2.3. If Y/PY/NI to 2.1 or 2.2: Were there deviations from the intended intervention that arose because of the experimental context? | | | NI | No relevant information reported |
|  | 2.4 If Y/PY to 2.3: Were these deviations likely to have affected the outcome? | | | NA |  |
|  | 2.5. If Y/PY/NI to 2.4: Were these deviations from intended intervention balanced between groups? | | | NA |  |
|  | 2.6 Was an appropriate analysis used to estimate the effect of assignment to intervention? | | | NI | not enough information |
|  | 2.7 If N/PN/NI to 2.6: Was there potential for a substantial impact (on the result) of the failure to analyse participants in the group to which they were randomized? | | | NI | not enough information |
|  | **Risk of bias judgement** |  |  | **High** |  |
| **Bias due to missing outcome data** | 3.1 Were data for this outcome available for all, or nearly all, participants randomized? | | | Y | The authors report data on all patients involved in randomization |
|  | 3.2 If N/PN/NI to 3.1: Is there evidence that result was not biased by missing outcome data? | | | NA |  |
|  | 3.3 If N/PN to 3.2: Could missingness in the outcome depend on its true value? | | | NA |  |
|  | 3.4 If Y/PY/NI to 3.3: Is it likely that missingness in the outcome depended on its true value? | | | NA |  |
|  | **Risk of bias judgement** |  |  | **Low** |  |
| **Bias in measurement of the outcome** | 4.1 Was the method of measuring the outcome inappropriate? | | | N | method is appropriate |
|  | 4.2 Could measurement or ascertainment of the outcome have differed between intervention groups? | | | N | Outcome measures (data collection) involving the same measurement methods and thresholds, used at comparable time points |
|  | 4.3 Were outcome assessors aware of the intervention received by study participants? | | | NI | not enough information |
|  | 4.4 If Y/PY/NI to 4.3: Could assessment of the outcome have been influenced by knowledge of intervention received? | | | N | Results do not involve judgment |
|  | 4.5 If Y/PY/NI to 4.4: Is it likely that assessment of the outcome was influenced by knowledge of intervention received? | | | NA |  |
|  | **Risk of bias judgement** |  |  | **Low** |  |
| **Bias in selection of the reported result** | 5.1 Were the data that produced this result analysed in accordance with a pre-specified analysis plan that was finalized before unblinded outcome data were available for analysis? | | | NI | No mention of pre-specified analyses or protocols |
|  | 5.2 ... multiple eligible outcome measurements (e.g. scales, definitions, time points) within the outcome domain? | | | NI | not enough information |
|  | 5.3 ... multiple eligible analyses of the data? | |  | NI | not enough information |
|  | **Risk of bias judgement** |  |  | **Some concerns** |  |
| **Overall bias** | **Risk of bias judgement** |  |  | **High** |  |
|  |  |  |  |  |  |
|  |  |  |  |  |  |
| **Unique ID** | A13 | **Study ID** | OU and Lan, 2015 | **Assessor** |  |
| **Ref or Label** |  | **Aim** | assignment to intervention (the 'intention-to-treat' effect) | |  |
| **Experimental** | GZFL+MFP | **Comparator** | MFP | **Source** | Journal article(s) with results of the trial |
| **Outcome** | CER | **Results** |  | **Weight** | 1 |
| **Domain** | **Signalling question** |  |  | **Response** | **Comments** |
| **Bias arising from the randomization process** | 1.1 Was the allocation sequence random? | |  | Y | The author uses the random number table method |
|  | 1.2 Was the allocation sequence concealed until participants were enrolled and assigned to interventions? | | | Y |  |
|  | 1.3 Did baseline differences between intervention groups suggest a problem with the randomization process? | | | N | There is no difference between baseline data |
|  | **Risk of bias judgement** |  |  | **Low** |  |
| **Bias due to deviations from intended interventions** | 2.1.Were participants aware of their assigned intervention during the trial? | | | Y | Does not mention whether blinding is used |
|  | 2.2.Were carers and people delivering the interventions aware of participants' assigned intervention during the trial? | | | Y |  |
|  | 2.3. If Y/PY/NI to 2.1 or 2.2: Were there deviations from the intended intervention that arose because of the experimental context? | | | NI | No relevant information reported |
|  | 2.4 If Y/PY to 2.3: Were these deviations likely to have affected the outcome? | | | NA |  |
|  | 2.5. If Y/PY/NI to 2.4: Were these deviations from intended intervention balanced between groups? | | | NA |  |
|  | 2.6 Was an appropriate analysis used to estimate the effect of assignment to intervention? | | | NI | not enough information |
|  | 2.7 If N/PN/NI to 2.6: Was there potential for a substantial impact (on the result) of the failure to analyse participants in the group to which they were randomized? | | | NI | not enough information |
|  | **Risk of bias judgement** |  |  | **High** |  |
| **Bias due to missing outcome data** | 3.1 Were data for this outcome available for all, or nearly all, participants randomized? | | | Y | The authors report data on all patients involved in randomization |
|  | 3.2 If N/PN/NI to 3.1: Is there evidence that result was not biased by missing outcome data? | | | NA |  |
|  | 3.3 If N/PN to 3.2: Could missingness in the outcome depend on its true value? | | | NA |  |
|  | 3.4 If Y/PY/NI to 3.3: Is it likely that missingness in the outcome depended on its true value? | | | NA |  |
|  | **Risk of bias judgement** |  |  | **Low** |  |
| **Bias in measurement of the outcome** | 4.1 Was the method of measuring the outcome inappropriate? | | | N | method is appropriate |
|  | 4.2 Could measurement or ascertainment of the outcome have differed between intervention groups? | | | N | Outcome measures (data collection) involving the same measurement methods and thresholds, used at comparable time points |
|  | 4.3 Were outcome assessors aware of the intervention received by study participants? | | | NI | not enough information |
|  | 4.4 If Y/PY/NI to 4.3: Could assessment of the outcome have been influenced by knowledge of intervention received? | | | N | Results do not involve judgment |
|  | 4.5 If Y/PY/NI to 4.4: Is it likely that assessment of the outcome was influenced by knowledge of intervention received? | | | NA |  |
|  | **Risk of bias judgement** |  |  | **Low** |  |
| **Bias in selection of the reported result** | 5.1 Were the data that produced this result analysed in accordance with a pre-specified analysis plan that was finalized before unblinded outcome data were available for analysis? | | | NI | No mention of pre-specified analyses or protocols |
|  | 5.2 ... multiple eligible outcome measurements (e.g. scales, definitions, time points) within the outcome domain? | | | NI | not enough information |
|  | 5.3 ... multiple eligible analyses of the data? | |  | NI | not enough information |
|  | **Risk of bias judgement** |  |  | **Some concerns** |  |
| **Overall bias** | **Risk of bias judgement** |  |  | **High** |  |
|  |  |  |  |  |  |
|  |  |  |  |  |  |
| **Unique ID** | A14 | **Study ID** | Qin, 2015 | **Assessor** |  |
| **Ref or Label** |  | **Aim** | assignment to intervention (the 'intention-to-treat' effect) | |  |
| **Experimental** | GZFL+MFP | **Comparator** | MFP | **Source** | Journal article(s) with results of the trial |
| **Outcome** | CER | **Results** |  | **Weight** | 1 |
| **Domain** | **Signalling question** |  |  | **Response** | **Comments** |
| **Bias arising from the randomization process** | 1.1 Was the allocation sequence random? | |  | NI | The authors did not describe in detail the generation and allocation concealment of random sequences |
|  | 1.2 Was the allocation sequence concealed until participants were enrolled and assigned to interventions? | | | NI |  |
|  | 1.3 Did baseline differences between intervention groups suggest a problem with the randomization process? | | | N | There is no difference between baseline data |
|  | **Risk of bias judgement** |  |  | **Some concerns** |  |
| **Bias due to deviations from intended interventions** | 2.1.Were participants aware of their assigned intervention during the trial? | | | Y | Does not mention whether blinding is used |
|  | 2.2.Were carers and people delivering the interventions aware of participants' assigned intervention during the trial? | | | Y |  |
|  | 2.3. If Y/PY/NI to 2.1 or 2.2: Were there deviations from the intended intervention that arose because of the experimental context? | | | NI | No relevant information reported |
|  | 2.4 If Y/PY to 2.3: Were these deviations likely to have affected the outcome? | | | NA |  |
|  | 2.5. If Y/PY/NI to 2.4: Were these deviations from intended intervention balanced between groups? | | | NA |  |
|  | 2.6 Was an appropriate analysis used to estimate the effect of assignment to intervention? | | | NI | not enough information |
|  | 2.7 If N/PN/NI to 2.6: Was there potential for a substantial impact (on the result) of the failure to analyse participants in the group to which they were randomized? | | | NI | not enough information |
|  | **Risk of bias judgement** |  |  | **High** |  |
| **Bias due to missing outcome data** | 3.1 Were data for this outcome available for all, or nearly all, participants randomized? | | | Y | The authors report data on all patients involved in randomization |
|  | 3.2 If N/PN/NI to 3.1: Is there evidence that result was not biased by missing outcome data? | | | NA |  |
|  | 3.3 If N/PN to 3.2: Could missingness in the outcome depend on its true value? | | | NA |  |
|  | 3.4 If Y/PY/NI to 3.3: Is it likely that missingness in the outcome depended on its true value? | | | NA |  |
|  | **Risk of bias judgement** |  |  | **Low** |  |
| **Bias in measurement of the outcome** | 4.1 Was the method of measuring the outcome inappropriate? | | | N | method is appropriate |
|  | 4.2 Could measurement or ascertainment of the outcome have differed between intervention groups? | | | N | Outcome measures (data collection) involving the same measurement methods and thresholds, used at comparable time points |
|  | 4.3 Were outcome assessors aware of the intervention received by study participants? | | | NI | not enough information |
|  | 4.4 If Y/PY/NI to 4.3: Could assessment of the outcome have been influenced by knowledge of intervention received? | | | N | Results do not involve judgment |
|  | 4.5 If Y/PY/NI to 4.4: Is it likely that assessment of the outcome was influenced by knowledge of intervention received? | | | NA |  |
|  | **Risk of bias judgement** |  |  | **Low** |  |
| **Bias in selection of the reported result** | 5.1 Were the data that produced this result analysed in accordance with a pre-specified analysis plan that was finalized before unblinded outcome data were available for analysis? | | | NI | No mention of pre-specified analyses or protocols |
|  | 5.2 ... multiple eligible outcome measurements (e.g. scales, definitions, time points) within the outcome domain? | | | NI | not enough information |
|  | 5.3 ... multiple eligible analyses of the data? | |  | NI | not enough information |
|  | **Risk of bias judgement** |  |  | **Some concerns** |  |
| **Overall bias** | **Risk of bias judgement** |  |  | **High** |  |
|  |  |  |  |  |  |
|  |  |  |  |  |  |
| **Unique ID** | A15 | **Study ID** | Sha and Zhu, 2016 | **Assessor** |  |
| **Ref or Label** |  | **Aim** | assignment to intervention (the 'intention-to-treat' effect) | |  |
| **Experimental** | GZFL+MFP | **Comparator** | MFP | **Source** | Journal article(s) with results of the trial |
| **Outcome** | CER | **Results** |  | **Weight** | 1 |
| **Domain** | **Signalling question** |  |  | **Response** | **Comments** |
| **Bias arising from the randomization process** | 1.1 Was the allocation sequence random? | |  | NI | The authors did not describe in detail the generation and allocation concealment of random sequences |
|  | 1.2 Was the allocation sequence concealed until participants were enrolled and assigned to interventions? | | | NI |  |
|  | 1.3 Did baseline differences between intervention groups suggest a problem with the randomization process? | | | N | There is no difference between baseline data |
|  | **Risk of bias judgement** |  |  | **Some concerns** |  |
| **Bias due to deviations from intended interventions** | 2.1.Were participants aware of their assigned intervention during the trial? | | | Y | Does not mention whether blinding is used |
|  | 2.2.Were carers and people delivering the interventions aware of participants' assigned intervention during the trial? | | | Y |  |
|  | 2.3. If Y/PY/NI to 2.1 or 2.2: Were there deviations from the intended intervention that arose because of the experimental context? | | | NI | No relevant information reported |
|  | 2.4 If Y/PY to 2.3: Were these deviations likely to have affected the outcome? | | | NA |  |
|  | 2.5. If Y/PY/NI to 2.4: Were these deviations from intended intervention balanced between groups? | | | NA |  |
|  | 2.6 Was an appropriate analysis used to estimate the effect of assignment to intervention? | | | NI | not enough information |
|  | 2.7 If N/PN/NI to 2.6: Was there potential for a substantial impact (on the result) of the failure to analyse participants in the group to which they were randomized? | | | NI | not enough information |
|  | **Risk of bias judgement** |  |  | **High** |  |
| **Bias due to missing outcome data** | 3.1 Were data for this outcome available for all, or nearly all, participants randomized? | | | Y | The authors report data on all patients involved in randomization |
|  | 3.2 If N/PN/NI to 3.1: Is there evidence that result was not biased by missing outcome data? | | | NA |  |
|  | 3.3 If N/PN to 3.2: Could missingness in the outcome depend on its true value? | | | NA |  |
|  | 3.4 If Y/PY/NI to 3.3: Is it likely that missingness in the outcome depended on its true value? | | | NA |  |
|  | **Risk of bias judgement** |  |  | **Low** |  |
| **Bias in measurement of the outcome** | 4.1 Was the method of measuring the outcome inappropriate? | | | N | method is appropriate |
|  | 4.2 Could measurement or ascertainment of the outcome have differed between intervention groups? | | | N | Outcome measures (data collection) involving the same measurement methods and thresholds, used at comparable time points |
|  | 4.3 Were outcome assessors aware of the intervention received by study participants? | | | NI | not enough information |
|  | 4.4 If Y/PY/NI to 4.3: Could assessment of the outcome have been influenced by knowledge of intervention received? | | | N | Results do not involve judgment |
|  | 4.5 If Y/PY/NI to 4.4: Is it likely that assessment of the outcome was influenced by knowledge of intervention received? | | | NA |  |
|  | **Risk of bias judgement** |  |  | **Low** |  |
| **Bias in selection of the reported result** | 5.1 Were the data that produced this result analysed in accordance with a pre-specified analysis plan that was finalized before unblinded outcome data were available for analysis? | | | NI | No mention of pre-specified analyses or protocols |
|  | 5.2 ... multiple eligible outcome measurements (e.g. scales, definitions, time points) within the outcome domain? | | | NI | not enough information |
|  | 5.3 ... multiple eligible analyses of the data? | |  | NI | not enough information |
|  | **Risk of bias judgement** |  |  | **Some concerns** |  |
| **Overall bias** | **Risk of bias judgement** |  |  | **High** |  |
|  |  |  |  |  |  |
|  |  |  |  |  |  |
| **Unique ID** | A16 | **Study ID** | Si et al., 2019 | **Assessor** |  |
| **Ref or Label** |  | **Aim** | assignment to intervention (the 'intention-to-treat' effect) | |  |
| **Experimental** | GZFL+MFP | **Comparator** | MFP | **Source** | Journal article(s) with results of the trial |
| **Outcome** | CER | **Results** |  | **Weight** | 1 |
| **Domain** | **Signalling question** |  |  | **Response** | **Comments** |
| **Bias arising from the randomization process** | 1.1 Was the allocation sequence random? | |  | NI | The authors did not describe in detail the generation and allocation concealment of random sequences |
|  | 1.2 Was the allocation sequence concealed until participants were enrolled and assigned to interventions? | | | NI |  |
|  | 1.3 Did baseline differences between intervention groups suggest a problem with the randomization process? | | | N | There is no difference between baseline data |
|  | **Risk of bias judgement** |  |  | **Some concerns** |  |
| **Bias due to deviations from intended interventions** | 2.1.Were participants aware of their assigned intervention during the trial? | | | Y | Does not mention whether blinding is used |
|  | 2.2.Were carers and people delivering the interventions aware of participants' assigned intervention during the trial? | | | Y |  |
|  | 2.3. If Y/PY/NI to 2.1 or 2.2: Were there deviations from the intended intervention that arose because of the experimental context? | | | NI | No relevant information reported |
|  | 2.4 If Y/PY to 2.3: Were these deviations likely to have affected the outcome? | | | NA |  |
|  | 2.5. If Y/PY/NI to 2.4: Were these deviations from intended intervention balanced between groups? | | | NA |  |
|  | 2.6 Was an appropriate analysis used to estimate the effect of assignment to intervention? | | | NI | not enough information |
|  | 2.7 If N/PN/NI to 2.6: Was there potential for a substantial impact (on the result) of the failure to analyse participants in the group to which they were randomized? | | | NI | not enough information |
|  | **Risk of bias judgement** |  |  | **High** |  |
| **Bias due to missing outcome data** | 3.1 Were data for this outcome available for all, or nearly all, participants randomized? | | | Y | The authors report data on all patients involved in randomization |
|  | 3.2 If N/PN/NI to 3.1: Is there evidence that result was not biased by missing outcome data? | | | NA |  |
|  | 3.3 If N/PN to 3.2: Could missingness in the outcome depend on its true value? | | | NA |  |
|  | 3.4 If Y/PY/NI to 3.3: Is it likely that missingness in the outcome depended on its true value? | | | NA |  |
|  | **Risk of bias judgement** |  |  | **Low** |  |
| **Bias in measurement of the outcome** | 4.1 Was the method of measuring the outcome inappropriate? | | | N | method is appropriate |
|  | 4.2 Could measurement or ascertainment of the outcome have differed between intervention groups? | | | N | Outcome measures (data collection) involving the same measurement methods and thresholds, used at comparable time points |
|  | 4.3 Were outcome assessors aware of the intervention received by study participants? | | | NI | not enough information |
|  | 4.4 If Y/PY/NI to 4.3: Could assessment of the outcome have been influenced by knowledge of intervention received? | | | N | Results do not involve judgment |
|  | 4.5 If Y/PY/NI to 4.4: Is it likely that assessment of the outcome was influenced by knowledge of intervention received? | | | NA |  |
|  | **Risk of bias judgement** |  |  | **Low** |  |
| **Bias in selection of the reported result** | 5.1 Were the data that produced this result analysed in accordance with a pre-specified analysis plan that was finalized before unblinded outcome data were available for analysis? | | | NI | No mention of pre-specified analyses or protocols |
|  | 5.2 ... multiple eligible outcome measurements (e.g. scales, definitions, time points) within the outcome domain? | | | NI | not enough information |
|  | 5.3 ... multiple eligible analyses of the data? | |  | NI | not enough information |
|  | **Risk of bias judgement** |  |  | **Some concerns** |  |
| **Overall bias** | **Risk of bias judgement** |  |  | **High** |  |
|  |  |  |  |  |  |
|  |  |  |  |  |  |
| **Unique ID** | A17 | **Study ID** | Su, 2013 | **Assessor** |  |
| **Ref or Label** |  | **Aim** | assignment to intervention (the 'intention-to-treat' effect) | |  |
| **Experimental** | GZFL+MFP | **Comparator** | MFP | **Source** | Journal article(s) with results of the trial |
| **Outcome** | CER | **Results** |  | **Weight** | 1 |
| **Domain** | **Signalling question** |  |  | **Response** | **Comments** |
| **Bias arising from the randomization process** | 1.1 Was the allocation sequence random? | |  | NI | The authors did not describe in detail the generation and allocation concealment of random sequences |
|  | 1.2 Was the allocation sequence concealed until participants were enrolled and assigned to interventions? | | | NI |  |
|  | 1.3 Did baseline differences between intervention groups suggest a problem with the randomization process? | | | N | There is no difference between baseline data |
|  | **Risk of bias judgement** |  |  | **Some concerns** |  |
| **Bias due to deviations from intended interventions** | 2.1.Were participants aware of their assigned intervention during the trial? | | | Y | Does not mention whether blinding is used |
|  | 2.2.Were carers and people delivering the interventions aware of participants' assigned intervention during the trial? | | | Y |  |
|  | 2.3. If Y/PY/NI to 2.1 or 2.2: Were there deviations from the intended intervention that arose because of the experimental context? | | | NI | No relevant information reported |
|  | 2.4 If Y/PY to 2.3: Were these deviations likely to have affected the outcome? | | | NA |  |
|  | 2.5. If Y/PY/NI to 2.4: Were these deviations from intended intervention balanced between groups? | | | NA |  |
|  | 2.6 Was an appropriate analysis used to estimate the effect of assignment to intervention? | | | NI | not enough information |
|  | 2.7 If N/PN/NI to 2.6: Was there potential for a substantial impact (on the result) of the failure to analyse participants in the group to which they were randomized? | | | NI | not enough information |
|  | **Risk of bias judgement** |  |  | **High** |  |
| **Bias due to missing outcome data** | 3.1 Were data for this outcome available for all, or nearly all, participants randomized? | | | Y | The authors report data on all patients involved in randomization |
|  | 3.2 If N/PN/NI to 3.1: Is there evidence that result was not biased by missing outcome data? | | | NA |  |
|  | 3.3 If N/PN to 3.2: Could missingness in the outcome depend on its true value? | | | NA |  |
|  | 3.4 If Y/PY/NI to 3.3: Is it likely that missingness in the outcome depended on its true value? | | | NA |  |
|  | **Risk of bias judgement** |  |  | **Low** |  |
| **Bias in measurement of the outcome** | 4.1 Was the method of measuring the outcome inappropriate? | | | N | method is appropriate |
|  | 4.2 Could measurement or ascertainment of the outcome have differed between intervention groups? | | | N | Outcome measures (data collection) involving the same measurement methods and thresholds, used at comparable time points |
|  | 4.3 Were outcome assessors aware of the intervention received by study participants? | | | NI | not enough information |
|  | 4.4 If Y/PY/NI to 4.3: Could assessment of the outcome have been influenced by knowledge of intervention received? | | | N | Results do not involve judgment |
|  | 4.5 If Y/PY/NI to 4.4: Is it likely that assessment of the outcome was influenced by knowledge of intervention received? | | | NA |  |
|  | **Risk of bias judgement** |  |  | **Low** |  |
| **Bias in selection of the reported result** | 5.1 Were the data that produced this result analysed in accordance with a pre-specified analysis plan that was finalized before unblinded outcome data were available for analysis? | | | NI | No mention of pre-specified analyses or protocols |
|  | 5.2 ... multiple eligible outcome measurements (e.g. scales, definitions, time points) within the outcome domain? | | | NI | not enough information |
|  | 5.3 ... multiple eligible analyses of the data? | |  | NI | not enough information |
|  | **Risk of bias judgement** |  |  | **Some concerns** |  |
| **Overall bias** | **Risk of bias judgement** |  |  | **High** |  |
|  |  |  |  |  |  |
|  |  |  |  |  |  |
| **Unique ID** | A18 | **Study ID** | Wang and Zhang, 2021 | **Assessor** |  |
| **Ref or Label** |  | **Aim** | assignment to intervention (the 'intention-to-treat' effect) | |  |
| **Experimental** | GZFL+MFP | **Comparator** | MFP | **Source** | Journal article(s) with results of the trial |
| **Outcome** | CER | **Results** |  | **Weight** | 1 |
| **Domain** | **Signalling question** |  |  | **Response** | **Comments** |
| **Bias arising from the randomization process** | 1.1 Was the allocation sequence random? | |  | NI | The authors did not describe in detail the generation and allocation concealment of random sequences |
|  | 1.2 Was the allocation sequence concealed until participants were enrolled and assigned to interventions? | | | NI |  |
|  | 1.3 Did baseline differences between intervention groups suggest a problem with the randomization process? | | | N | There is no difference between baseline data |
|  | **Risk of bias judgement** |  |  | **Some concerns** |  |
| **Bias due to deviations from intended interventions** | 2.1.Were participants aware of their assigned intervention during the trial? | | | Y | Does not mention whether blinding is used |
|  | 2.2.Were carers and people delivering the interventions aware of participants' assigned intervention during the trial? | | | Y |  |
|  | 2.3. If Y/PY/NI to 2.1 or 2.2: Were there deviations from the intended intervention that arose because of the experimental context? | | | NI | No relevant information reported |
|  | 2.4 If Y/PY to 2.3: Were these deviations likely to have affected the outcome? | | | NA |  |
|  | 2.5. If Y/PY/NI to 2.4: Were these deviations from intended intervention balanced between groups? | | | NA |  |
|  | 2.6 Was an appropriate analysis used to estimate the effect of assignment to intervention? | | | NI | not enough information |
|  | 2.7 If N/PN/NI to 2.6: Was there potential for a substantial impact (on the result) of the failure to analyse participants in the group to which they were randomized? | | | NI | not enough information |
|  | **Risk of bias judgement** |  |  | **High** |  |
| **Bias due to missing outcome data** | 3.1 Were data for this outcome available for all, or nearly all, participants randomized? | | | Y | The authors report data on all patients involved in randomization |
|  | 3.2 If N/PN/NI to 3.1: Is there evidence that result was not biased by missing outcome data? | | | NA |  |
|  | 3.3 If N/PN to 3.2: Could missingness in the outcome depend on its true value? | | | NA |  |
|  | 3.4 If Y/PY/NI to 3.3: Is it likely that missingness in the outcome depended on its true value? | | | NA |  |
|  | **Risk of bias judgement** |  |  | **Low** |  |
| **Bias in measurement of the outcome** | 4.1 Was the method of measuring the outcome inappropriate? | | | N | method is appropriate |
|  | 4.2 Could measurement or ascertainment of the outcome have differed between intervention groups? | | | N | Outcome measures (data collection) involving the same measurement methods and thresholds, used at comparable time points |
|  | 4.3 Were outcome assessors aware of the intervention received by study participants? | | | NI | not enough information |
|  | 4.4 If Y/PY/NI to 4.3: Could assessment of the outcome have been influenced by knowledge of intervention received? | | | N | Results do not involve judgment |
|  | 4.5 If Y/PY/NI to 4.4: Is it likely that assessment of the outcome was influenced by knowledge of intervention received? | | | NA |  |
|  | **Risk of bias judgement** |  |  | **Low** |  |
| **Bias in selection of the reported result** | 5.1 Were the data that produced this result analysed in accordance with a pre-specified analysis plan that was finalized before unblinded outcome data were available for analysis? | | | NI | No mention of pre-specified analyses or protocols |
|  | 5.2 ... multiple eligible outcome measurements (e.g. scales, definitions, time points) within the outcome domain? | | | NI | not enough information |
|  | 5.3 ... multiple eligible analyses of the data? | |  | NI | not enough information |
|  | **Risk of bias judgement** |  |  | **Some concerns** |  |
| **Overall bias** | **Risk of bias judgement** |  |  | **High** |  |
|  |  |  |  |  |  |
|  |  |  |  |  |  |
| **Unique ID** | A19 | **Study ID** | Wei, 2019 | **Assessor** |  |
| **Ref or Label** |  | **Aim** | assignment to intervention (the 'intention-to-treat' effect) | |  |
| **Experimental** | GZFL+MFP | **Comparator** | MFP | **Source** | Journal article(s) with results of the trial |
| **Outcome** | CER | **Results** |  | **Weight** | 1 |
| **Domain** | **Signalling question** |  |  | **Response** | **Comments** |
| **Bias arising from the randomization process** | 1.1 Was the allocation sequence random? | |  | NI | The authors did not describe in detail the generation and allocation concealment of random sequences |
|  | 1.2 Was the allocation sequence concealed until participants were enrolled and assigned to interventions? | | | NI |  |
|  | 1.3 Did baseline differences between intervention groups suggest a problem with the randomization process? | | | N | There is no difference between baseline data |
|  | **Risk of bias judgement** |  |  | **Some concerns** |  |
| **Bias due to deviations from intended interventions** | 2.1.Were participants aware of their assigned intervention during the trial? | | | Y | Does not mention whether blinding is used |
|  | 2.2.Were carers and people delivering the interventions aware of participants' assigned intervention during the trial? | | | Y |  |
|  | 2.3. If Y/PY/NI to 2.1 or 2.2: Were there deviations from the intended intervention that arose because of the experimental context? | | | NI | No relevant information reported |
|  | 2.4 If Y/PY to 2.3: Were these deviations likely to have affected the outcome? | | | NA |  |
|  | 2.5. If Y/PY/NI to 2.4: Were these deviations from intended intervention balanced between groups? | | | NA |  |
|  | 2.6 Was an appropriate analysis used to estimate the effect of assignment to intervention? | | | NI | not enough information |
|  | 2.7 If N/PN/NI to 2.6: Was there potential for a substantial impact (on the result) of the failure to analyse participants in the group to which they were randomized? | | | NI | not enough information |
|  | **Risk of bias judgement** |  |  | **High** |  |
| **Bias due to missing outcome data** | 3.1 Were data for this outcome available for all, or nearly all, participants randomized? | | | Y | The authors report data on all patients involved in randomization |
|  | 3.2 If N/PN/NI to 3.1: Is there evidence that result was not biased by missing outcome data? | | | NA |  |
|  | 3.3 If N/PN to 3.2: Could missingness in the outcome depend on its true value? | | | NA |  |
|  | 3.4 If Y/PY/NI to 3.3: Is it likely that missingness in the outcome depended on its true value? | | | NA |  |
|  | **Risk of bias judgement** |  |  | **Low** |  |
| **Bias in measurement of the outcome** | 4.1 Was the method of measuring the outcome inappropriate? | | | N | method is appropriate |
|  | 4.2 Could measurement or ascertainment of the outcome have differed between intervention groups? | | | N | Outcome measures (data collection) involving the same measurement methods and thresholds, used at comparable time points |
|  | 4.3 Were outcome assessors aware of the intervention received by study participants? | | | NI | not enough information |
|  | 4.4 If Y/PY/NI to 4.3: Could assessment of the outcome have been influenced by knowledge of intervention received? | | | N | Results do not involve judgment |
|  | 4.5 If Y/PY/NI to 4.4: Is it likely that assessment of the outcome was influenced by knowledge of intervention received? | | | NA |  |
|  | **Risk of bias judgement** |  |  | **Low** |  |
| **Bias in selection of the reported result** | 5.1 Were the data that produced this result analysed in accordance with a pre-specified analysis plan that was finalized before unblinded outcome data were available for analysis? | | | NI | No mention of pre-specified analyses or protocols |
|  | 5.2 ... multiple eligible outcome measurements (e.g. scales, definitions, time points) within the outcome domain? | | | NI | not enough information |
|  | 5.3 ... multiple eligible analyses of the data? | |  | NI | not enough information |
|  | **Risk of bias judgement** |  |  | **Some concerns** |  |
| **Overall bias** | **Risk of bias judgement** |  |  | **High** |  |
|  |  |  |  |  |  |
|  |  |  |  |  |  |
| **Unique ID** | A20 | **Study ID** | Wei, 2014 | **Assessor** |  |
| **Ref or Label** |  | **Aim** | assignment to intervention (the 'intention-to-treat' effect) | |  |
| **Experimental** | GZFL+MFP | **Comparator** | MFP | **Source** | Journal article(s) with results of the trial |
| **Outcome** | CER | **Results** |  | **Weight** | 1 |
| **Domain** | **Signalling question** |  |  | **Response** | **Comments** |
| **Bias arising from the randomization process** | 1.1 Was the allocation sequence random? | |  | NI | The authors did not describe in detail the generation and allocation concealment of random sequences |
|  | 1.2 Was the allocation sequence concealed until participants were enrolled and assigned to interventions? | | | NI |  |
|  | 1.3 Did baseline differences between intervention groups suggest a problem with the randomization process? | | | N | There is no difference between baseline data |
|  | **Risk of bias judgement** |  |  | **Some concerns** |  |
| **Bias due to deviations from intended interventions** | 2.1.Were participants aware of their assigned intervention during the trial? | | | Y | Does not mention whether blinding is used |
|  | 2.2.Were carers and people delivering the interventions aware of participants' assigned intervention during the trial? | | | Y |  |
|  | 2.3. If Y/PY/NI to 2.1 or 2.2: Were there deviations from the intended intervention that arose because of the experimental context? | | | NI | No relevant information reported |
|  | 2.4 If Y/PY to 2.3: Were these deviations likely to have affected the outcome? | | | NA |  |
|  | 2.5. If Y/PY/NI to 2.4: Were these deviations from intended intervention balanced between groups? | | | NA |  |
|  | 2.6 Was an appropriate analysis used to estimate the effect of assignment to intervention? | | | NI | not enough information |
|  | 2.7 If N/PN/NI to 2.6: Was there potential for a substantial impact (on the result) of the failure to analyse participants in the group to which they were randomized? | | | NI | not enough information |
|  | **Risk of bias judgement** |  |  | **High** |  |
| **Bias due to missing outcome data** | 3.1 Were data for this outcome available for all, or nearly all, participants randomized? | | | Y | The authors report data on all patients involved in randomization |
|  | 3.2 If N/PN/NI to 3.1: Is there evidence that result was not biased by missing outcome data? | | | NA |  |
|  | 3.3 If N/PN to 3.2: Could missingness in the outcome depend on its true value? | | | NA |  |
|  | 3.4 If Y/PY/NI to 3.3: Is it likely that missingness in the outcome depended on its true value? | | | NA |  |
|  | **Risk of bias judgement** |  |  | **Low** |  |
| **Bias in measurement of the outcome** | 4.1 Was the method of measuring the outcome inappropriate? | | | N | method is appropriate |
|  | 4.2 Could measurement or ascertainment of the outcome have differed between intervention groups? | | | N | Outcome measures (data collection) involving the same measurement methods and thresholds, used at comparable time points |
|  | 4.3 Were outcome assessors aware of the intervention received by study participants? | | | NI | not enough information |
|  | 4.4 If Y/PY/NI to 4.3: Could assessment of the outcome have been influenced by knowledge of intervention received? | | | N | Results do not involve judgment |
|  | 4.5 If Y/PY/NI to 4.4: Is it likely that assessment of the outcome was influenced by knowledge of intervention received? | | | NA |  |
|  | **Risk of bias judgement** |  |  | **Low** |  |
| **Bias in selection of the reported result** | 5.1 Were the data that produced this result analysed in accordance with a pre-specified analysis plan that was finalized before unblinded outcome data were available for analysis? | | | NI | No mention of pre-specified analyses or protocols |
|  | 5.2 ... multiple eligible outcome measurements (e.g. scales, definitions, time points) within the outcome domain? | | | NI | not enough information |
|  | 5.3 ... multiple eligible analyses of the data? | |  | NI | not enough information |
|  | **Risk of bias judgement** |  |  | **Some concerns** |  |
| **Overall bias** | **Risk of bias judgement** |  |  | **High** |  |
|  |  |  |  |  |  |
|  |  |  |  |  |  |
| **Unique ID** | A21 | **Study ID** | Xu, 2017 | **Assessor** |  |
| **Ref or Label** |  | **Aim** | assignment to intervention (the 'intention-to-treat' effect) | |  |
| **Experimental** | GZFL+MFP | **Comparator** | MFP | **Source** | Journal article(s) with results of the trial |
| **Outcome** | CER | **Results** |  | **Weight** | 1 |
| **Domain** | **Signalling question** |  |  | **Response** | **Comments** |
| **Bias arising from the randomization process** | 1.1 Was the allocation sequence random? | |  | Y | The author uses the random number table method |
|  | 1.2 Was the allocation sequence concealed until participants were enrolled and assigned to interventions? | | | Y |  |
|  | 1.3 Did baseline differences between intervention groups suggest a problem with the randomization process? | | | N | There is no difference between baseline data |
|  | **Risk of bias judgement** |  |  | **Low** |  |
| **Bias due to deviations from intended interventions** | 2.1.Were participants aware of their assigned intervention during the trial? | | | Y | Does not mention whether blinding is used |
|  | 2.2.Were carers and people delivering the interventions aware of participants' assigned intervention during the trial? | | | Y |  |
|  | 2.3. If Y/PY/NI to 2.1 or 2.2: Were there deviations from the intended intervention that arose because of the experimental context? | | | NI | No relevant information reported |
|  | 2.4 If Y/PY to 2.3: Were these deviations likely to have affected the outcome? | | | NA |  |
|  | 2.5. If Y/PY/NI to 2.4: Were these deviations from intended intervention balanced between groups? | | | NA |  |
|  | 2.6 Was an appropriate analysis used to estimate the effect of assignment to intervention? | | | NI | not enough information |
|  | 2.7 If N/PN/NI to 2.6: Was there potential for a substantial impact (on the result) of the failure to analyse participants in the group to which they were randomized? | | | NI | not enough information |
|  | **Risk of bias judgement** |  |  | **High** |  |
| **Bias due to missing outcome data** | 3.1 Were data for this outcome available for all, or nearly all, participants randomized? | | | Y | The authors report data on all patients involved in randomization |
|  | 3.2 If N/PN/NI to 3.1: Is there evidence that result was not biased by missing outcome data? | | | NA |  |
|  | 3.3 If N/PN to 3.2: Could missingness in the outcome depend on its true value? | | | NA |  |
|  | 3.4 If Y/PY/NI to 3.3: Is it likely that missingness in the outcome depended on its true value? | | | NA |  |
|  | **Risk of bias judgement** |  |  | **Low** |  |
| **Bias in measurement of the outcome** | 4.1 Was the method of measuring the outcome inappropriate? | | | N | method is appropriate |
|  | 4.2 Could measurement or ascertainment of the outcome have differed between intervention groups? | | | N | Outcome measures (data collection) involving the same measurement methods and thresholds, used at comparable time points |
|  | 4.3 Were outcome assessors aware of the intervention received by study participants? | | | NI | not enough information |
|  | 4.4 If Y/PY/NI to 4.3: Could assessment of the outcome have been influenced by knowledge of intervention received? | | | N | Results do not involve judgment |
|  | 4.5 If Y/PY/NI to 4.4: Is it likely that assessment of the outcome was influenced by knowledge of intervention received? | | | NA |  |
|  | **Risk of bias judgement** |  |  | **Low** |  |
| **Bias in selection of the reported result** | 5.1 Were the data that produced this result analysed in accordance with a pre-specified analysis plan that was finalized before unblinded outcome data were available for analysis? | | | NI | No mention of pre-specified analyses or protocols |
|  | 5.2 ... multiple eligible outcome measurements (e.g. scales, definitions, time points) within the outcome domain? | | | NI | not enough information |
|  | 5.3 ... multiple eligible analyses of the data? | |  | NI | not enough information |
|  | **Risk of bias judgement** |  |  | **Some concerns** |  |
| **Overall bias** | **Risk of bias judgement** |  |  | **High** |  |
|  |  |  |  |  |  |
|  |  |  |  |  |  |
| **Unique ID** | A22 | **Study ID** | Yang, 2018 | **Assessor** |  |
| **Ref or Label** |  | **Aim** | assignment to intervention (the 'intention-to-treat' effect) | |  |
| **Experimental** | GZFL+MFP | **Comparator** | MFP | **Source** | Journal article(s) with results of the trial |
| **Outcome** | CER | **Results** |  | **Weight** | 1 |
| **Domain** | **Signalling question** |  |  | **Response** | **Comments** |
| **Bias arising from the randomization process** | 1.1 Was the allocation sequence random? | |  | Y | The author uses the random number table method |
|  | 1.2 Was the allocation sequence concealed until participants were enrolled and assigned to interventions? | | | Y |  |
|  | 1.3 Did baseline differences between intervention groups suggest a problem with the randomization process? | | | N | There is no difference between baseline data |
|  | **Risk of bias judgement** |  |  | **Low** |  |
| **Bias due to deviations from intended interventions** | 2.1.Were participants aware of their assigned intervention during the trial? | | | Y | Does not mention whether blinding is used |
|  | 2.2.Were carers and people delivering the interventions aware of participants' assigned intervention during the trial? | | | Y |  |
|  | 2.3. If Y/PY/NI to 2.1 or 2.2: Were there deviations from the intended intervention that arose because of the experimental context? | | | NI | No relevant information reported |
|  | 2.4 If Y/PY to 2.3: Were these deviations likely to have affected the outcome? | | | NA |  |
|  | 2.5. If Y/PY/NI to 2.4: Were these deviations from intended intervention balanced between groups? | | | NA |  |
|  | 2.6 Was an appropriate analysis used to estimate the effect of assignment to intervention? | | | NI | not enough information |
|  | 2.7 If N/PN/NI to 2.6: Was there potential for a substantial impact (on the result) of the failure to analyse participants in the group to which they were randomized? | | | NI | not enough information |
|  | **Risk of bias judgement** |  |  | **High** |  |
| **Bias due to missing outcome data** | 3.1 Were data for this outcome available for all, or nearly all, participants randomized? | | | Y | The authors report data on all patients involved in randomization |
|  | 3.2 If N/PN/NI to 3.1: Is there evidence that result was not biased by missing outcome data? | | | NA |  |
|  | 3.3 If N/PN to 3.2: Could missingness in the outcome depend on its true value? | | | NA |  |
|  | 3.4 If Y/PY/NI to 3.3: Is it likely that missingness in the outcome depended on its true value? | | | NA |  |
|  | **Risk of bias judgement** |  |  | **Low** |  |
| **Bias in measurement of the outcome** | 4.1 Was the method of measuring the outcome inappropriate? | | | N | method is appropriate |
|  | 4.2 Could measurement or ascertainment of the outcome have differed between intervention groups? | | | N | Outcome measures (data collection) involving the same measurement methods and thresholds, used at comparable time points |
|  | 4.3 Were outcome assessors aware of the intervention received by study participants? | | | NI | not enough information |
|  | 4.4 If Y/PY/NI to 4.3: Could assessment of the outcome have been influenced by knowledge of intervention received? | | | N | Results do not involve judgment |
|  | 4.5 If Y/PY/NI to 4.4: Is it likely that assessment of the outcome was influenced by knowledge of intervention received? | | | NA |  |
|  | **Risk of bias judgement** |  |  | **Low** |  |
| **Bias in selection of the reported result** | 5.1 Were the data that produced this result analysed in accordance with a pre-specified analysis plan that was finalized before unblinded outcome data were available for analysis? | | | NI | No mention of pre-specified analyses or protocols |
|  | 5.2 ... multiple eligible outcome measurements (e.g. scales, definitions, time points) within the outcome domain? | | | NI | not enough information |
|  | 5.3 ... multiple eligible analyses of the data? | |  | NI | not enough information |
|  | **Risk of bias judgement** |  |  | **Some concerns** |  |
| **Overall bias** | **Risk of bias judgement** |  |  | **High** |  |
|  |  |  |  |  |  |
|  |  |  |  |  |  |
| **Unique ID** | A23 | **Study ID** | Yuan et al., 2021 | **Assessor** |  |
| **Ref or Label** |  | **Aim** | assignment to intervention (the 'intention-to-treat' effect) | |  |
| **Experimental** | GZFL+MFP | **Comparator** | MFP | **Source** | Journal article(s) with results of the trial |
| **Outcome** | CER | **Results** |  | **Weight** | 1 |
| **Domain** | **Signalling question** |  |  | **Response** | **Comments** |
| **Bias arising from the randomization process** | 1.1 Was the allocation sequence random? | |  | NI | The authors did not describe in detail the generation and allocation concealment of random sequences |
|  | 1.2 Was the allocation sequence concealed until participants were enrolled and assigned to interventions? | | | NI |  |
|  | 1.3 Did baseline differences between intervention groups suggest a problem with the randomization process? | | | N | There is no difference between baseline data |
|  | **Risk of bias judgement** |  |  | **Some concerns** |  |
| **Bias due to deviations from intended interventions** | 2.1.Were participants aware of their assigned intervention during the trial? | | | Y | Does not mention whether blinding is used |
|  | 2.2.Were carers and people delivering the interventions aware of participants' assigned intervention during the trial? | | | Y |  |
|  | 2.3. If Y/PY/NI to 2.1 or 2.2: Were there deviations from the intended intervention that arose because of the experimental context? | | | NI | No relevant information reported |
|  | 2.4 If Y/PY to 2.3: Were these deviations likely to have affected the outcome? | | | NA |  |
|  | 2.5. If Y/PY/NI to 2.4: Were these deviations from intended intervention balanced between groups? | | | NA |  |
|  | 2.6 Was an appropriate analysis used to estimate the effect of assignment to intervention? | | | NI | not enough information |
|  | 2.7 If N/PN/NI to 2.6: Was there potential for a substantial impact (on the result) of the failure to analyse participants in the group to which they were randomized? | | | NI | not enough information |
|  | **Risk of bias judgement** |  |  | **High** |  |
| **Bias due to missing outcome data** | 3.1 Were data for this outcome available for all, or nearly all, participants randomized? | | | Y | The authors report data on all patients involved in randomization |
|  | 3.2 If N/PN/NI to 3.1: Is there evidence that result was not biased by missing outcome data? | | | NA |  |
|  | 3.3 If N/PN to 3.2: Could missingness in the outcome depend on its true value? | | | NA |  |
|  | 3.4 If Y/PY/NI to 3.3: Is it likely that missingness in the outcome depended on its true value? | | | NA |  |
|  | **Risk of bias judgement** |  |  | **Low** |  |
| **Bias in measurement of the outcome** | 4.1 Was the method of measuring the outcome inappropriate? | | | N | method is appropriate |
|  | 4.2 Could measurement or ascertainment of the outcome have differed between intervention groups? | | | N | Outcome measures (data collection) involving the same measurement methods and thresholds, used at comparable time points |
|  | 4.3 Were outcome assessors aware of the intervention received by study participants? | | | NI | not enough information |
|  | 4.4 If Y/PY/NI to 4.3: Could assessment of the outcome have been influenced by knowledge of intervention received? | | | N | Results do not involve judgment |
|  | 4.5 If Y/PY/NI to 4.4: Is it likely that assessment of the outcome was influenced by knowledge of intervention received? | | | NA |  |
|  | **Risk of bias judgement** |  |  | **Low** |  |
| **Bias in selection of the reported result** | 5.1 Were the data that produced this result analysed in accordance with a pre-specified analysis plan that was finalized before unblinded outcome data were available for analysis? | | | NI | No mention of pre-specified analyses or protocols |
|  | 5.2 ... multiple eligible outcome measurements (e.g. scales, definitions, time points) within the outcome domain? | | | NI | not enough information |
|  | 5.3 ... multiple eligible analyses of the data? | |  | NI | not enough information |
|  | **Risk of bias judgement** |  |  | **Some concerns** |  |
| **Overall bias** | **Risk of bias judgement** |  |  | **High** |  |
|  |  |  |  |  |  |
|  |  |  |  |  |  |
| **Unique ID** | A24 | **Study ID** | Zhang and Yi, 2014 | **Assessor** |  |
| **Ref or Label** |  | **Aim** | assignment to intervention (the 'intention-to-treat' effect) | |  |
| **Experimental** | GZFL+MFP | **Comparator** | MFP | **Source** | Journal article(s) with results of the trial |
| **Outcome** | CER | **Results** |  | **Weight** | 1 |
| **Domain** | **Signalling question** |  |  | **Response** | **Comments** |
| **Bias arising from the randomization process** | 1.1 Was the allocation sequence random? | |  | NI | The authors did not describe in detail the generation and allocation concealment of random sequences |
|  | 1.2 Was the allocation sequence concealed until participants were enrolled and assigned to interventions? | | | NI |  |
|  | 1.3 Did baseline differences between intervention groups suggest a problem with the randomization process? | | | N | There is no difference between baseline data |
|  | **Risk of bias judgement** |  |  | **Some concerns** |  |
| **Bias due to deviations from intended interventions** | 2.1.Were participants aware of their assigned intervention during the trial? | | | Y | Does not mention whether blinding is used |
|  | 2.2.Were carers and people delivering the interventions aware of participants' assigned intervention during the trial? | | | Y |  |
|  | 2.3. If Y/PY/NI to 2.1 or 2.2: Were there deviations from the intended intervention that arose because of the experimental context? | | | NI | No relevant information reported |
|  | 2.4 If Y/PY to 2.3: Were these deviations likely to have affected the outcome? | | | NA |  |
|  | 2.5. If Y/PY/NI to 2.4: Were these deviations from intended intervention balanced between groups? | | | NA |  |
|  | 2.6 Was an appropriate analysis used to estimate the effect of assignment to intervention? | | | NI | not enough information |
|  | 2.7 If N/PN/NI to 2.6: Was there potential for a substantial impact (on the result) of the failure to analyse participants in the group to which they were randomized? | | | NI | not enough information |
|  | **Risk of bias judgement** |  |  | **High** |  |
| **Bias due to missing outcome data** | 3.1 Were data for this outcome available for all, or nearly all, participants randomized? | | | Y | The authors report data on all patients involved in randomization |
|  | 3.2 If N/PN/NI to 3.1: Is there evidence that result was not biased by missing outcome data? | | | NA |  |
|  | 3.3 If N/PN to 3.2: Could missingness in the outcome depend on its true value? | | | NA |  |
|  | 3.4 If Y/PY/NI to 3.3: Is it likely that missingness in the outcome depended on its true value? | | | NA |  |
|  | **Risk of bias judgement** |  |  | **Low** |  |
| **Bias in measurement of the outcome** | 4.1 Was the method of measuring the outcome inappropriate? | | | N | method is appropriate |
|  | 4.2 Could measurement or ascertainment of the outcome have differed between intervention groups? | | | N | Outcome measures (data collection) involving the same measurement methods and thresholds, used at comparable time points |
|  | 4.3 Were outcome assessors aware of the intervention received by study participants? | | | NI | not enough information |
|  | 4.4 If Y/PY/NI to 4.3: Could assessment of the outcome have been influenced by knowledge of intervention received? | | | N | Results do not involve judgment |
|  | 4.5 If Y/PY/NI to 4.4: Is it likely that assessment of the outcome was influenced by knowledge of intervention received? | | | NA |  |
|  | **Risk of bias judgement** |  |  | **Low** |  |
| **Bias in selection of the reported result** | 5.1 Were the data that produced this result analysed in accordance with a pre-specified analysis plan that was finalized before unblinded outcome data were available for analysis? | | | NI | No mention of pre-specified analyses or protocols |
|  | 5.2 ... multiple eligible outcome measurements (e.g. scales, definitions, time points) within the outcome domain? | | | NI | not enough information |
|  | 5.3 ... multiple eligible analyses of the data? | |  | NI | not enough information |
|  | **Risk of bias judgement** |  |  | **Some concerns** |  |
| **Overall bias** | **Risk of bias judgement** |  |  | **High** |  |
|  |  |  |  |  |  |
|  |  |  |  |  |  |
| **Unique ID** | A25 | **Study ID** | Zhang et al., 2019 | **Assessor** |  |
| **Ref or Label** |  | **Aim** | assignment to intervention (the 'intention-to-treat' effect) | |  |
| **Experimental** | GZFL+MFP | **Comparator** | MFP | **Source** | Journal article(s) with results of the trial |
| **Outcome** | CER | **Results** |  | **Weight** | 1 |
| **Domain** | **Signalling question** |  |  | **Response** | **Comments** |
| **Bias arising from the randomization process** | 1.1 Was the allocation sequence random? | |  | Y | The author uses the random number table method |
|  | 1.2 Was the allocation sequence concealed until participants were enrolled and assigned to interventions? | | | Y |  |
|  | 1.3 Did baseline differences between intervention groups suggest a problem with the randomization process? | | | N | There is no difference between baseline data |
|  | **Risk of bias judgement** |  |  | **Low** |  |
| **Bias due to deviations from intended interventions** | 2.1.Were participants aware of their assigned intervention during the trial? | | | Y | Does not mention whether blinding is used |
|  | 2.2.Were carers and people delivering the interventions aware of participants' assigned intervention during the trial? | | | Y |  |
|  | 2.3. If Y/PY/NI to 2.1 or 2.2: Were there deviations from the intended intervention that arose because of the experimental context? | | | NI | No relevant information reported |
|  | 2.4 If Y/PY to 2.3: Were these deviations likely to have affected the outcome? | | | NA |  |
|  | 2.5. If Y/PY/NI to 2.4: Were these deviations from intended intervention balanced between groups? | | | NA |  |
|  | 2.6 Was an appropriate analysis used to estimate the effect of assignment to intervention? | | | NI | not enough information |
|  | 2.7 If N/PN/NI to 2.6: Was there potential for a substantial impact (on the result) of the failure to analyse participants in the group to which they were randomized? | | | NI | not enough information |
|  | **Risk of bias judgement** |  |  | **High** |  |
| **Bias due to missing outcome data** | 3.1 Were data for this outcome available for all, or nearly all, participants randomized? | | | Y | The authors report data on all patients involved in randomization |
|  | 3.2 If N/PN/NI to 3.1: Is there evidence that result was not biased by missing outcome data? | | | NA |  |
|  | 3.3 If N/PN to 3.2: Could missingness in the outcome depend on its true value? | | | NA |  |
|  | 3.4 If Y/PY/NI to 3.3: Is it likely that missingness in the outcome depended on its true value? | | | NA |  |
|  | **Risk of bias judgement** |  |  | **Low** |  |
| **Bias in measurement of the outcome** | 4.1 Was the method of measuring the outcome inappropriate? | | | N | method is appropriate |
|  | 4.2 Could measurement or ascertainment of the outcome have differed between intervention groups? | | | N | Outcome measures (data collection) involving the same measurement methods and thresholds, used at comparable time points |
|  | 4.3 Were outcome assessors aware of the intervention received by study participants? | | | NI | not enough information |
|  | 4.4 If Y/PY/NI to 4.3: Could assessment of the outcome have been influenced by knowledge of intervention received? | | | N | Results do not involve judgment |
|  | 4.5 If Y/PY/NI to 4.4: Is it likely that assessment of the outcome was influenced by knowledge of intervention received? | | | NA |  |
|  | **Risk of bias judgement** |  |  | **Low** |  |
| **Bias in selection of the reported result** | 5.1 Were the data that produced this result analysed in accordance with a pre-specified analysis plan that was finalized before unblinded outcome data were available for analysis? | | | NI | No mention of pre-specified analyses or protocols |
|  | 5.2 ... multiple eligible outcome measurements (e.g. scales, definitions, time points) within the outcome domain? | | | NI | not enough information |
|  | 5.3 ... multiple eligible analyses of the data? | |  | NI | not enough information |
|  | **Risk of bias judgement** |  |  | **Some concerns** |  |
| **Overall bias** | **Risk of bias judgement** |  |  | **High** |  |
|  |  |  |  |  |  |
|  |  |  |  |  |  |
| **Unique ID** | A26 | **Study ID** | Zhong, 2020 | **Assessor** |  |
| **Ref or Label** |  | **Aim** | assignment to intervention (the 'intention-to-treat' effect) | |  |
| **Experimental** | GZFL+MFP | **Comparator** | MFP | **Source** | Journal article(s) with results of the trial |
| **Outcome** | CER | **Results** |  | **Weight** | 1 |
| **Domain** | **Signalling question** |  |  | **Response** | **Comments** |
| **Bias arising from the randomization process** | 1.1 Was the allocation sequence random? | |  | NI | The authors did not describe in detail the generation and allocation concealment of random sequences |
|  | 1.2 Was the allocation sequence concealed until participants were enrolled and assigned to interventions? | | | NI |  |
|  | 1.3 Did baseline differences between intervention groups suggest a problem with the randomization process? | | | N | There is no difference between baseline data |
|  | **Risk of bias judgement** |  |  | **Some concerns** |  |
| **Bias due to deviations from intended interventions** | 2.1.Were participants aware of their assigned intervention during the trial? | | | Y | Does not mention whether blinding is used |
|  | 2.2.Were carers and people delivering the interventions aware of participants' assigned intervention during the trial? | | | Y |  |
|  | 2.3. If Y/PY/NI to 2.1 or 2.2: Were there deviations from the intended intervention that arose because of the experimental context? | | | NI | No relevant information reported |
|  | 2.4 If Y/PY to 2.3: Were these deviations likely to have affected the outcome? | | | NA |  |
|  | 2.5. If Y/PY/NI to 2.4: Were these deviations from intended intervention balanced between groups? | | | NA |  |
|  | 2.6 Was an appropriate analysis used to estimate the effect of assignment to intervention? | | | NI | not enough information |
|  | 2.7 If N/PN/NI to 2.6: Was there potential for a substantial impact (on the result) of the failure to analyse participants in the group to which they were randomized? | | | NI | not enough information |
|  | **Risk of bias judgement** |  |  | **High** |  |
| **Bias due to missing outcome data** | 3.1 Were data for this outcome available for all, or nearly all, participants randomized? | | | Y | The authors report data on all patients involved in randomization |
|  | 3.2 If N/PN/NI to 3.1: Is there evidence that result was not biased by missing outcome data? | | | NA |  |
|  | 3.3 If N/PN to 3.2: Could missingness in the outcome depend on its true value? | | | NA |  |
|  | 3.4 If Y/PY/NI to 3.3: Is it likely that missingness in the outcome depended on its true value? | | | NA |  |
|  | **Risk of bias judgement** |  |  | **Low** |  |
| **Bias in measurement of the outcome** | 4.1 Was the method of measuring the outcome inappropriate? | | | N | method is appropriate |
|  | 4.2 Could measurement or ascertainment of the outcome have differed between intervention groups? | | | N | Outcome measures (data collection) involving the same measurement methods and thresholds, used at comparable time points |
|  | 4.3 Were outcome assessors aware of the intervention received by study participants? | | | NI | not enough information |
|  | 4.4 If Y/PY/NI to 4.3: Could assessment of the outcome have been influenced by knowledge of intervention received? | | | N | Results do not involve judgment |
|  | 4.5 If Y/PY/NI to 4.4: Is it likely that assessment of the outcome was influenced by knowledge of intervention received? | | | NA |  |
|  | **Risk of bias judgement** |  |  | **Low** |  |
| **Bias in selection of the reported result** | 5.1 Were the data that produced this result analysed in accordance with a pre-specified analysis plan that was finalized before unblinded outcome data were available for analysis? | | | NI | No mention of pre-specified analyses or protocols |
|  | 5.2 ... multiple eligible outcome measurements (e.g. scales, definitions, time points) within the outcome domain? | | | NI | not enough information |
|  | 5.3 ... multiple eligible analyses of the data? | |  | NI | not enough information |
|  | **Risk of bias judgement** |  |  | **Some concerns** |  |
| **Overall bias** | **Risk of bias judgement** |  |  | **High** |  |
|  |  |  |  |  |  |
|  |  |  |  |  |  |
| **Unique ID** | A27 | **Study ID** | Zhou, 2016 | **Assessor** |  |
| **Ref or Label** |  | **Aim** | assignment to intervention (the 'intention-to-treat' effect) | |  |
| **Experimental** | GZFL+MFP | **Comparator** | MFP | **Source** | Journal article(s) with results of the trial |
| **Outcome** | CER | **Results** |  | **Weight** | 1 |
| **Domain** | **Signalling question** |  |  | **Response** | **Comments** |
| **Bias arising from the randomization process** | 1.1 Was the allocation sequence random? | |  | Y | The author uses the random number table method |
|  | 1.2 Was the allocation sequence concealed until participants were enrolled and assigned to interventions? | | | Y |  |
|  | 1.3 Did baseline differences between intervention groups suggest a problem with the randomization process? | | | N | There is no difference between baseline data |
|  | **Risk of bias judgement** |  |  | **Low** |  |
| **Bias due to deviations from intended interventions** | 2.1.Were participants aware of their assigned intervention during the trial? | | | Y | Does not mention whether blinding is used |
|  | 2.2.Were carers and people delivering the interventions aware of participants' assigned intervention during the trial? | | | Y |  |
|  | 2.3. If Y/PY/NI to 2.1 or 2.2: Were there deviations from the intended intervention that arose because of the experimental context? | | | NI | No relevant information reported |
|  | 2.4 If Y/PY to 2.3: Were these deviations likely to have affected the outcome? | | | NA |  |
|  | 2.5. If Y/PY/NI to 2.4: Were these deviations from intended intervention balanced between groups? | | | NA |  |
|  | 2.6 Was an appropriate analysis used to estimate the effect of assignment to intervention? | | | NI | not enough information |
|  | 2.7 If N/PN/NI to 2.6: Was there potential for a substantial impact (on the result) of the failure to analyse participants in the group to which they were randomized? | | | NI | not enough information |
|  | **Risk of bias judgement** |  |  | **High** |  |
| **Bias due to missing outcome data** | 3.1 Were data for this outcome available for all, or nearly all, participants randomized? | | | Y | The authors report data on all patients involved in randomization |
|  | 3.2 If N/PN/NI to 3.1: Is there evidence that result was not biased by missing outcome data? | | | NA |  |
|  | 3.3 If N/PN to 3.2: Could missingness in the outcome depend on its true value? | | | NA |  |
|  | 3.4 If Y/PY/NI to 3.3: Is it likely that missingness in the outcome depended on its true value? | | | NA |  |
|  | **Risk of bias judgement** |  |  | **Low** |  |
| **Bias in measurement of the outcome** | 4.1 Was the method of measuring the outcome inappropriate? | | | N | method is appropriate |
|  | 4.2 Could measurement or ascertainment of the outcome have differed between intervention groups? | | | N | Outcome measures (data collection) involving the same measurement methods and thresholds, used at comparable time points |
|  | 4.3 Were outcome assessors aware of the intervention received by study participants? | | | NI | not enough information |
|  | 4.4 If Y/PY/NI to 4.3: Could assessment of the outcome have been influenced by knowledge of intervention received? | | | N | Results do not involve judgment |
|  | 4.5 If Y/PY/NI to 4.4: Is it likely that assessment of the outcome was influenced by knowledge of intervention received? | | | NA |  |
|  | **Risk of bias judgement** |  |  | **Low** |  |
| **Bias in selection of the reported result** | 5.1 Were the data that produced this result analysed in accordance with a pre-specified analysis plan that was finalized before unblinded outcome data were available for analysis? | | | NI | No mention of pre-specified analyses or protocols |
|  | 5.2 ... multiple eligible outcome measurements (e.g. scales, definitions, time points) within the outcome domain? | | | NI | not enough information |
|  | 5.3 ... multiple eligible analyses of the data? | |  | NI | not enough information |
|  | **Risk of bias judgement** |  |  | **Some concerns** |  |
| **Overall bias** | **Risk of bias judgement** |  |  | **High** |  |
|  |  |  |  |  |  |
|  |  |  |  |  |  |
| **Unique ID** | A28 | **Study ID** | Wang, 2004 | **Assessor** |  |
| **Ref or Label** |  | **Aim** | assignment to intervention (the 'intention-to-treat' effect) | |  |
| **Experimental** | GZFL+MFP | **Comparator** | MFP | **Source** | Journal article(s) with results of the trial |
| **Outcome** | CER | **Results** |  | **Weight** | 1 |
| **Domain** | **Signalling question** |  |  | **Response** | **Comments** |
| **Bias arising from the randomization process** | 1.1 Was the allocation sequence random? | |  | NI | The authors did not describe in detail the generation and allocation concealment of random sequences |
|  | 1.2 Was the allocation sequence concealed until participants were enrolled and assigned to interventions? | | | NI |  |
|  | 1.3 Did baseline differences between intervention groups suggest a problem with the randomization process? | | | N | There is no difference between baseline data |
|  | **Risk of bias judgement** |  |  | **Some concerns** |  |
| **Bias due to deviations from intended interventions** | 2.1.Were participants aware of their assigned intervention during the trial? | | | Y | Does not mention whether blinding is used |
|  | 2.2.Were carers and people delivering the interventions aware of participants' assigned intervention during the trial? | | | Y |  |
|  | 2.3. If Y/PY/NI to 2.1 or 2.2: Were there deviations from the intended intervention that arose because of the experimental context? | | | NI | No relevant information reported |
|  | 2.4 If Y/PY to 2.3: Were these deviations likely to have affected the outcome? | | | NA |  |
|  | 2.5. If Y/PY/NI to 2.4: Were these deviations from intended intervention balanced between groups? | | | NA |  |
|  | 2.6 Was an appropriate analysis used to estimate the effect of assignment to intervention? | | | NI | not enough information |
|  | 2.7 If N/PN/NI to 2.6: Was there potential for a substantial impact (on the result) of the failure to analyse participants in the group to which they were randomized? | | | NI | not enough information |
|  | **Risk of bias judgement** |  |  | **High** |  |
| **Bias due to missing outcome data** | 3.1 Were data for this outcome available for all, or nearly all, participants randomized? | | | Y | The authors report data on all patients involved in randomization |
|  | 3.2 If N/PN/NI to 3.1: Is there evidence that result was not biased by missing outcome data? | | | NA |  |
|  | 3.3 If N/PN to 3.2: Could missingness in the outcome depend on its true value? | | | NA |  |
|  | 3.4 If Y/PY/NI to 3.3: Is it likely that missingness in the outcome depended on its true value? | | | NA |  |
|  | **Risk of bias judgement** |  |  | **Low** |  |
| **Bias in measurement of the outcome** | 4.1 Was the method of measuring the outcome inappropriate? | | | N | method is appropriate |
|  | 4.2 Could measurement or ascertainment of the outcome have differed between intervention groups? | | | N | Outcome measures (data collection) involving the same measurement methods and thresholds, used at comparable time points |
|  | 4.3 Were outcome assessors aware of the intervention received by study participants? | | | NI | not enough information |
|  | 4.4 If Y/PY/NI to 4.3: Could assessment of the outcome have been influenced by knowledge of intervention received? | | | N | Results do not involve judgment |
|  | 4.5 If Y/PY/NI to 4.4: Is it likely that assessment of the outcome was influenced by knowledge of intervention received? | | | NA |  |
|  | **Risk of bias judgement** |  |  | **Low** |  |
| **Bias in selection of the reported result** | 5.1 Were the data that produced this result analysed in accordance with a pre-specified analysis plan that was finalized before unblinded outcome data were available for analysis? | | | NI | No mention of pre-specified analyses or protocols |
|  | 5.2 ... multiple eligible outcome measurements (e.g. scales, definitions, time points) within the outcome domain? | | | NI | not enough information |
|  | 5.3 ... multiple eligible analyses of the data? | |  | NI | not enough information |
|  | **Risk of bias judgement** |  |  | **Some concerns** |  |
| **Overall bias** | **Risk of bias judgement** |  |  | **High** |  |
