# Supplementary material for: The efficacy and safety of Chinese herbal medicine Guizhi Fuling capsule combined with low dose mifepristone in the treatment of uterine fibroids: a systematic review and meta-analysis of 28 randomized controlled trials
Source: BMC Complement Med Ther. 2023 Feb 18;23:54. doi: 10.1186/s12906-023-03842-y (PMC9938629; doi:10.1186/s12906-023-03842-y)
Supplement: Supplementary file 2 — Additional file 2: Supplementary File S6. Results of subgroup analyses, sensitivity analyses and publication bias. [file 12906_2023_3842_MOESM2_ESM.docx]

**Supplementary File S6. Results of subgroup analyses, sensitivity analyses and publication bias**

**6.1 Sensitivity analyses of the CER.**

**
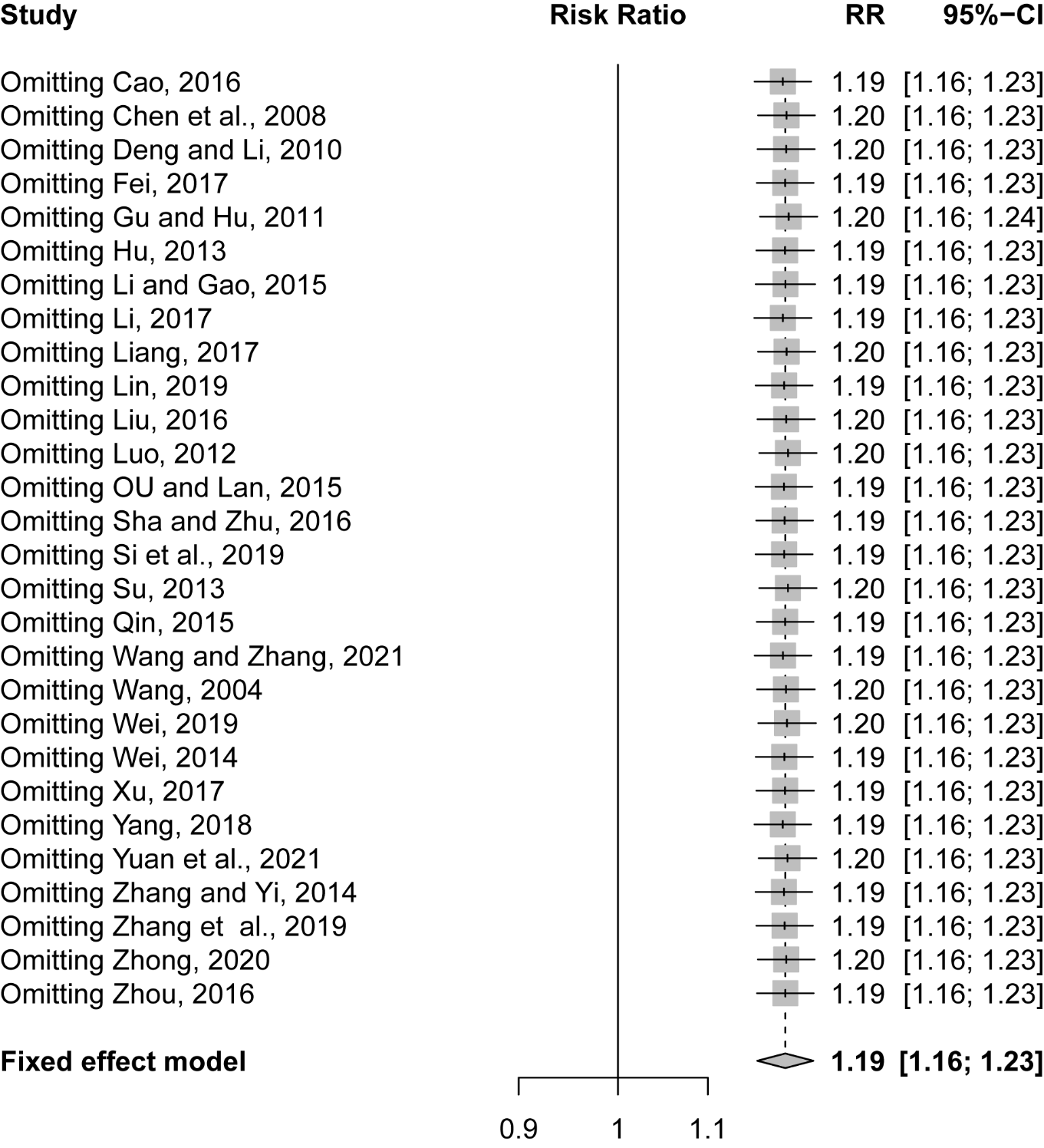
**

**6.2 Subgroup analysis of the CER according to the GZFL does.**

**
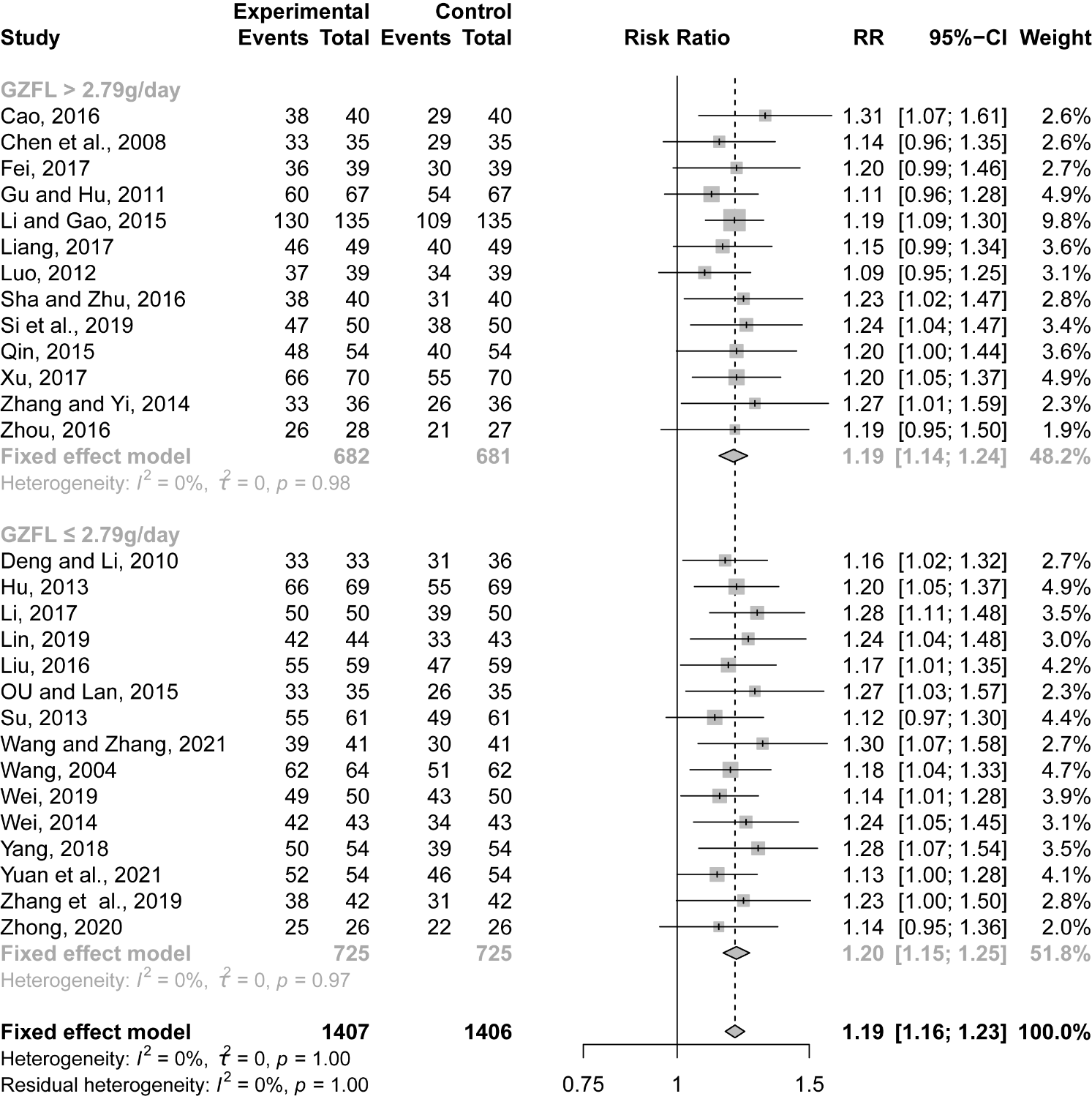
**

**6.3 Subgroup analysis of the CER according to the MFP does.**

**
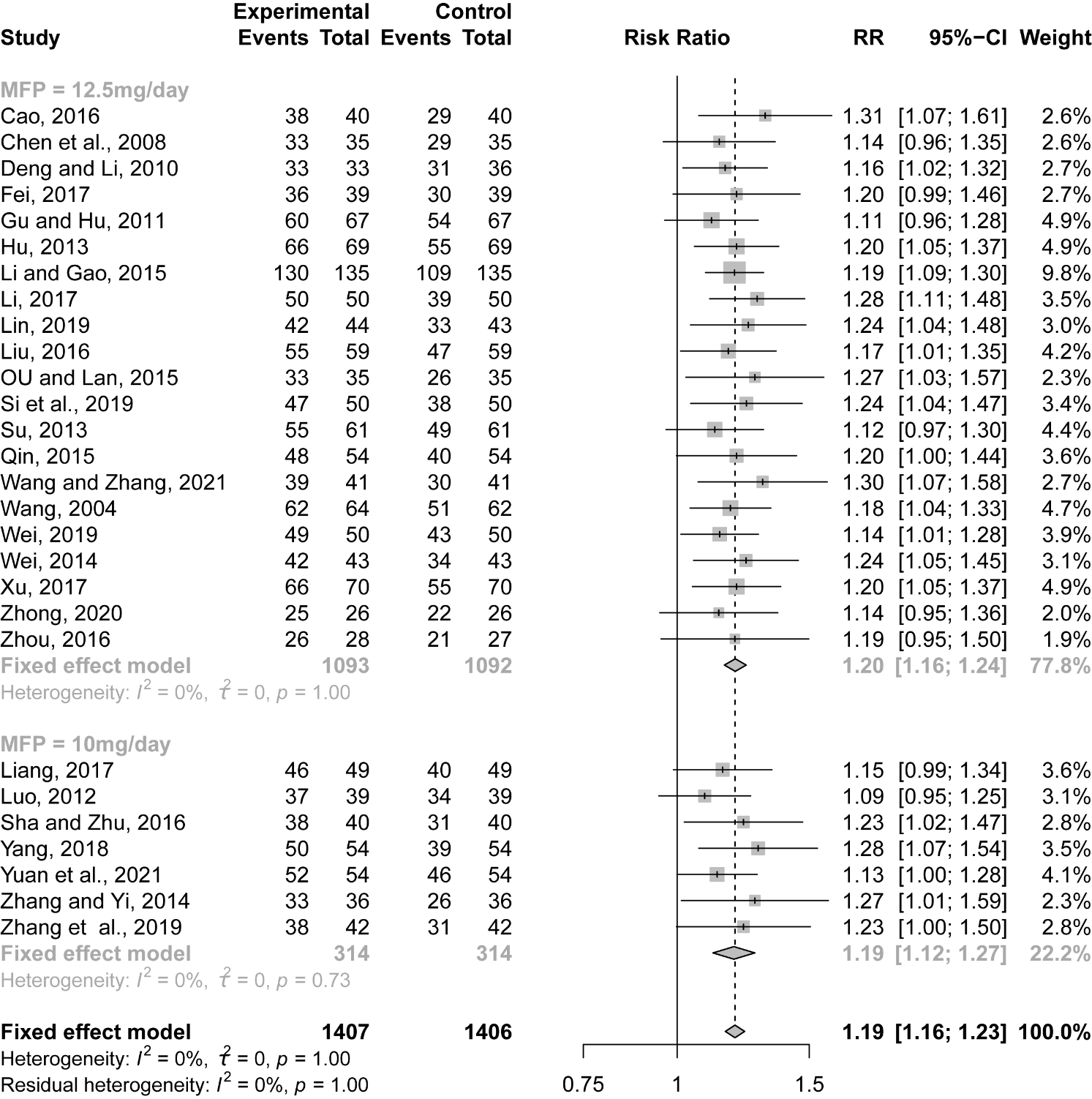
**

**6.4 Subgroup analysis of the CER according to the treatment duration.**

**
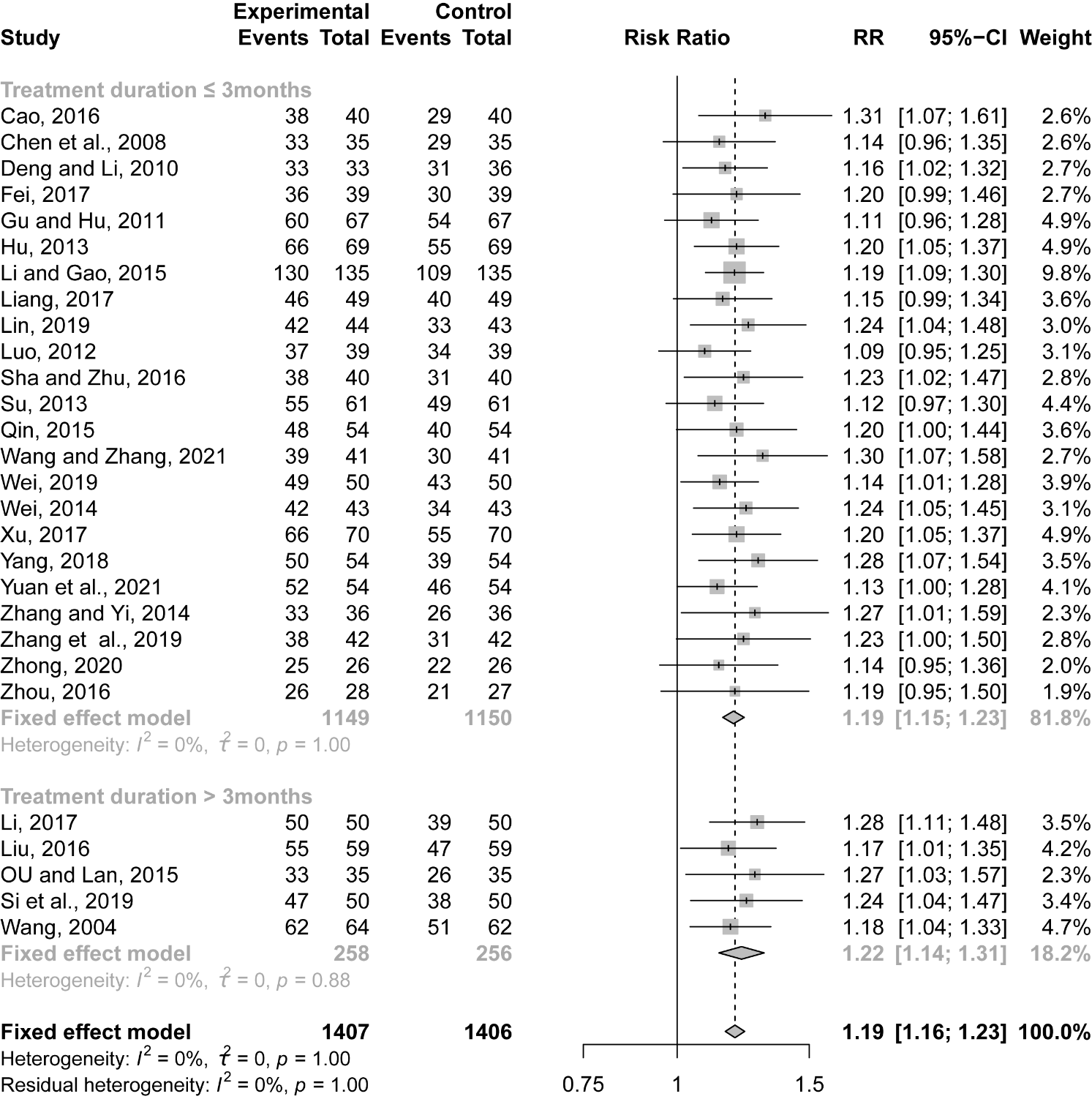
**

**6.5 Subgroup analysis of the CER according to the age.**

**
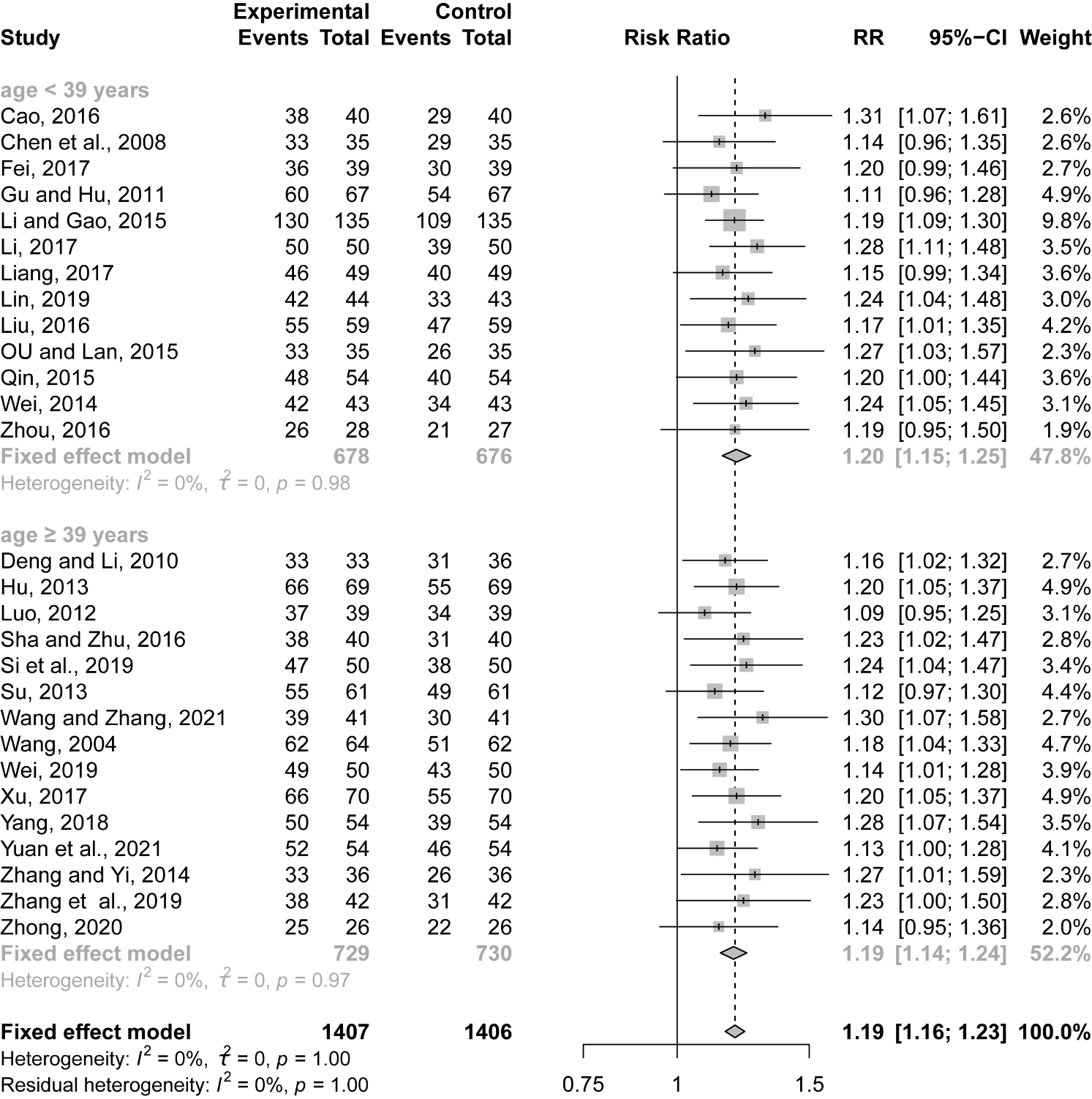
**

**6.6 Sensitivity analyses of the UFV.**

**
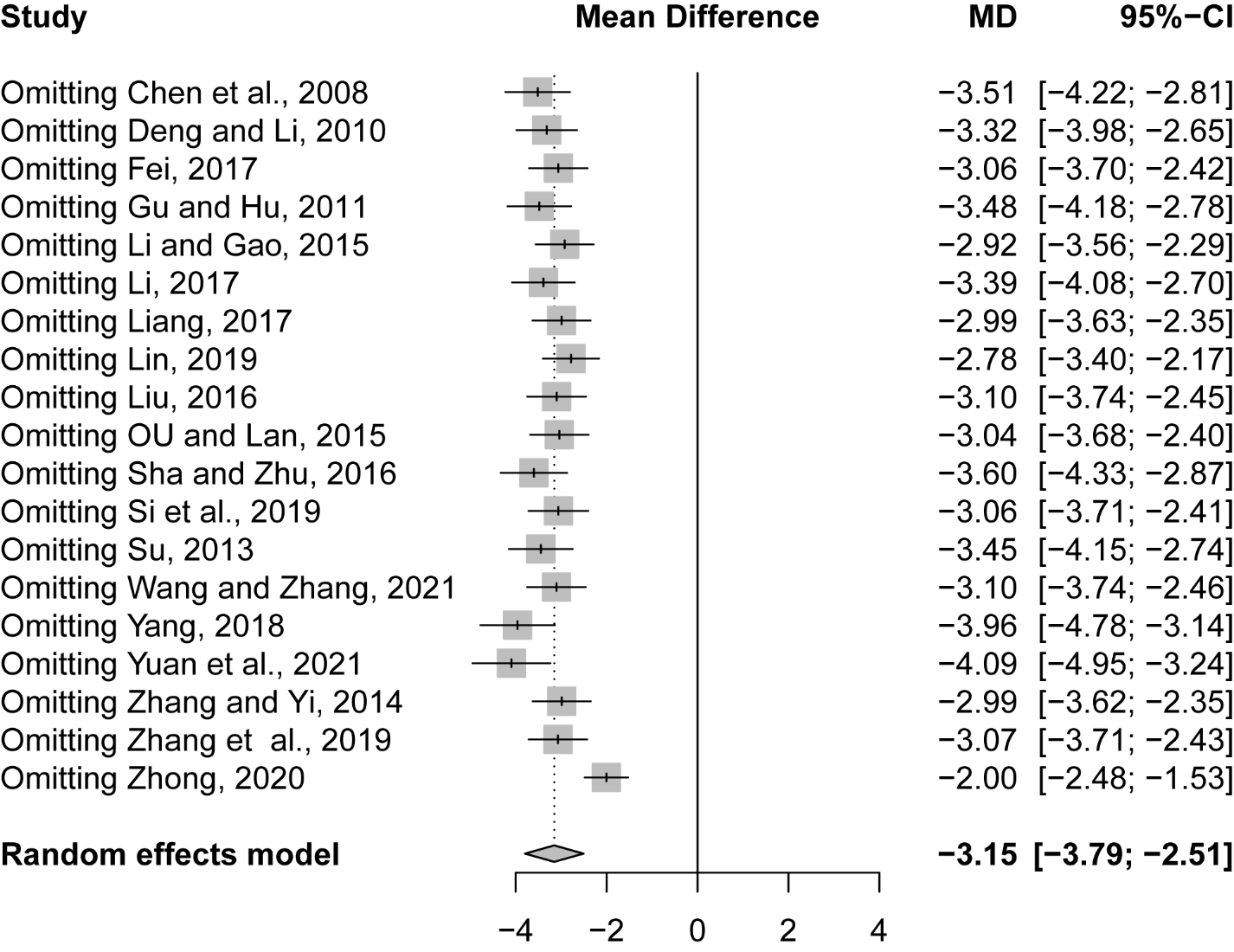
**

**6.7 Subgroup analysis of the UFV according to the GZFL does.**

**
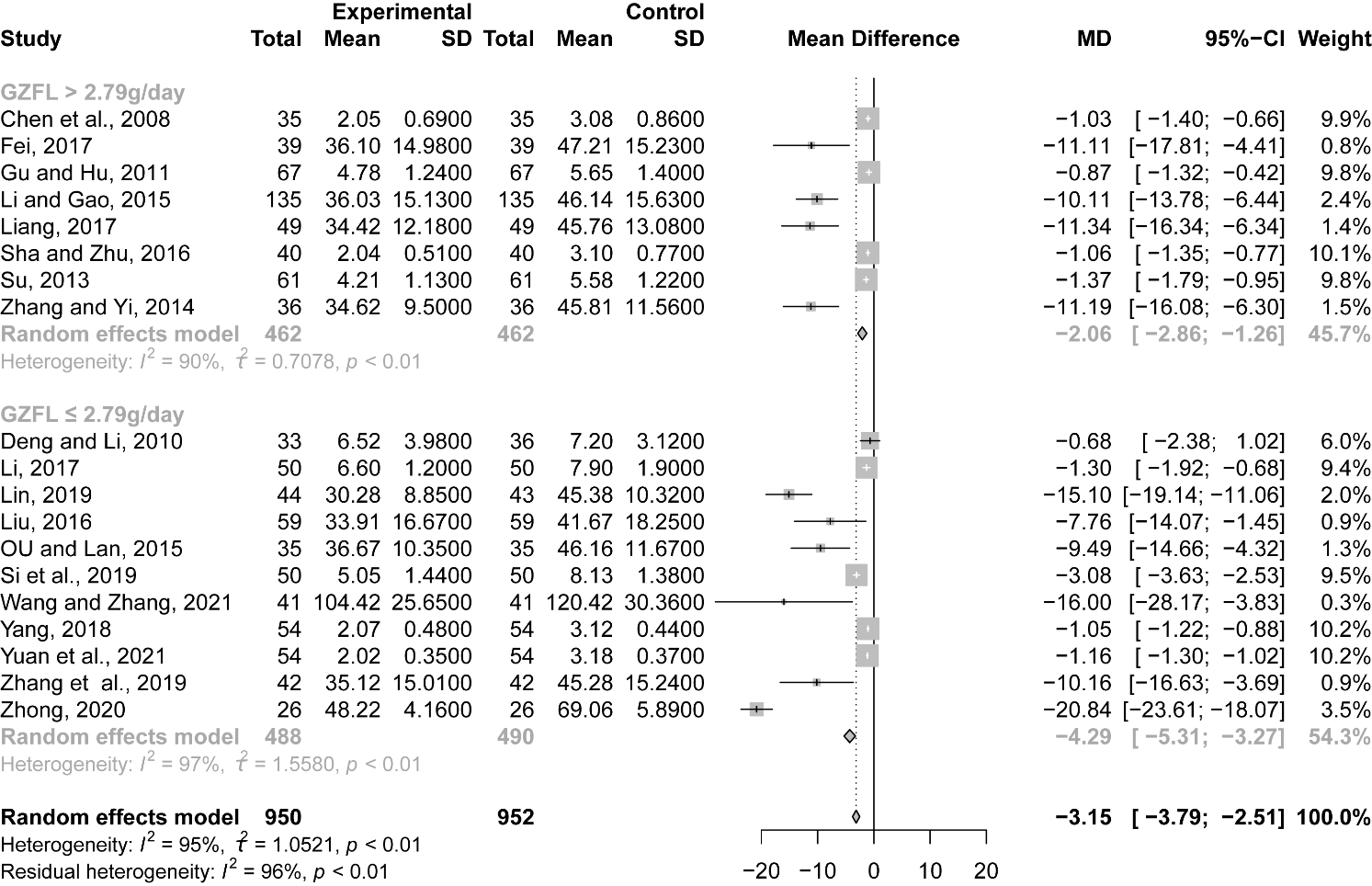
**

**6.8 Subgroup analysis of the UFV according to the MFP does.**

**
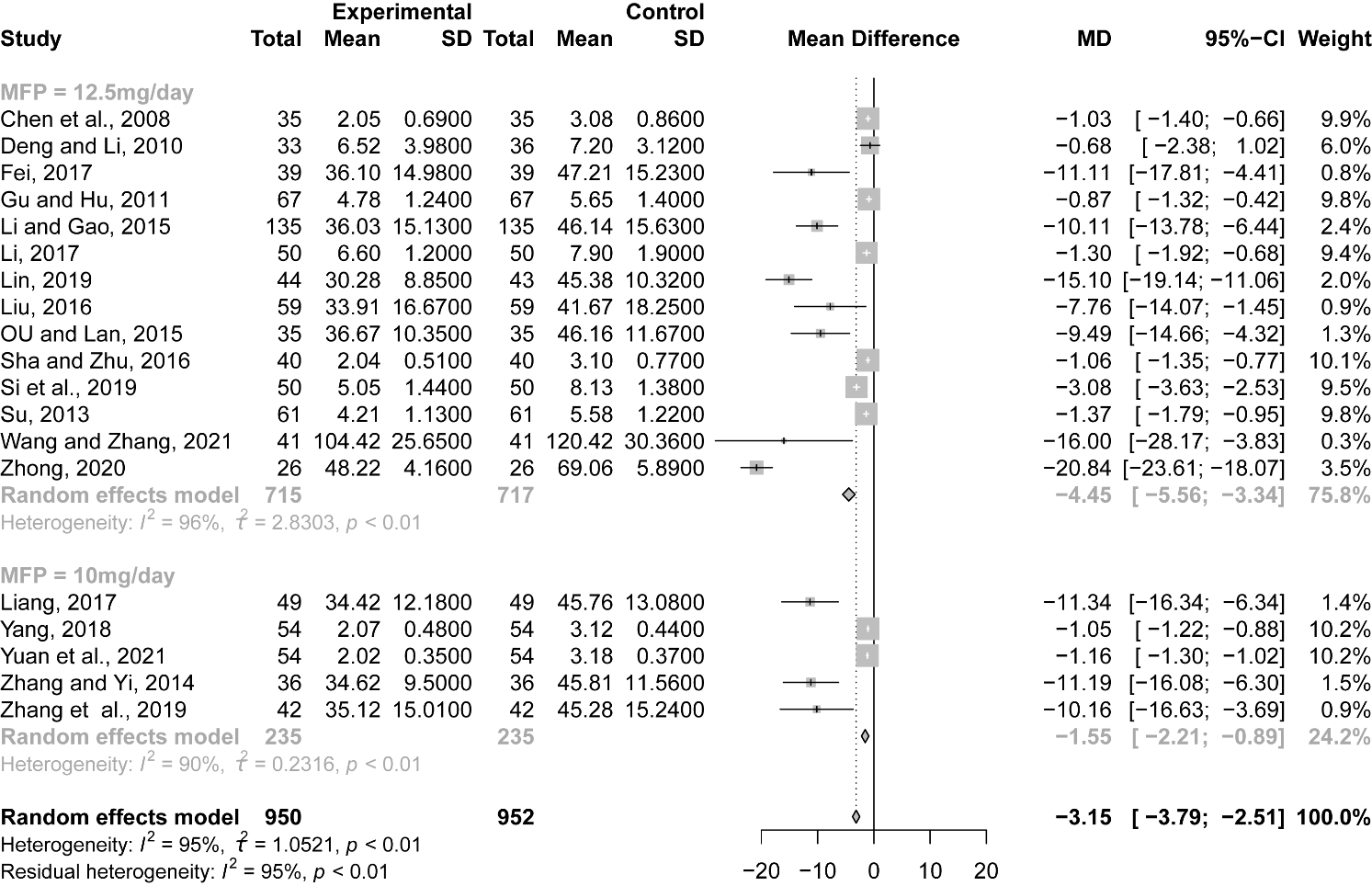
**

**6.9 Subgroup analysis of the UFV according to the treatment duration.**

**
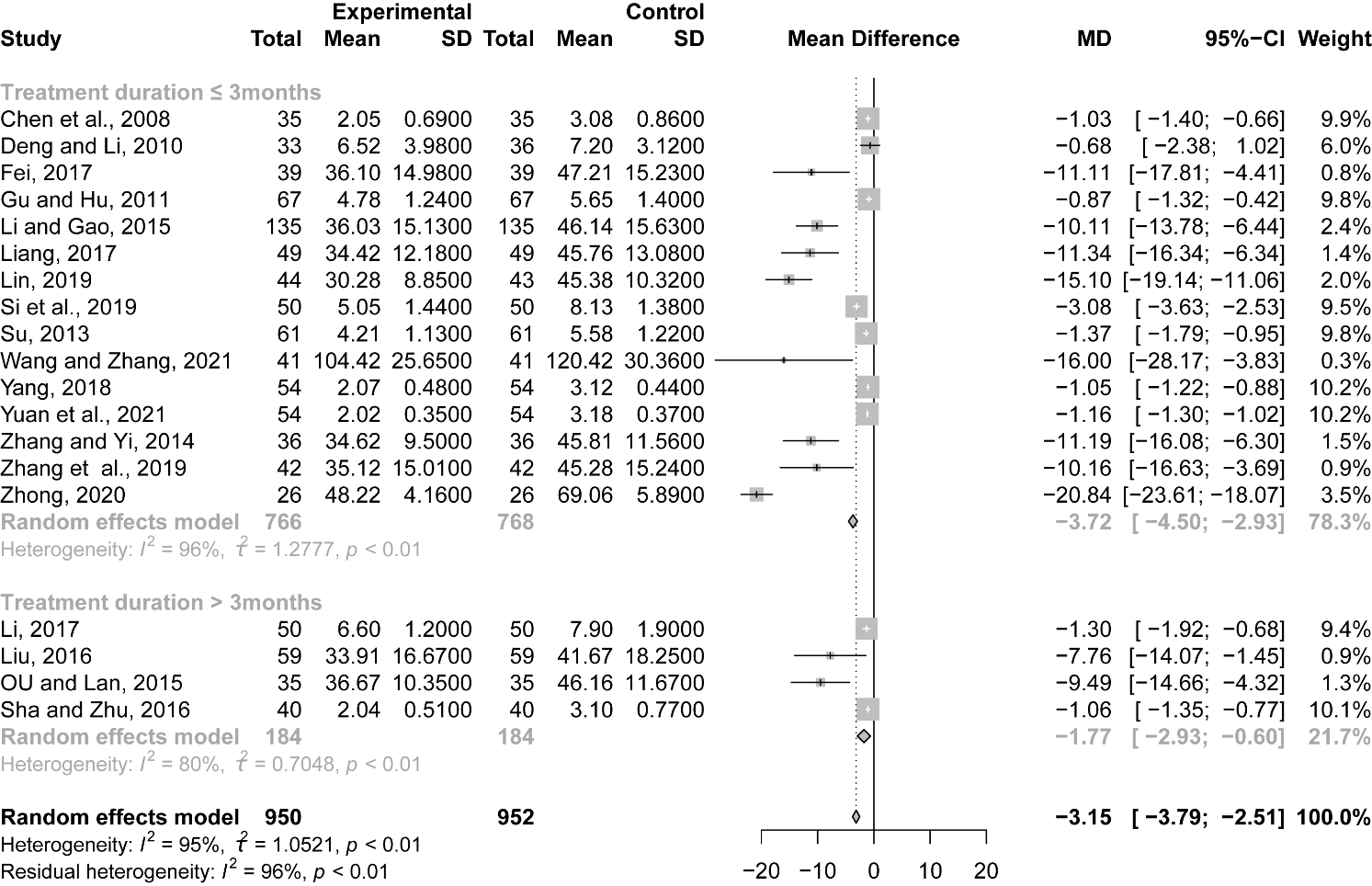
**

**6.10 Subgroup analysis of the UFV according to the age.**

**
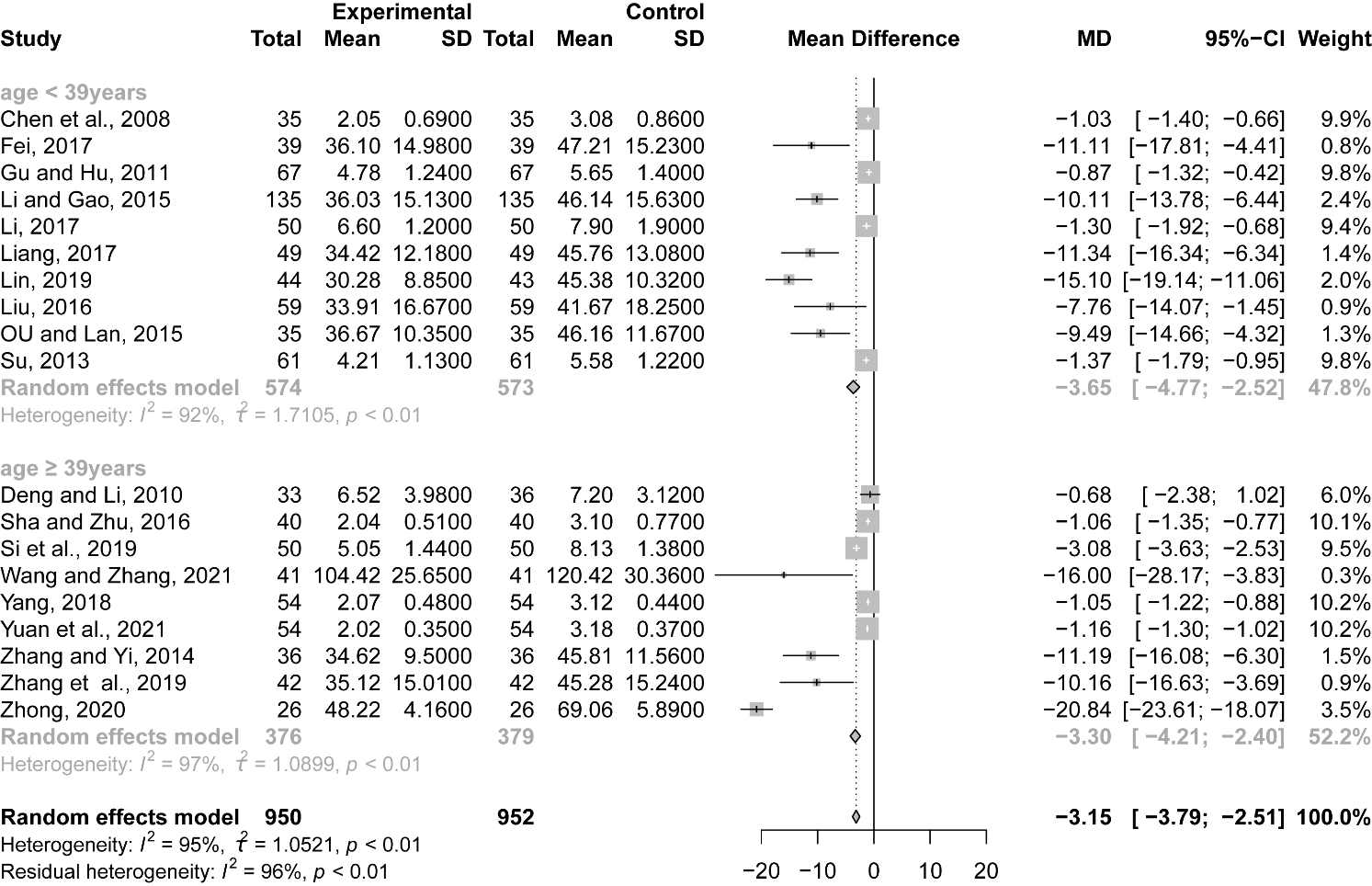
**

**6.11 Sensitivity analyses of the UV.**

**
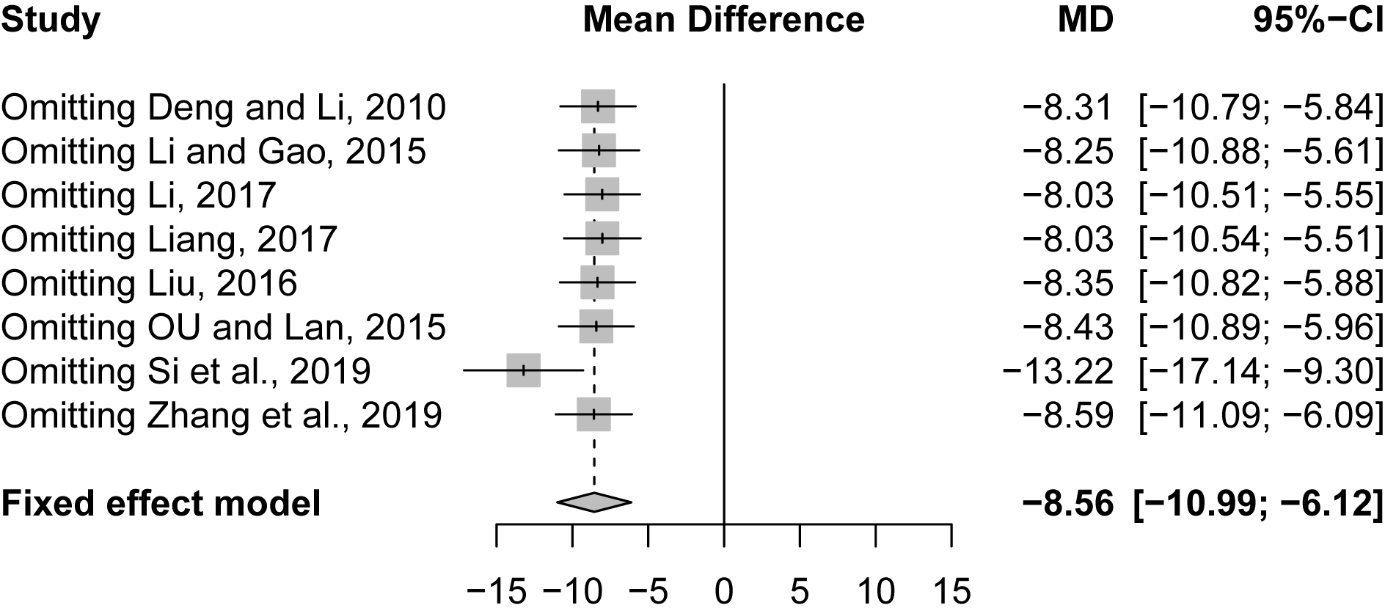
**

**6.12 Subgroup analysis of the UV according to the GZFL does.**

**
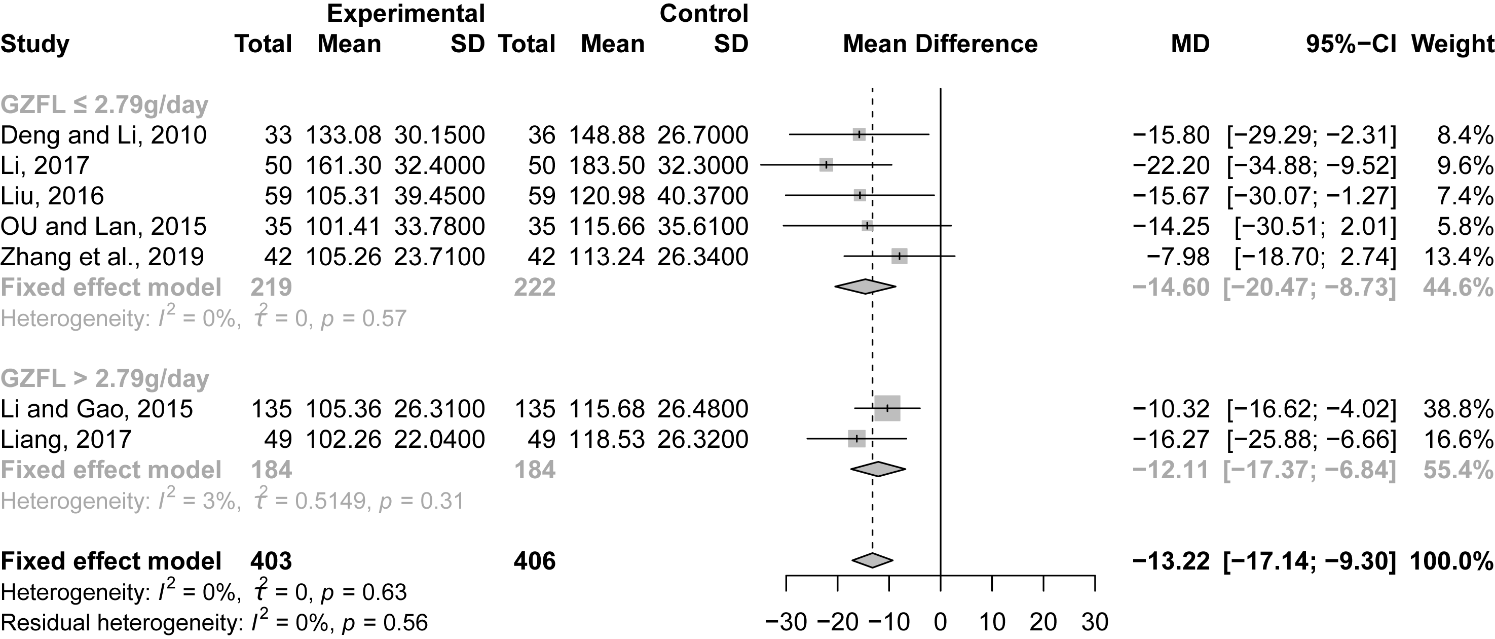
**

**6.13 Subgroup analysis of the UV according to the MFP does.**

**
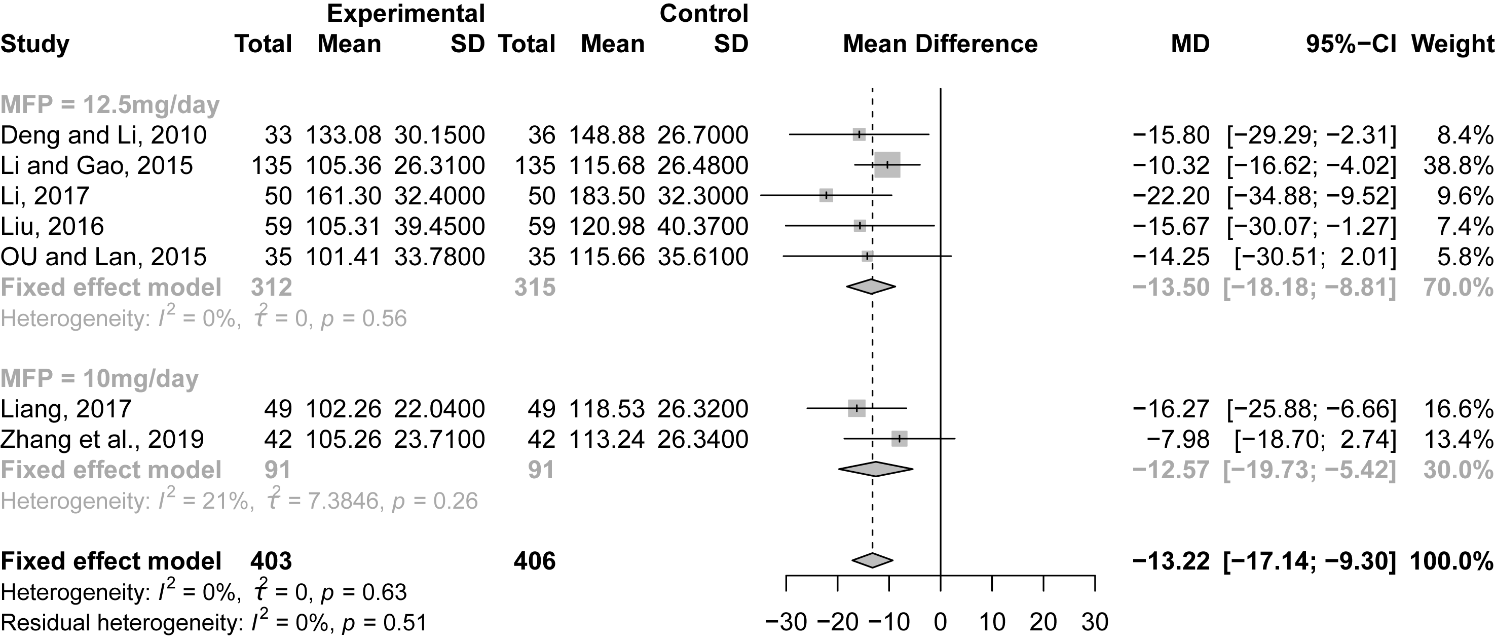
**

**6.14 Subgroup analysis of the UV according to the treatment duration.**

**
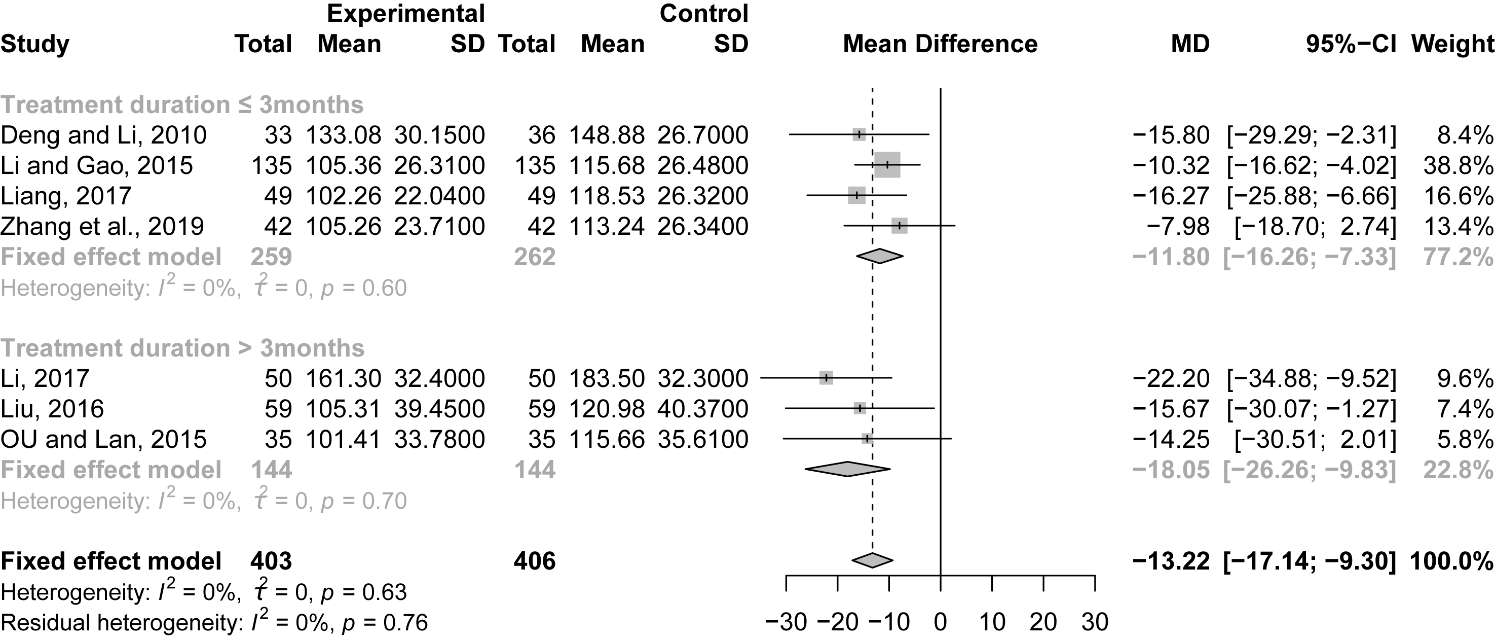
**

**6.15 Subgroup analysis of the UV according to the age.**

**
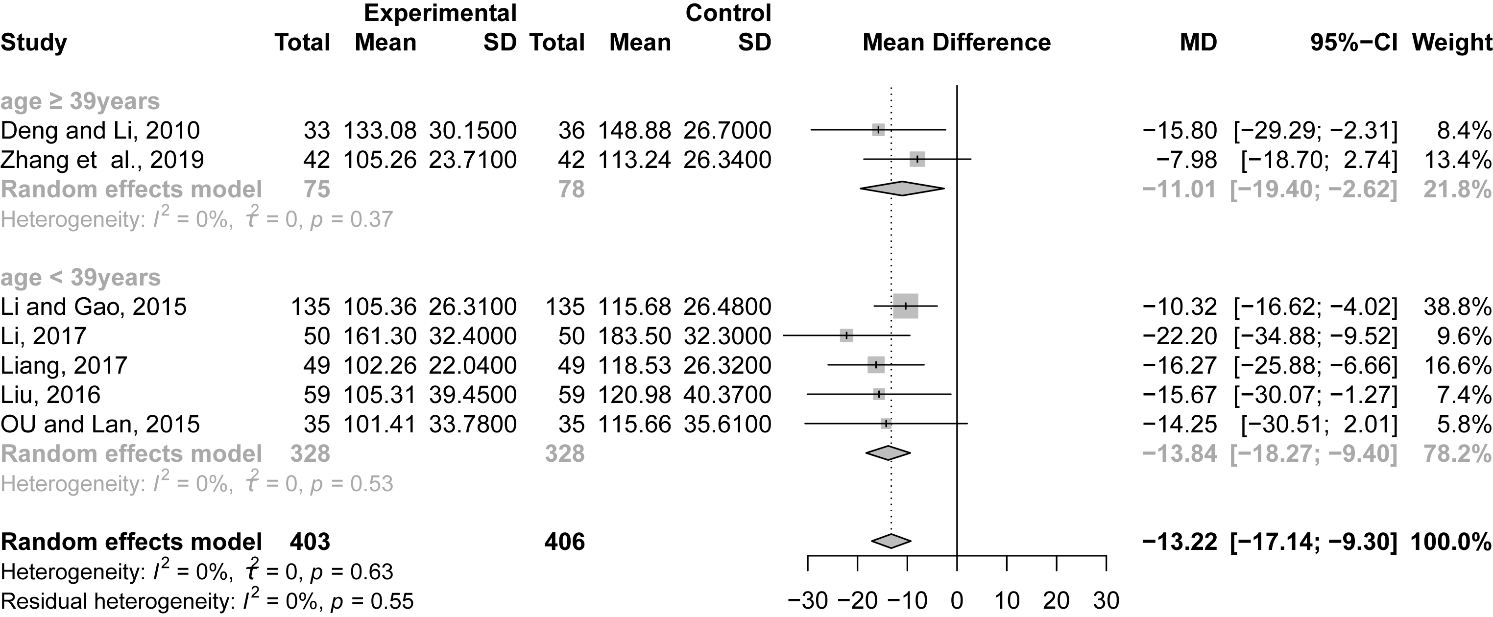
**

**6.16 Sensitivity analyses of the ADR.**

**
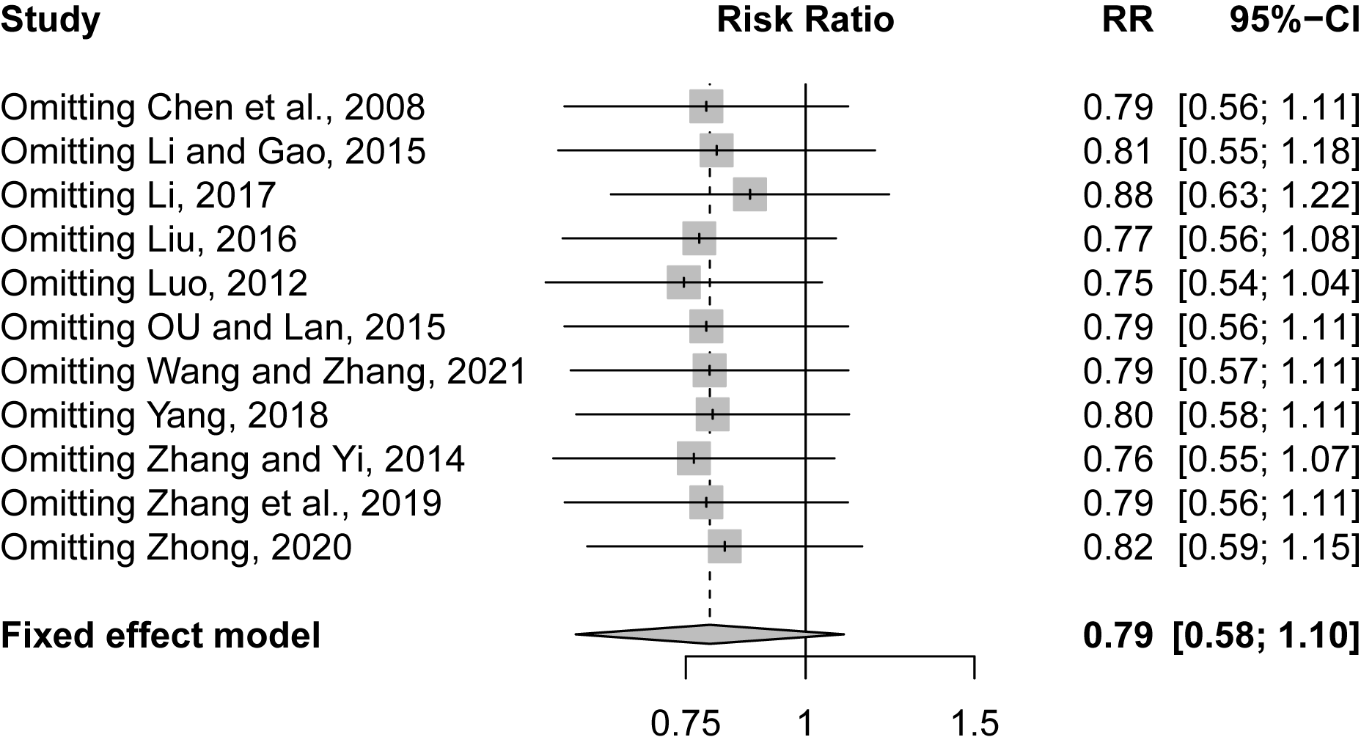
**

**6.17 Subgroup analysis of the ADR according to the GZFL does.**

**
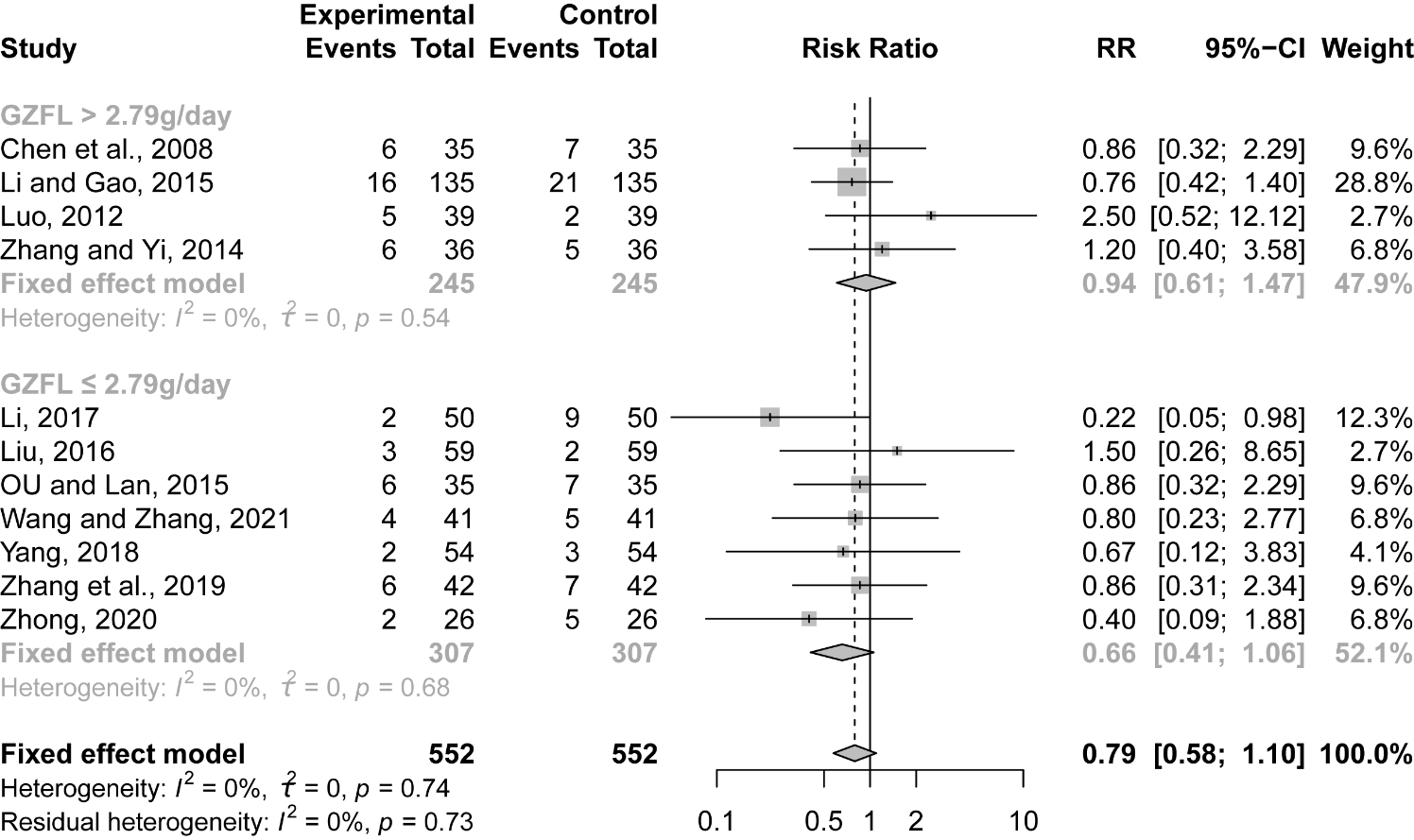
**

**6.18 Subgroup analysis of the ADR according to the MFP does.**

**
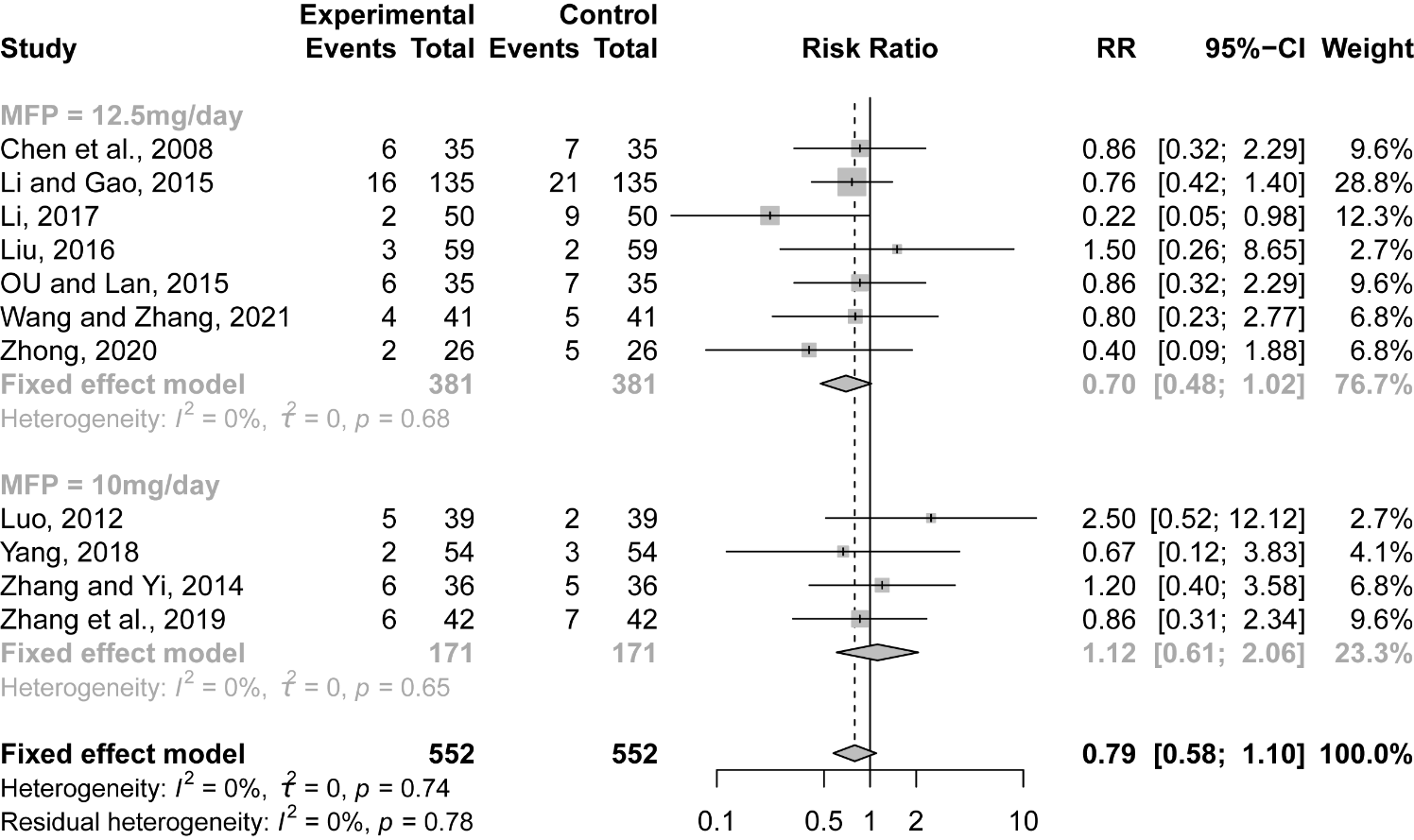
**

**6.19 Subgroup analysis of the ADR according to the treatment duration.**

**
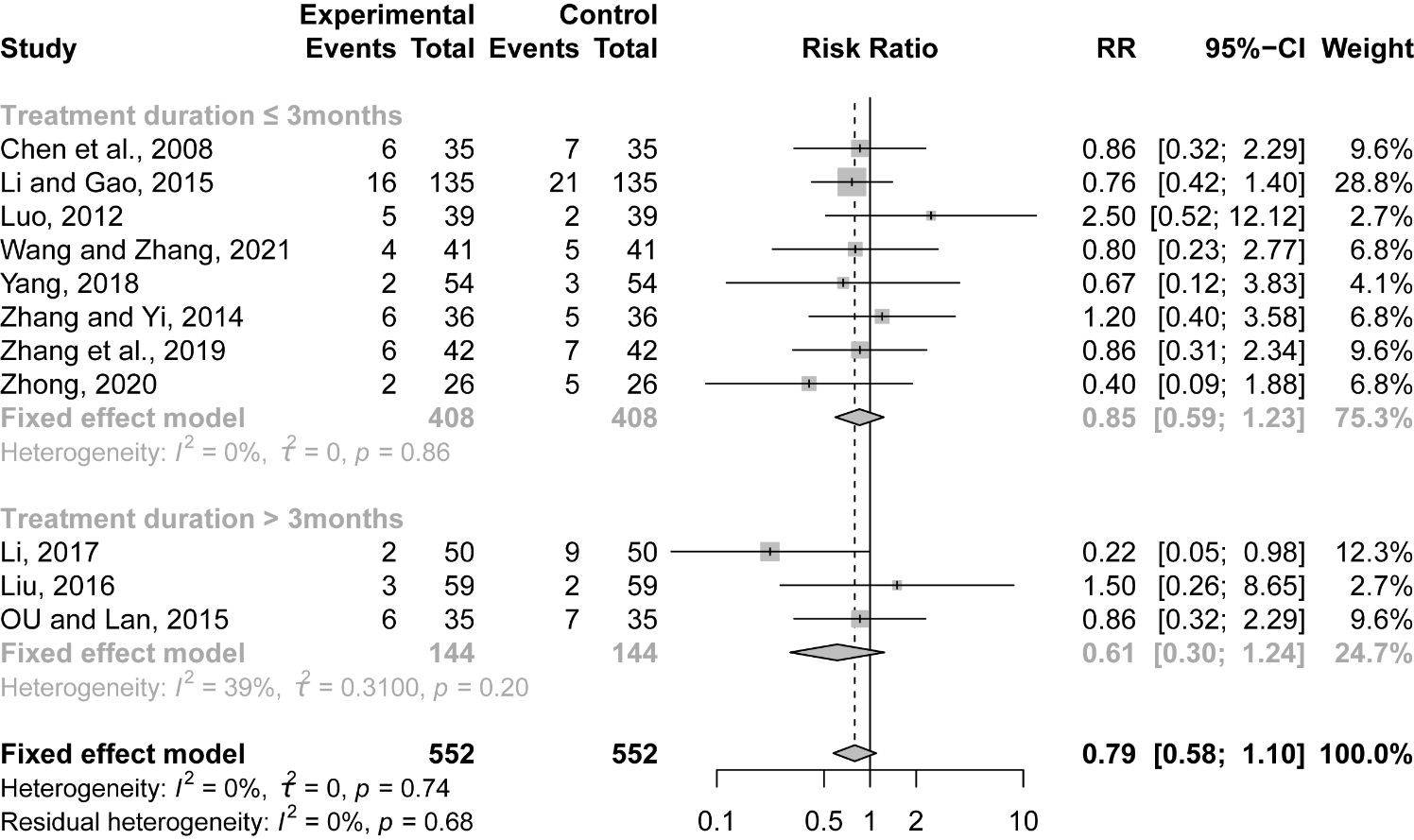
**

**6.20 Subgroup analysis of the ADR according to the age.**

**
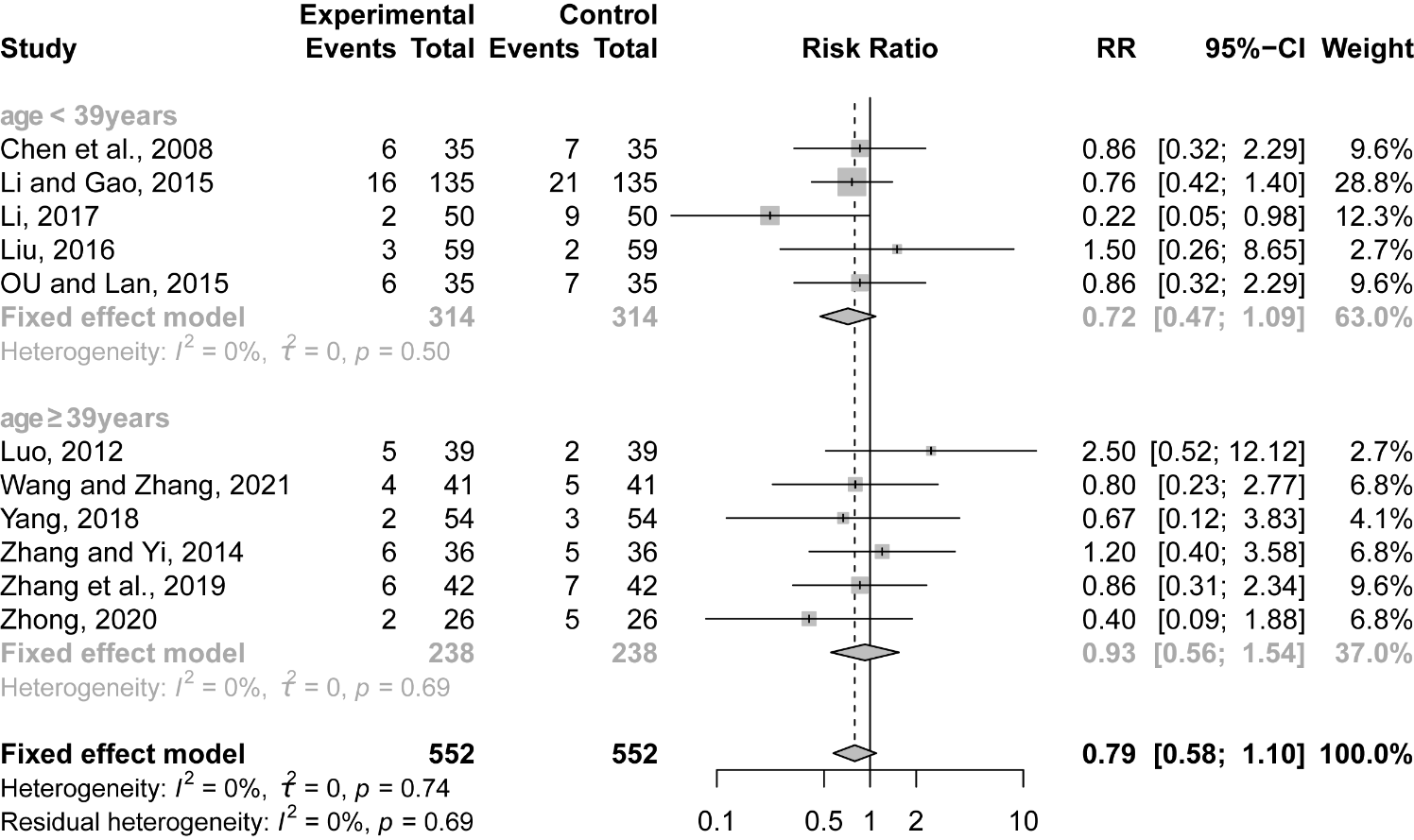
**

**6.21Sensitivity analyses of the FSH.**

**
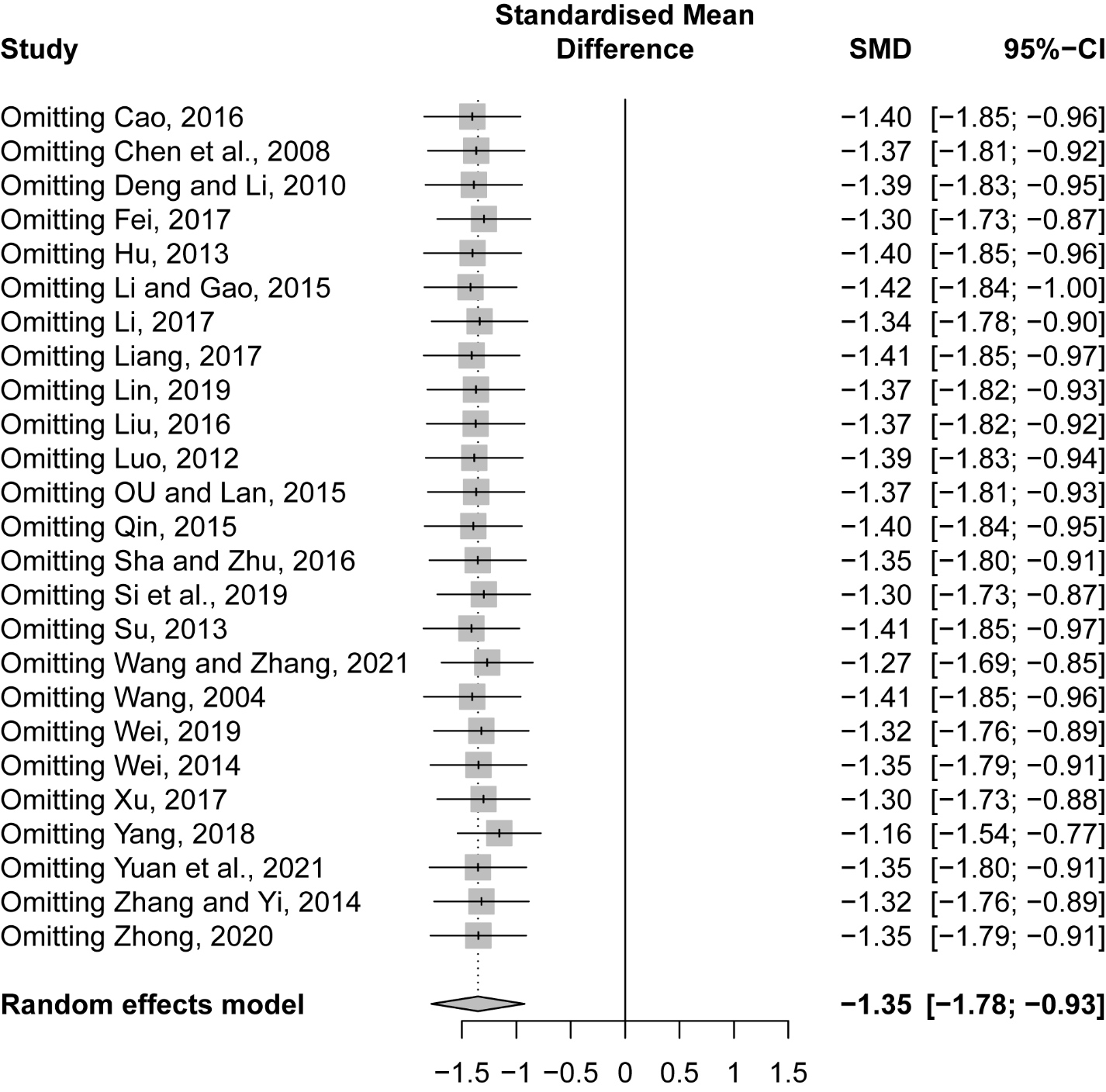
**

**6.22 Subgroup analysis of the FSH according to the GZFL does.**

**
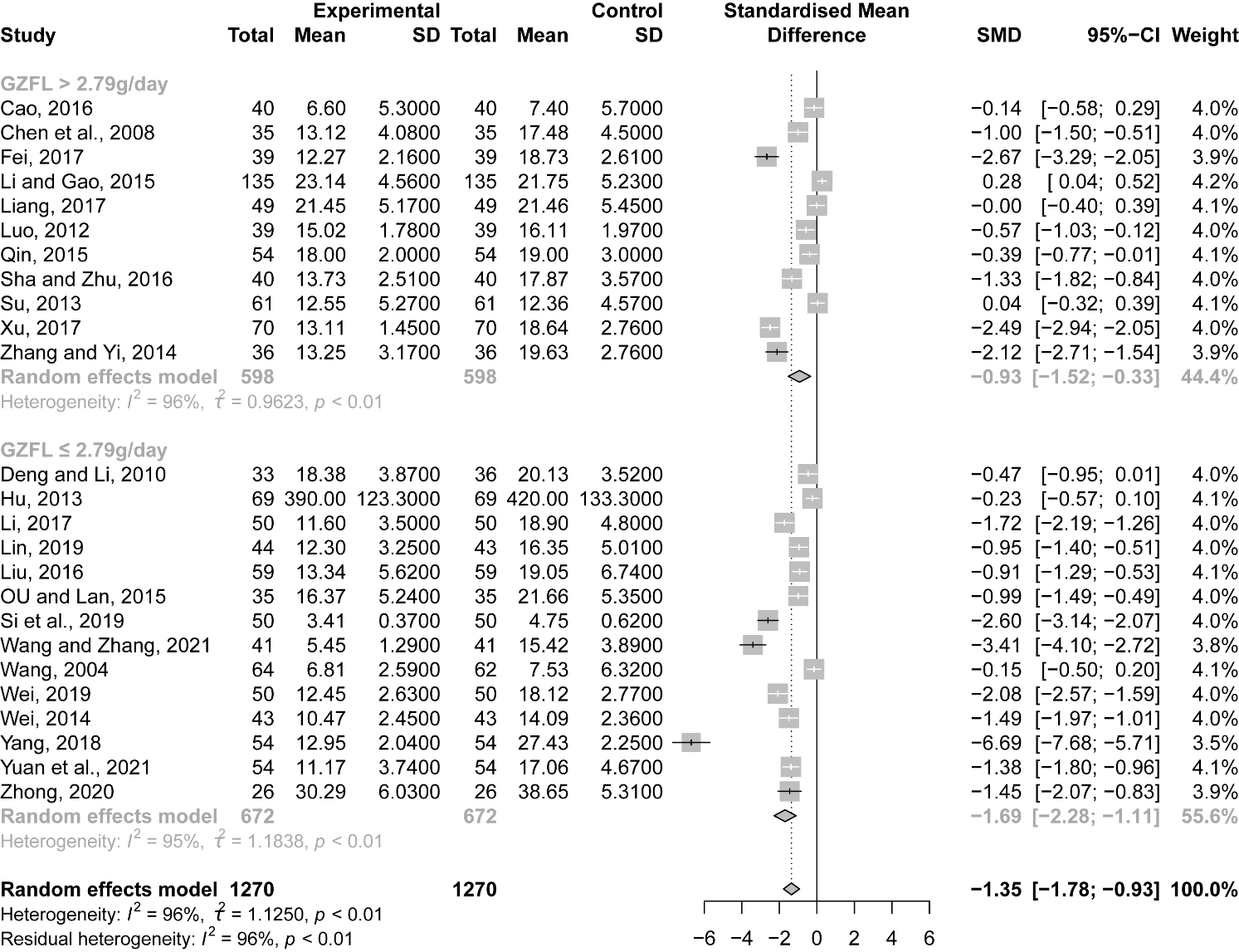
**

**6.23 Subgroup analysis of the FSH according to the MFP does.**

**
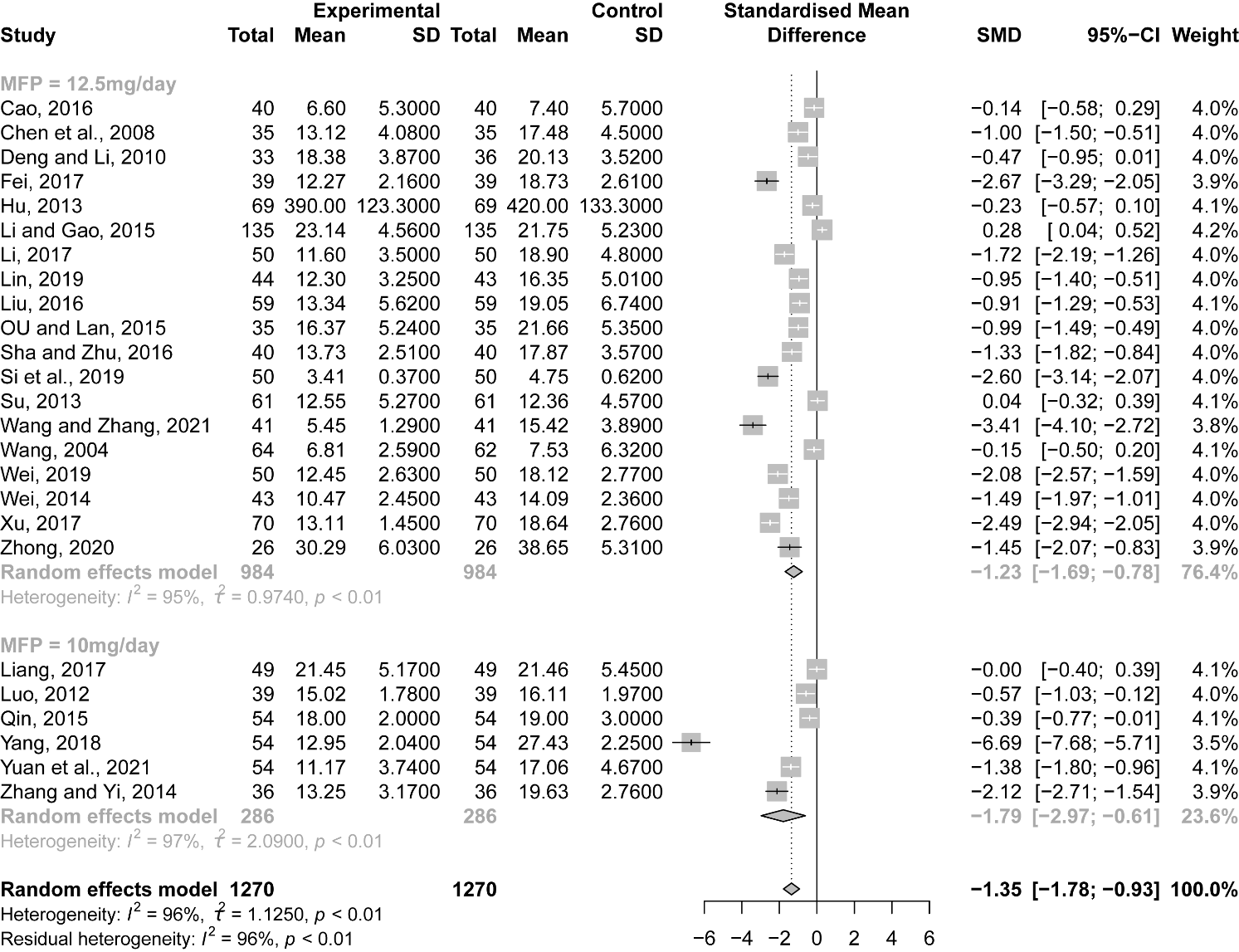
**

**6.24 Subgroup analysis of the FSH according to the treatment duration.**

**
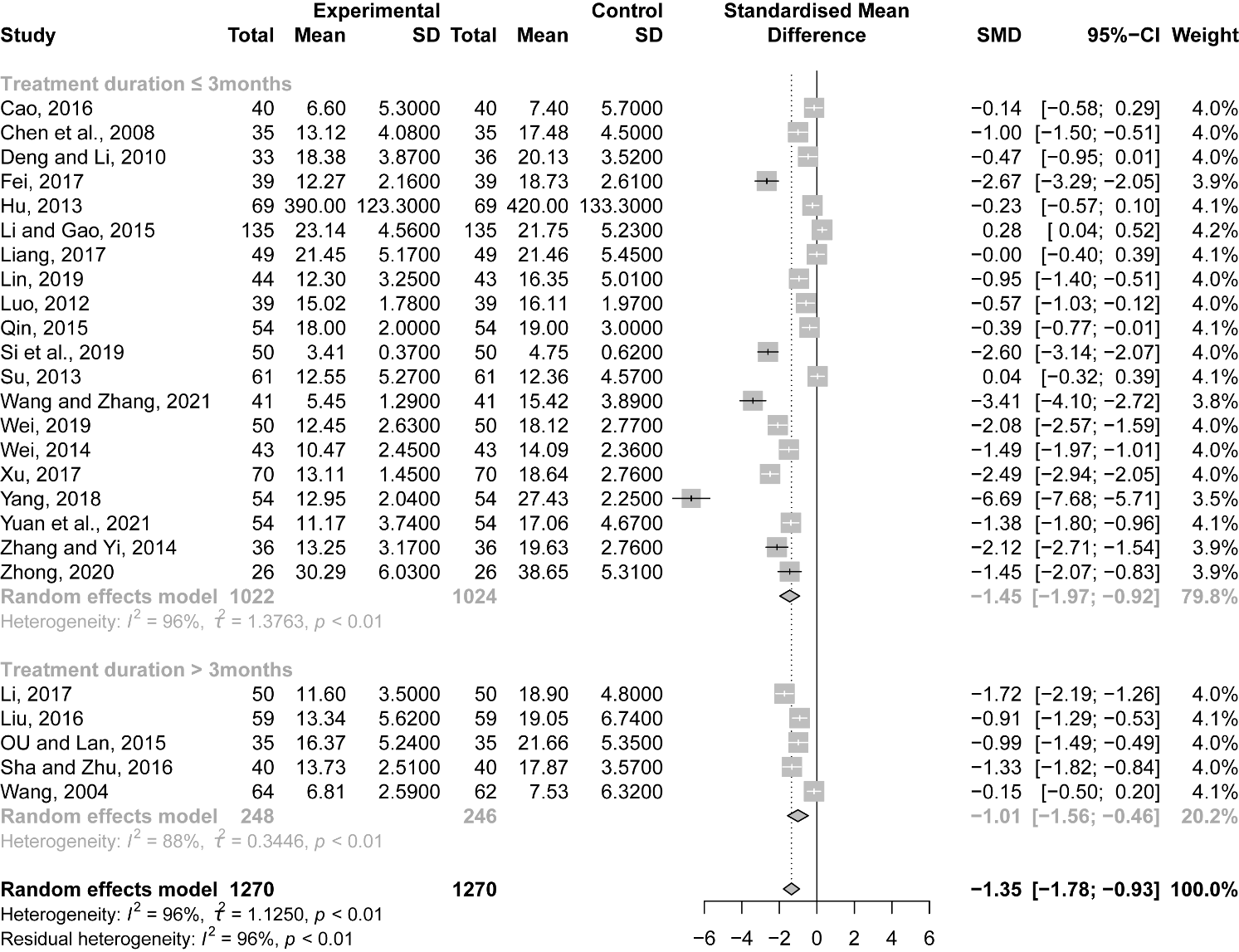
**

**6.25 Subgroup analysis of the FSH according to the age.**

**
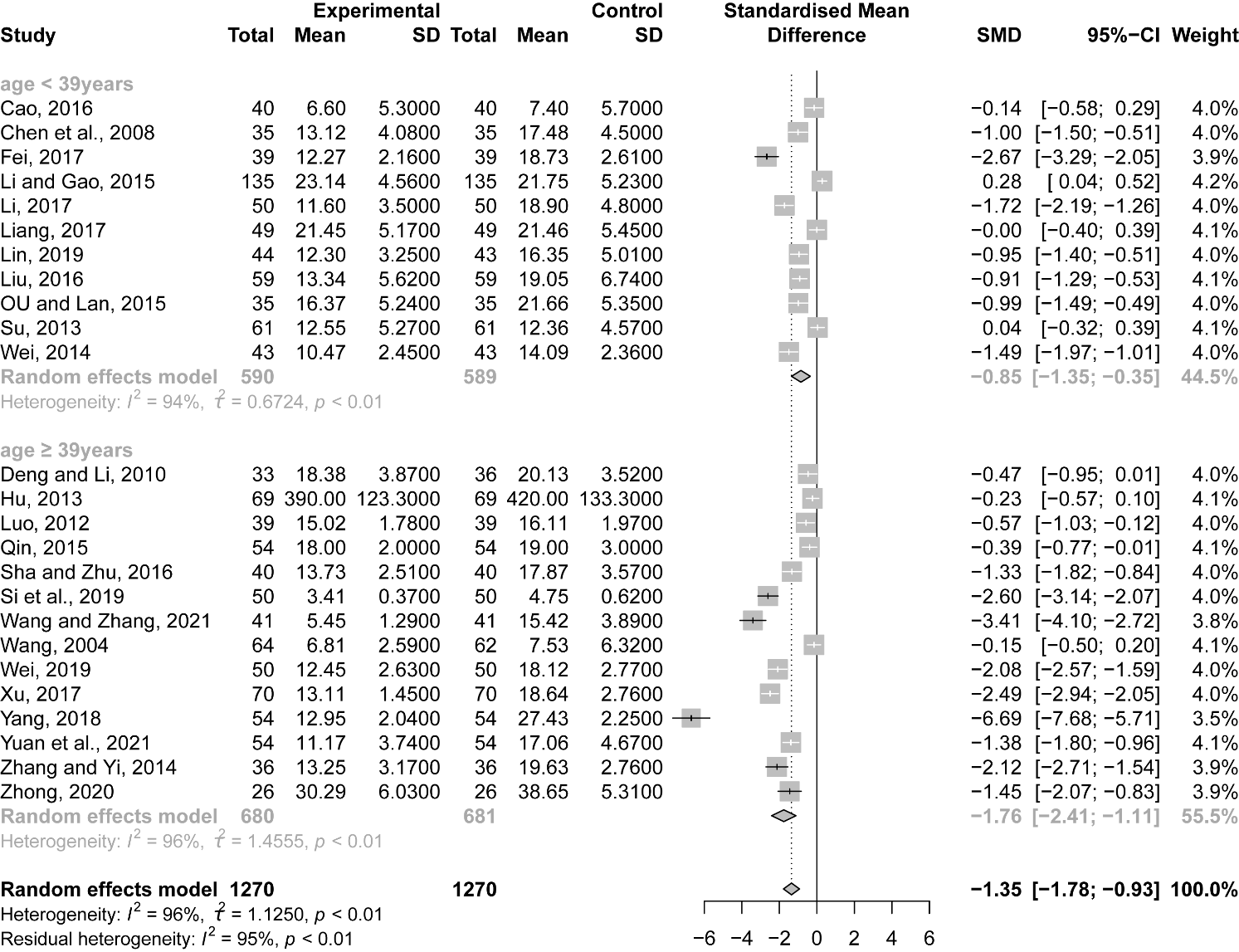
**

**6.26Sensitivity analyses of the E_2_.**

**
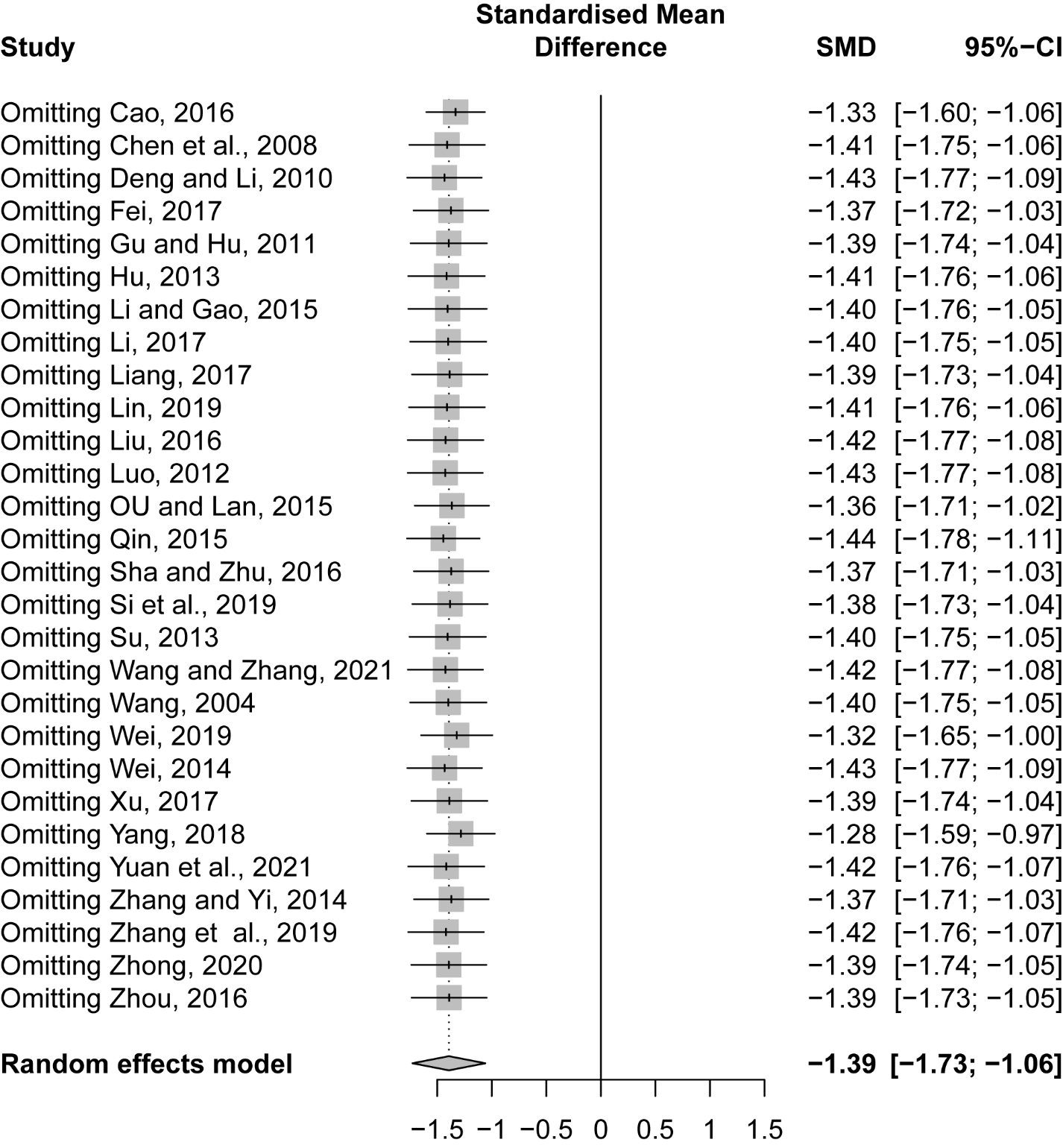
**

**6.27 Subgroup analysis of the E_2_ according to the GZFL does.**

**
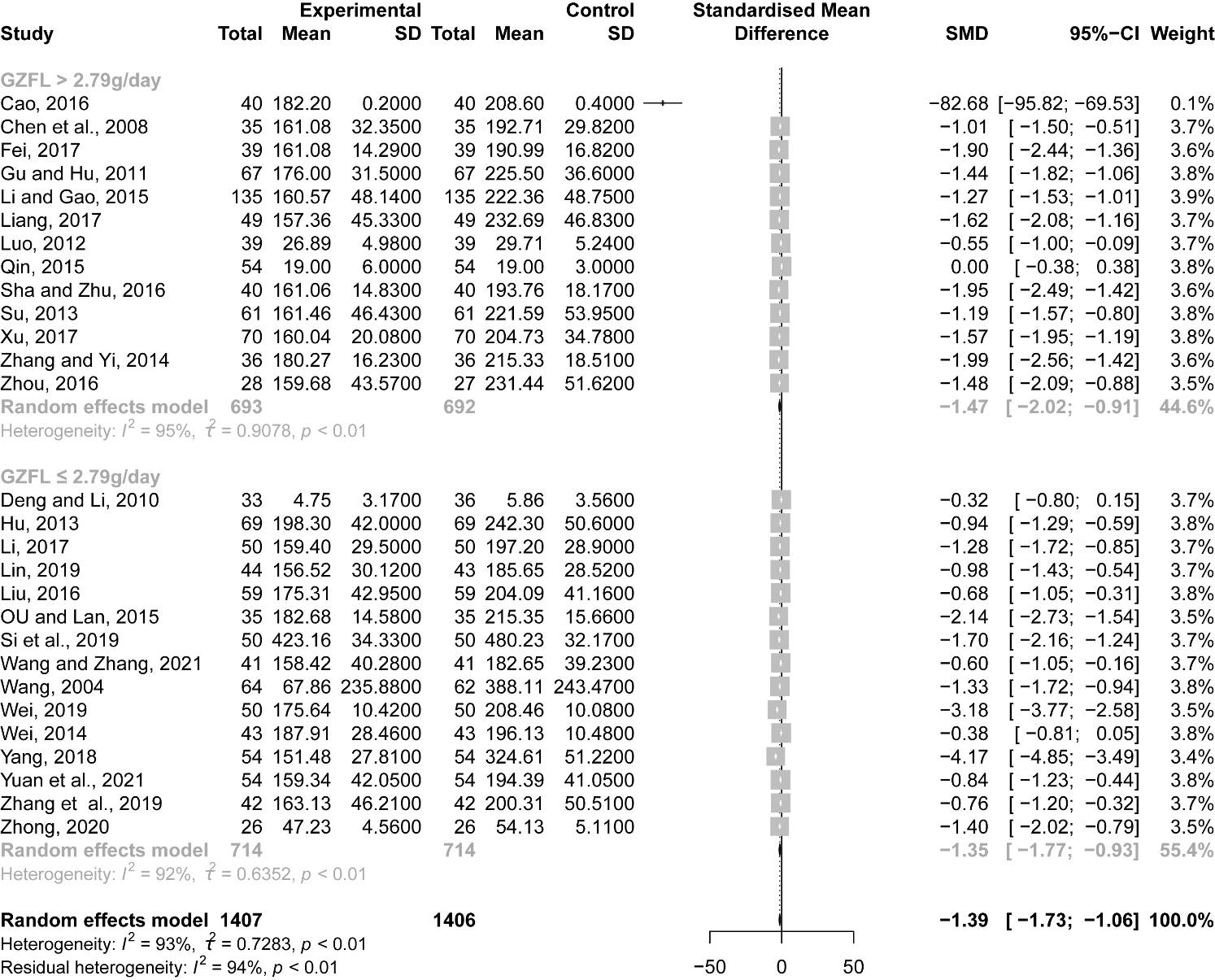
**

**6.28 Subgroup analysis of the E_2_ according to the MFP does.**

**
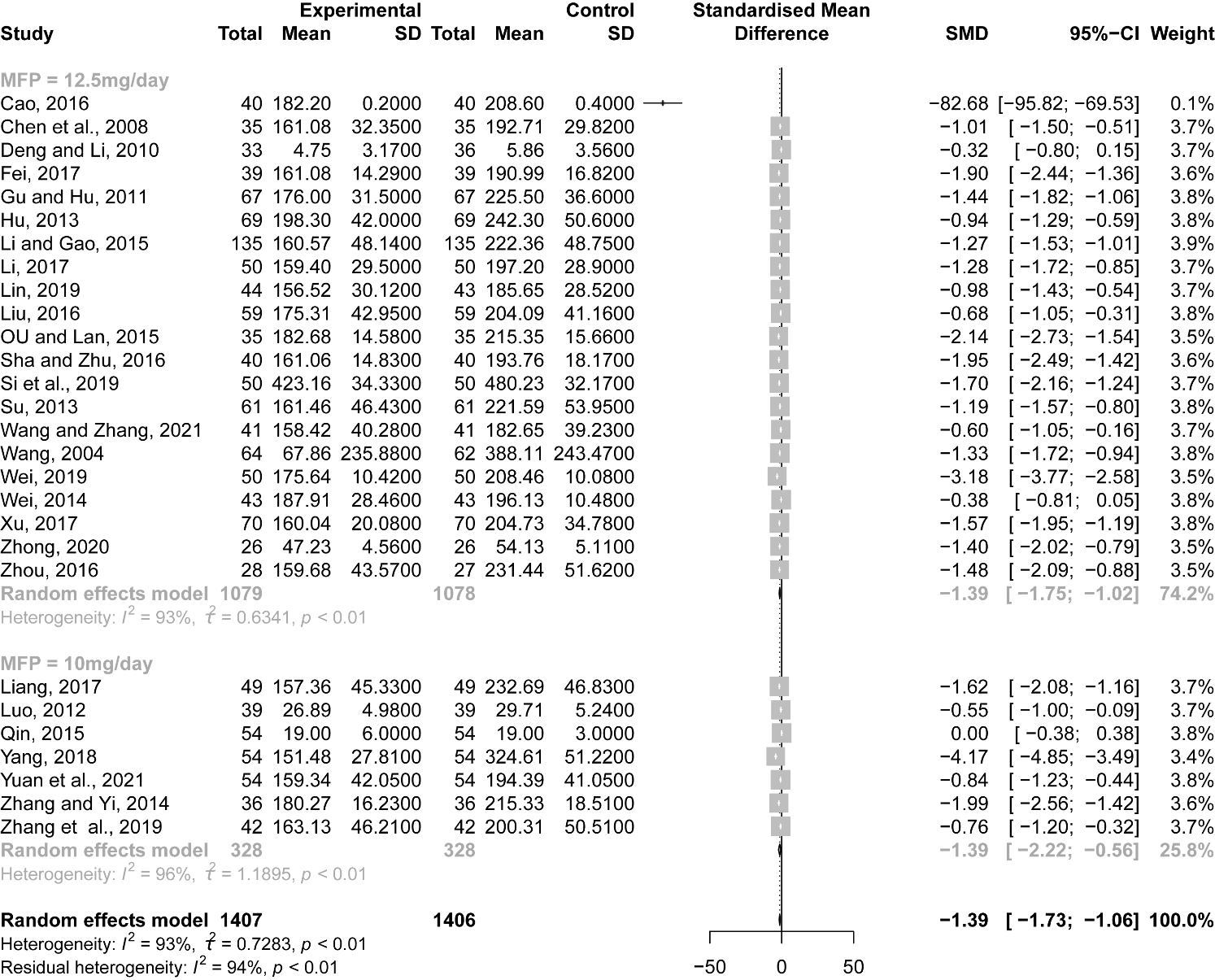
**

**6.29 Subgroup analysis of the E_2_ according to the treatment duration.**

**
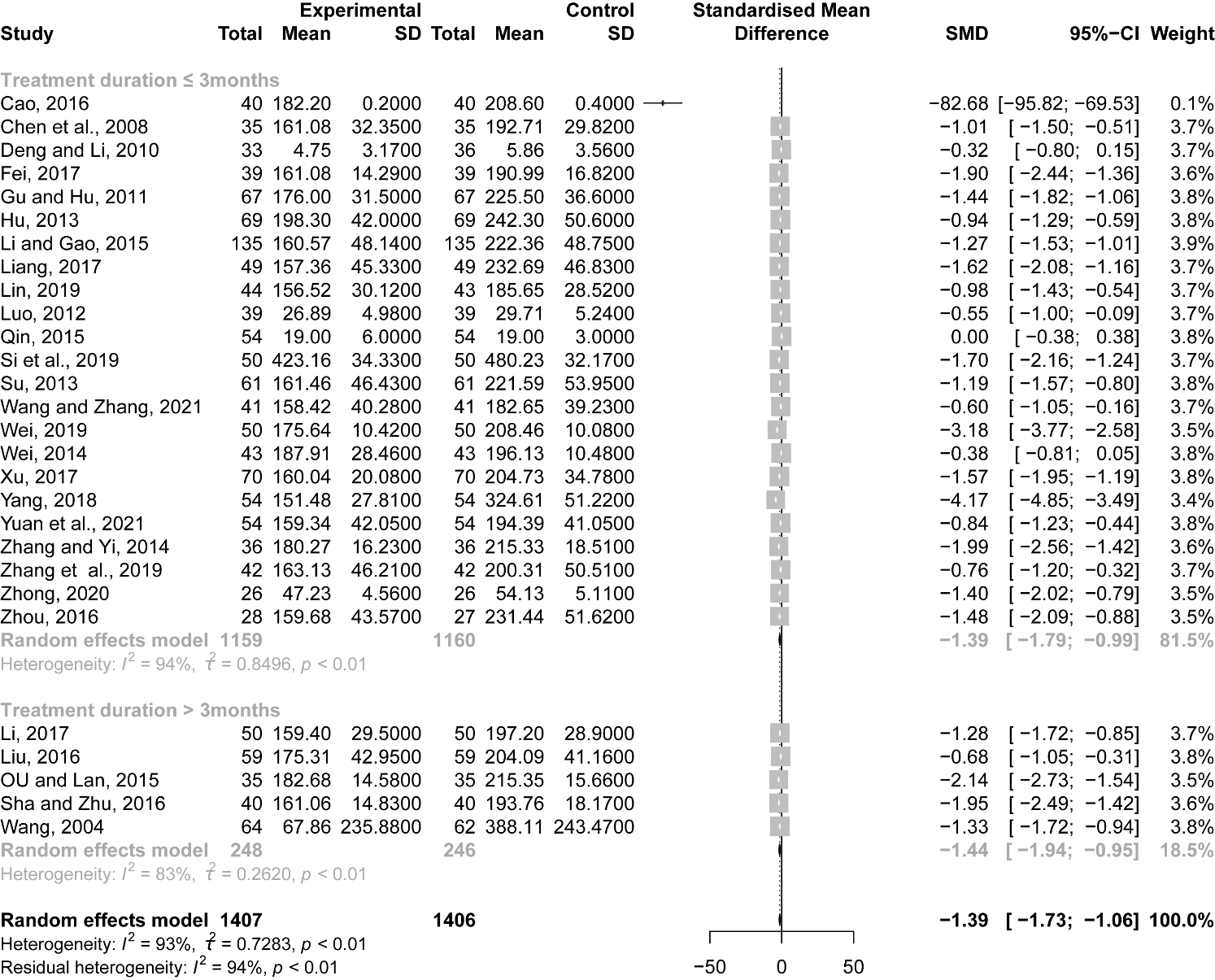
**

**6.30 Subgroup analysis of the E_2_ according to the age.**

**
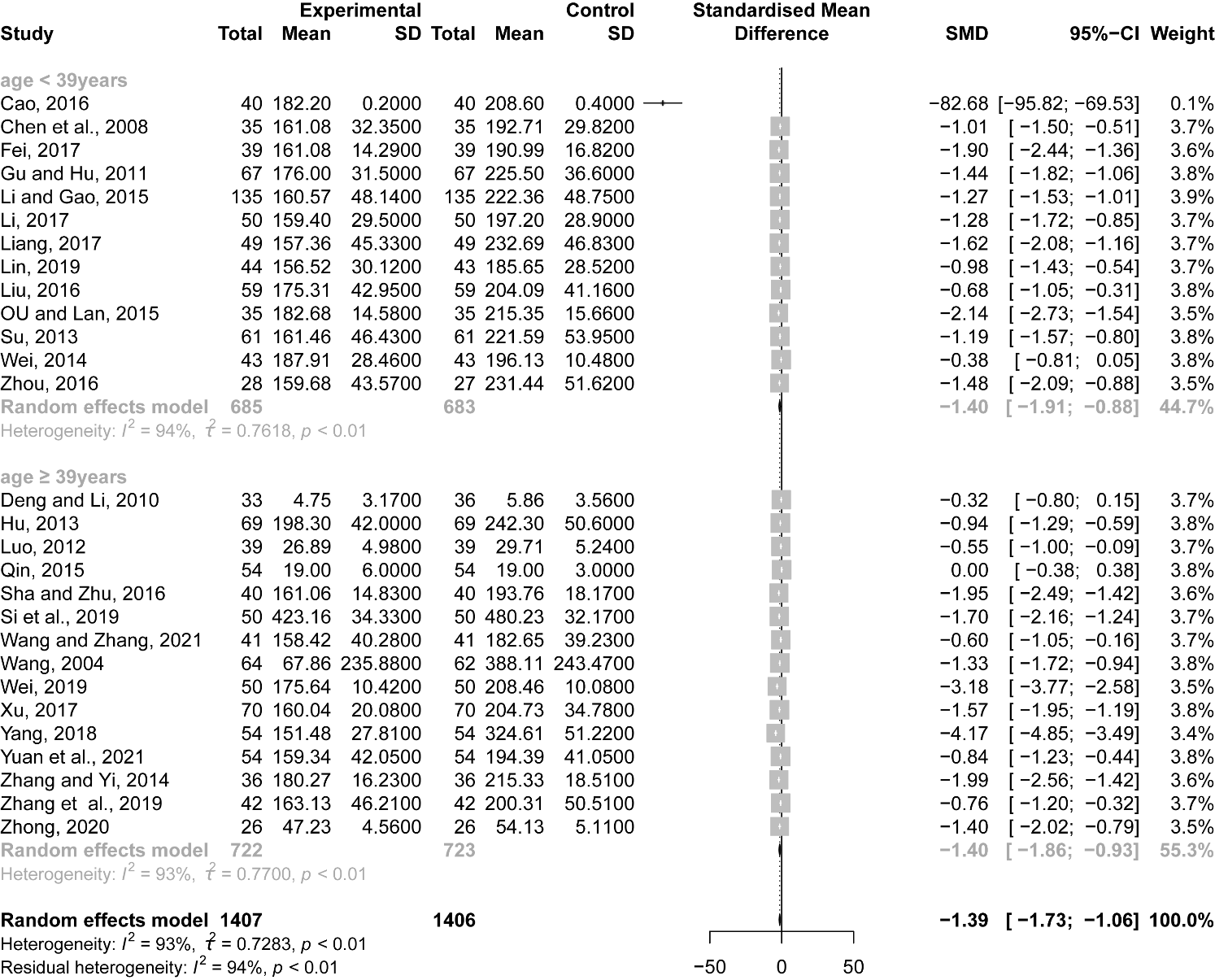
**

**6.31Sensitivity analyses of the P.**

**
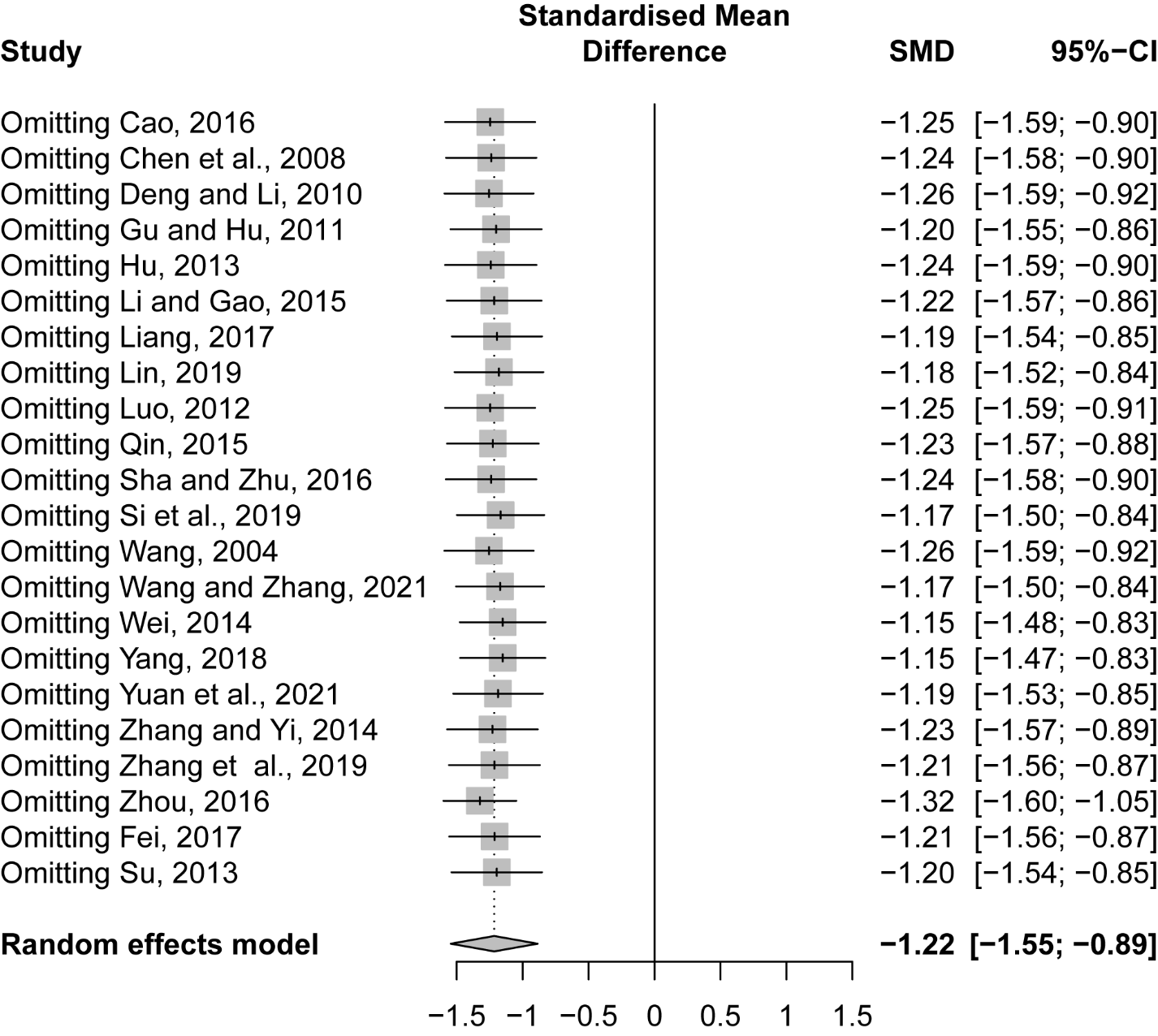
**

**6.32 Subgroup analysis of the P according to the GZFL does.**

**
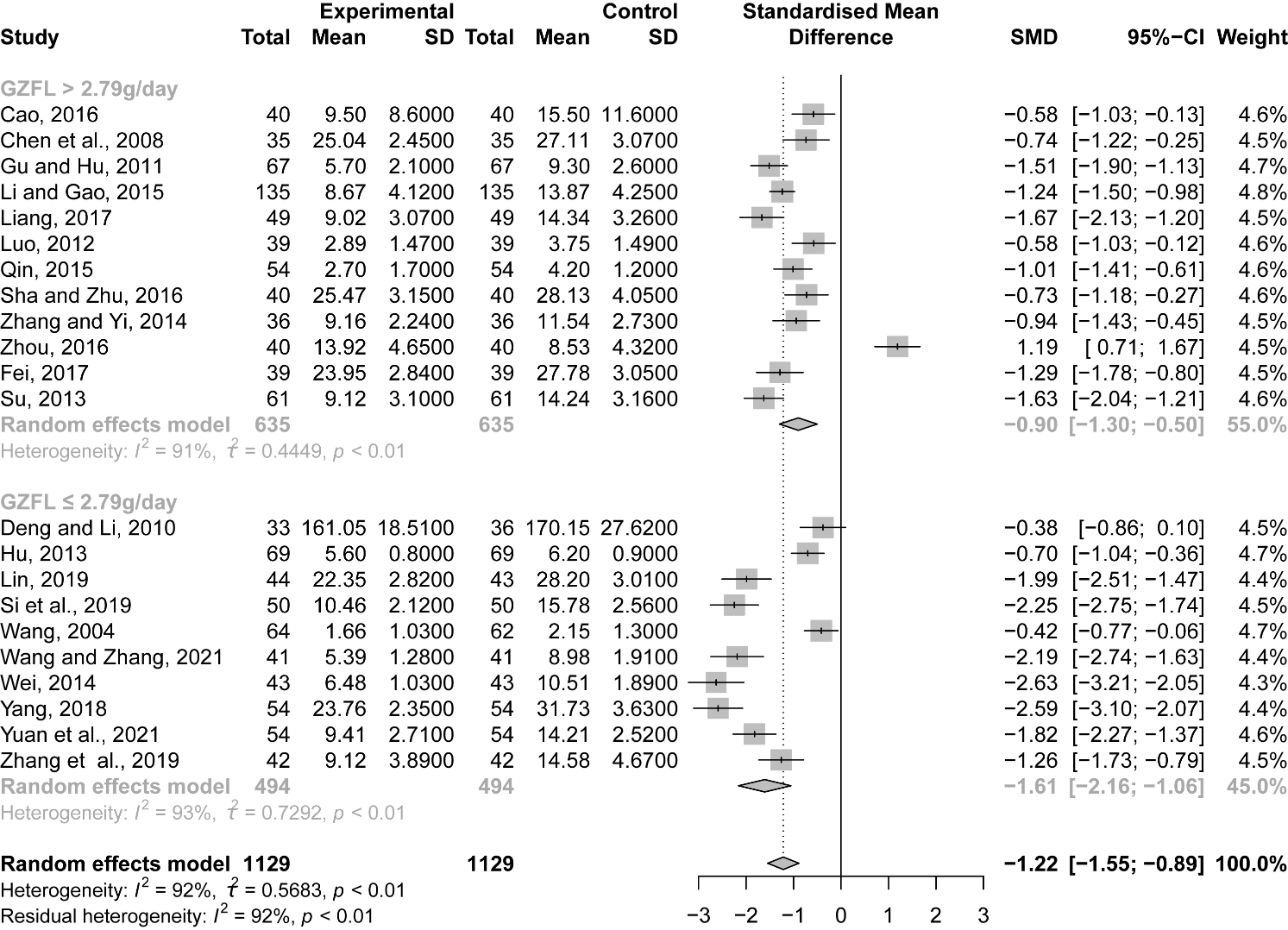
**

**6.33 Subgroup analysis of the P according to the MFP does.**

**
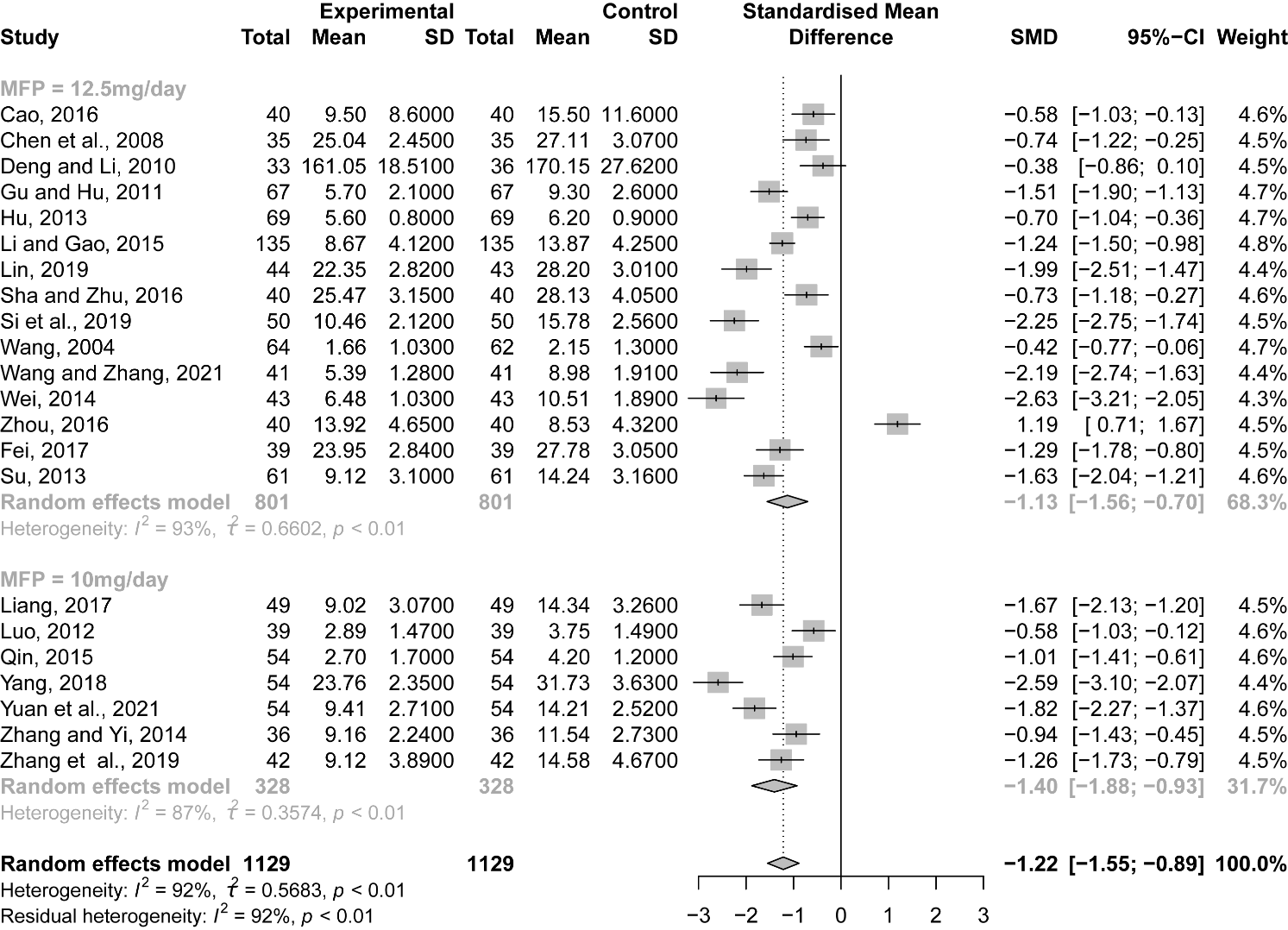
**

**6.34 Subgroup analysis of the P according to the treatment duration.**

**
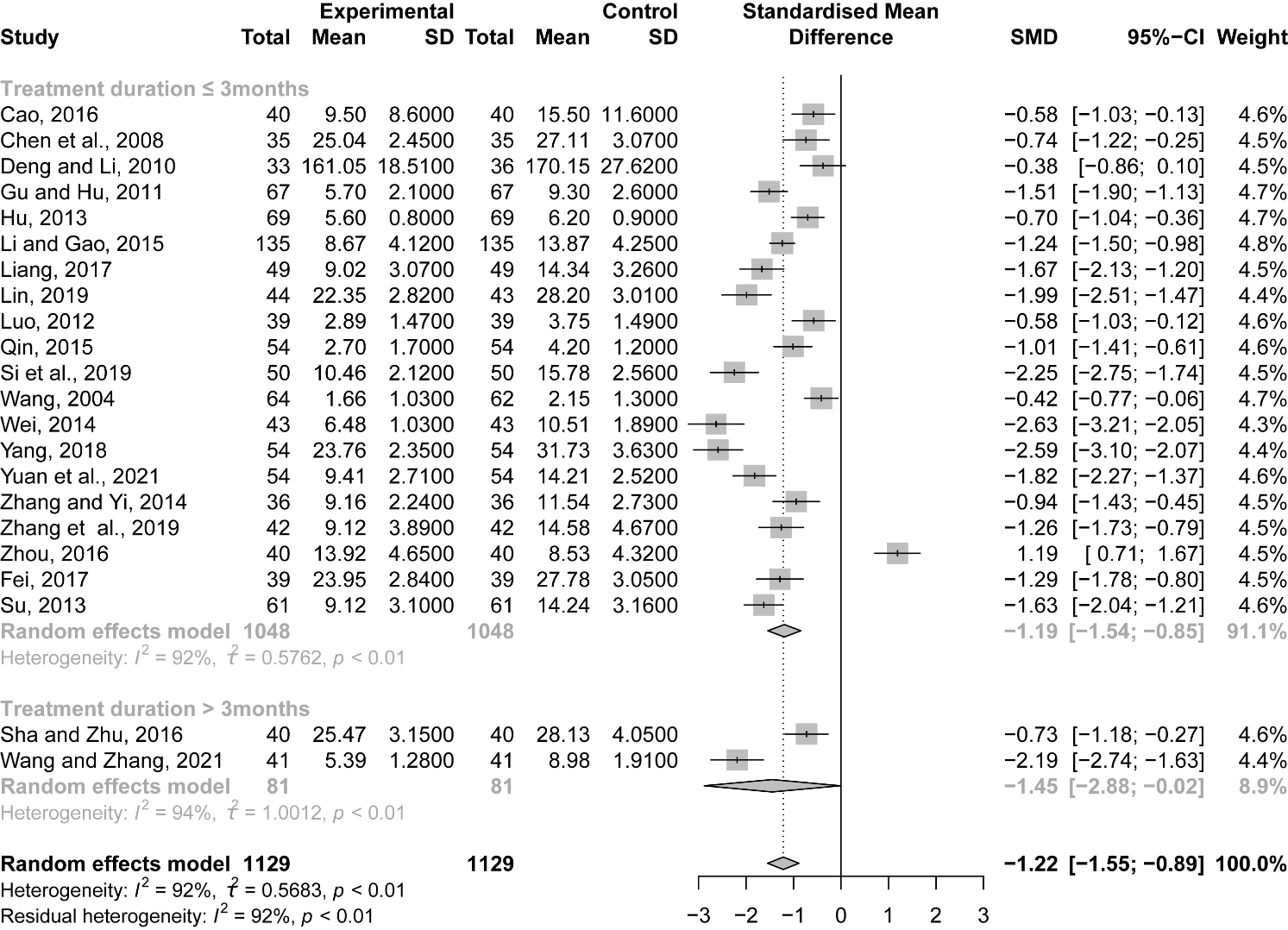
**

**6.35 Subgroup analysis of the P according to the age.**

**
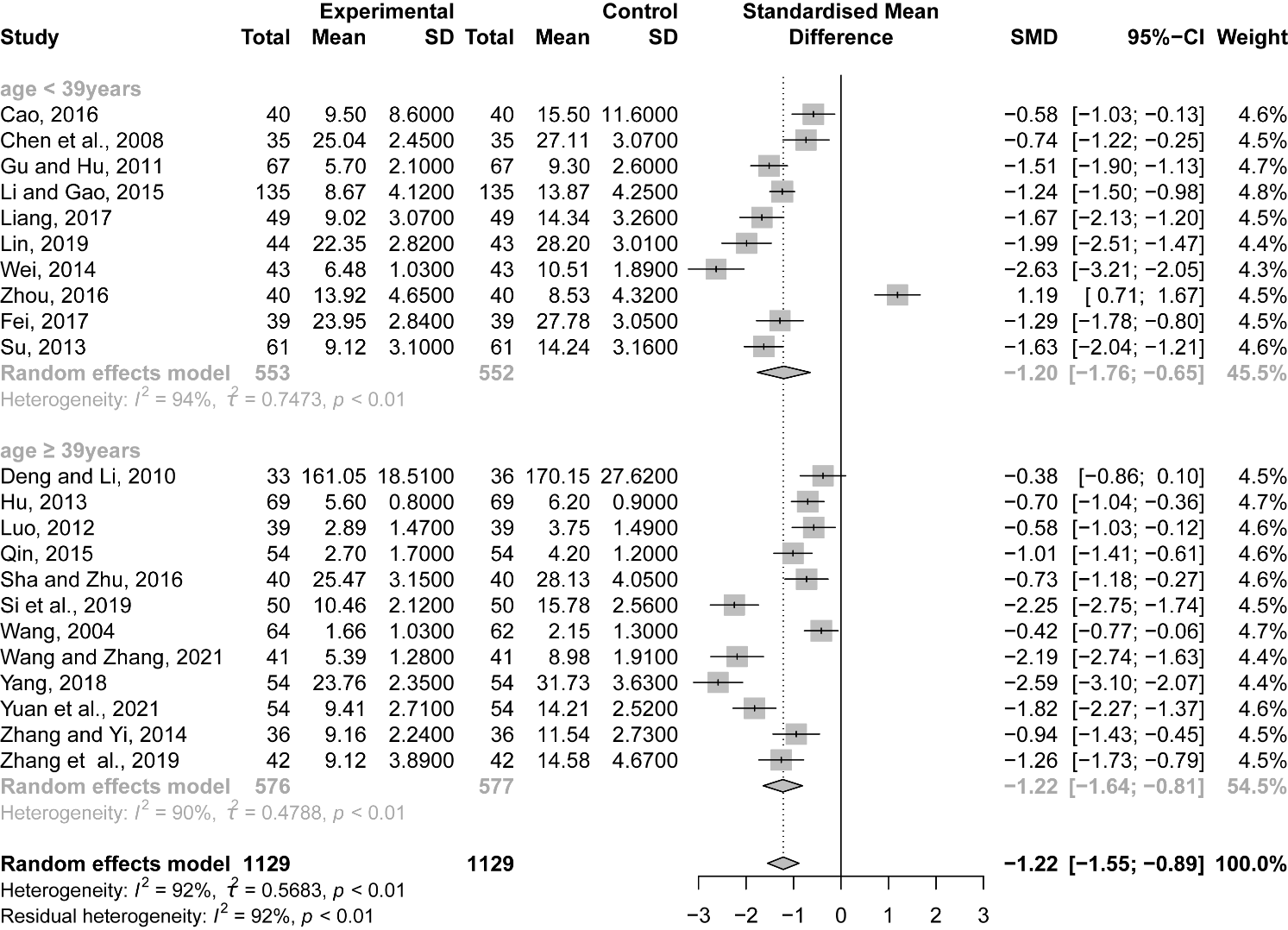
**

**6.36 Sensitivity analyses of the LH.**

**
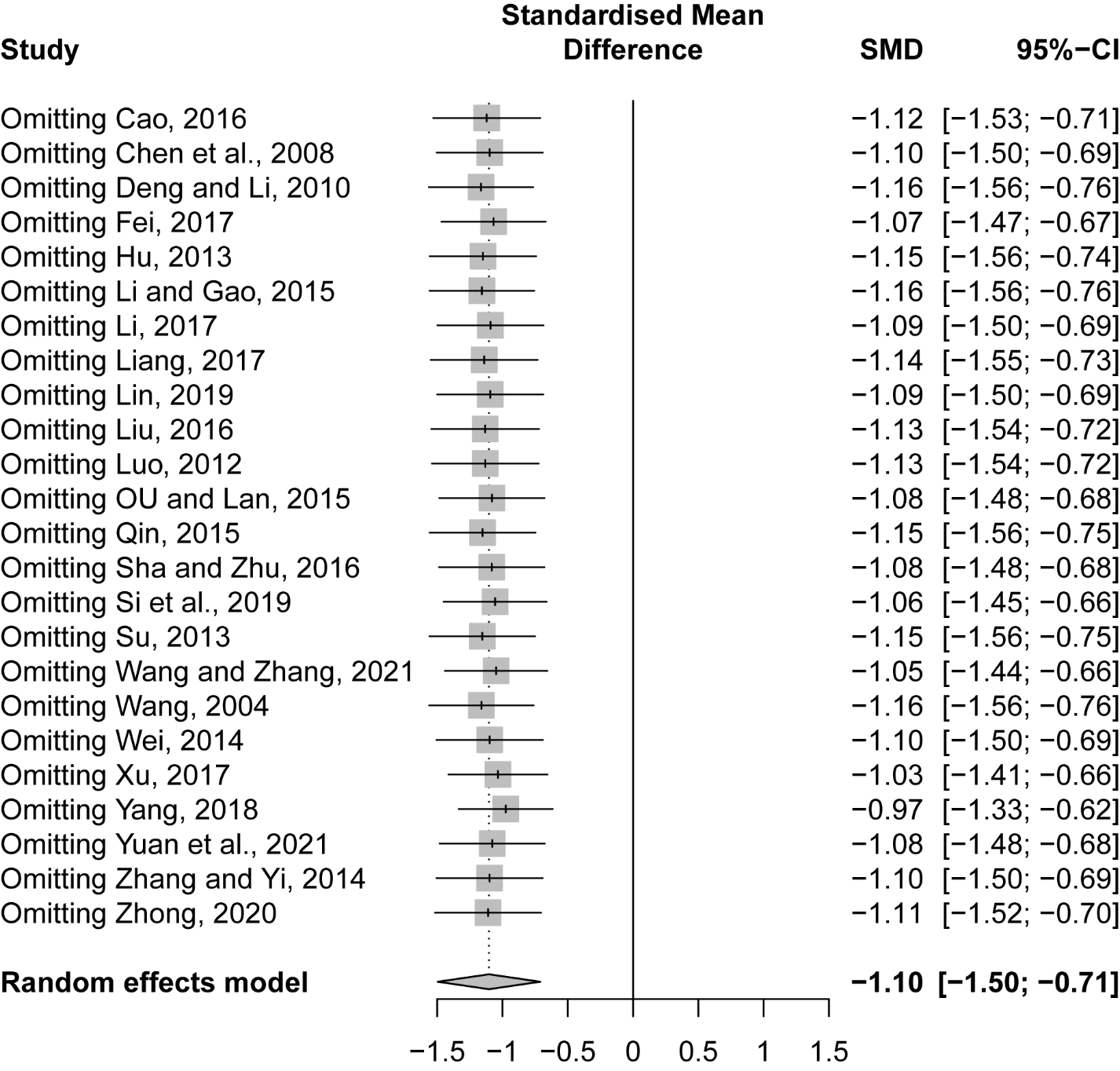
**

**6.37 Subgroup analysis of the LH according to the GZFL does.**

**
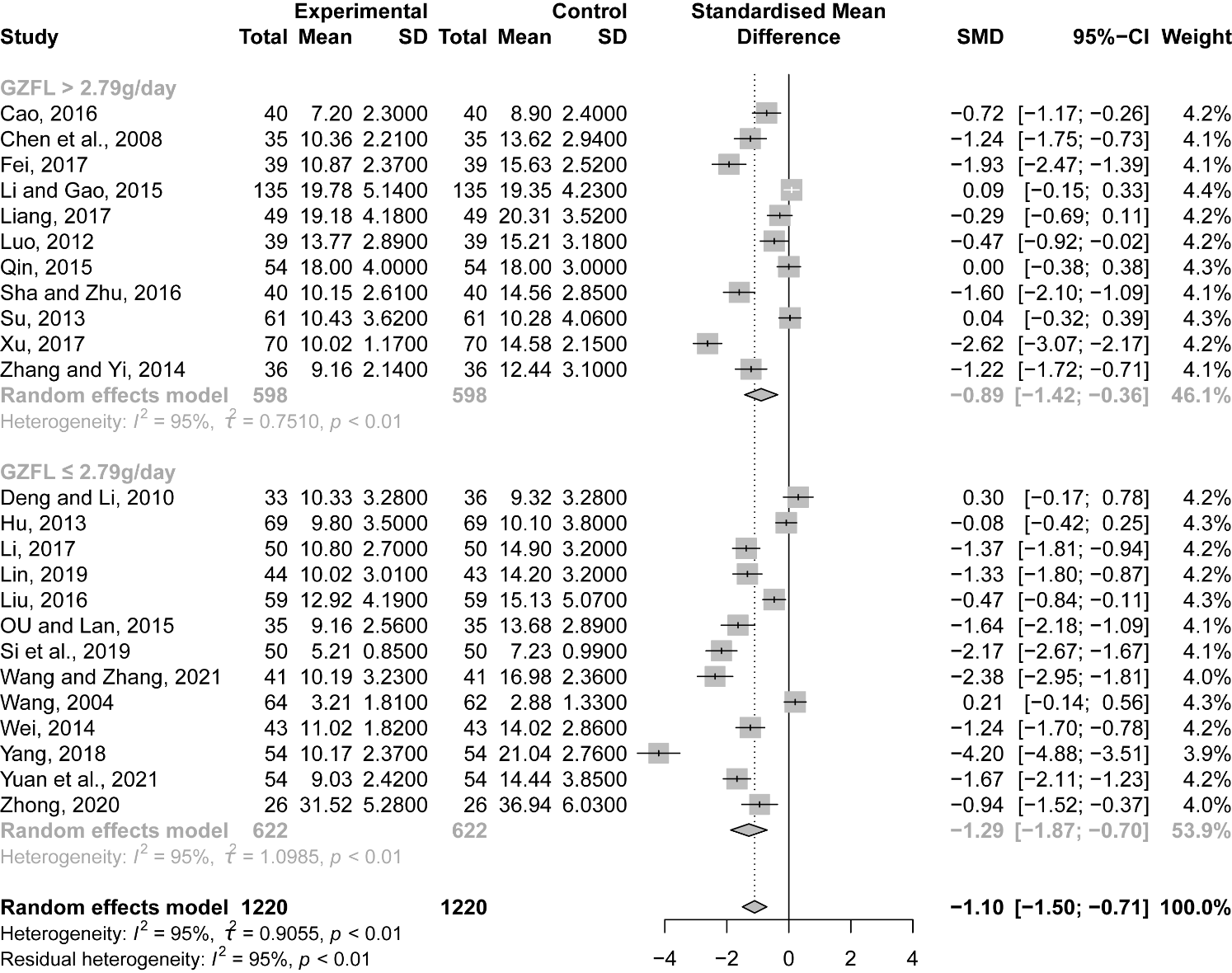
**

**6.38 Subgroup analysis of the LH according to the MFP does.**

**
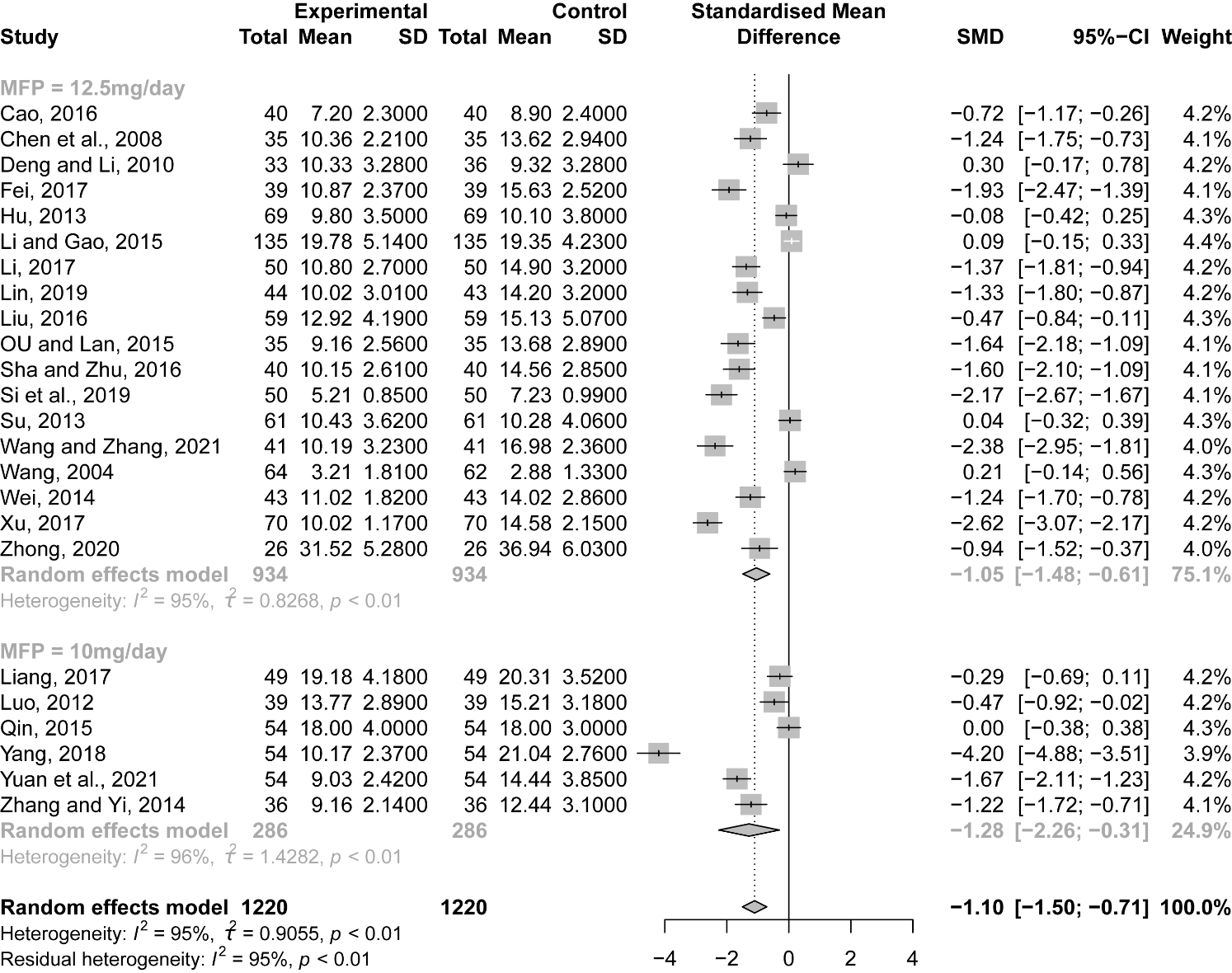
**

**6.39Subgroup analysis of the LH according to the treatment duration.**

**
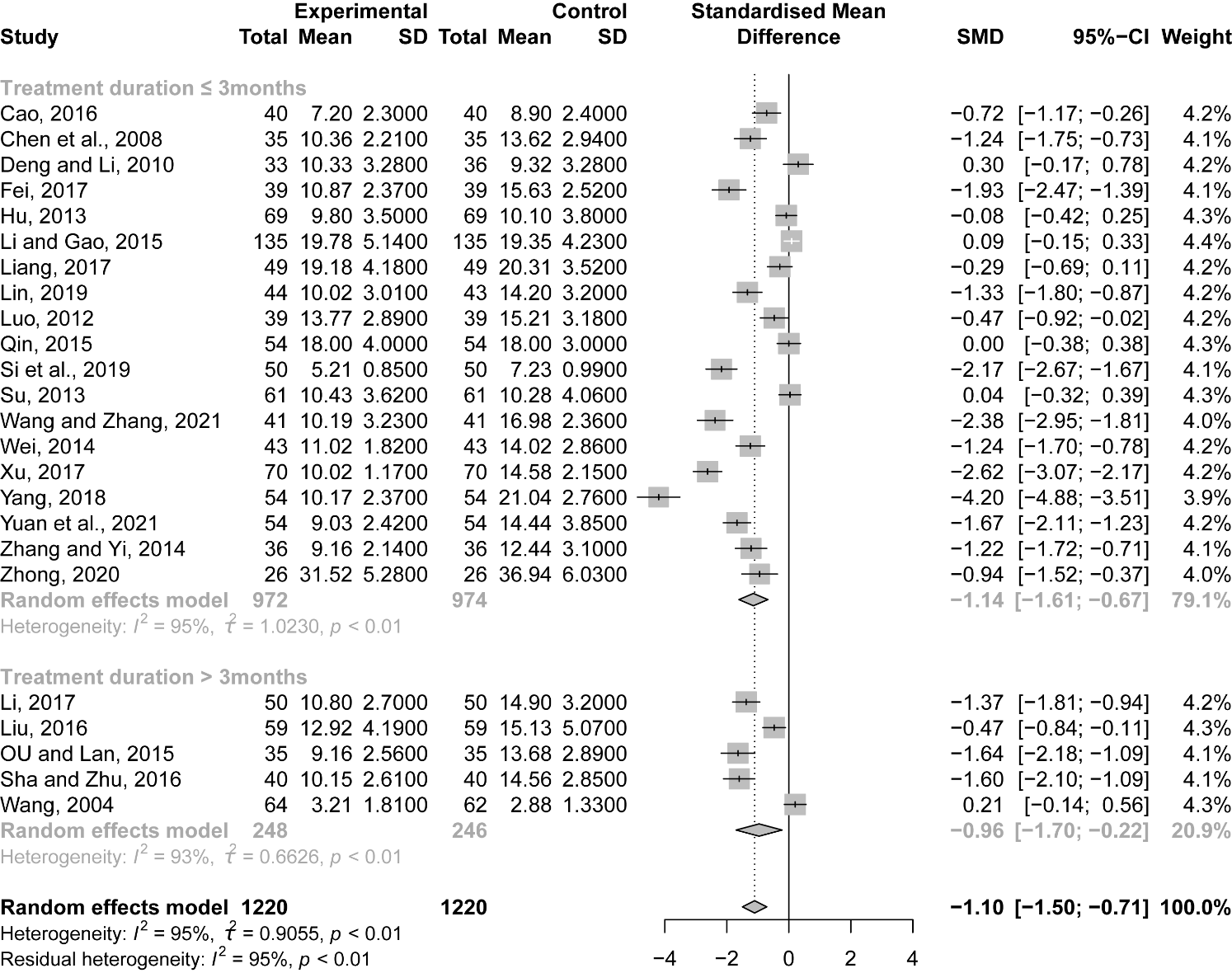
**

**6.40 Subgroup analysis of the LH according to the age.**

**
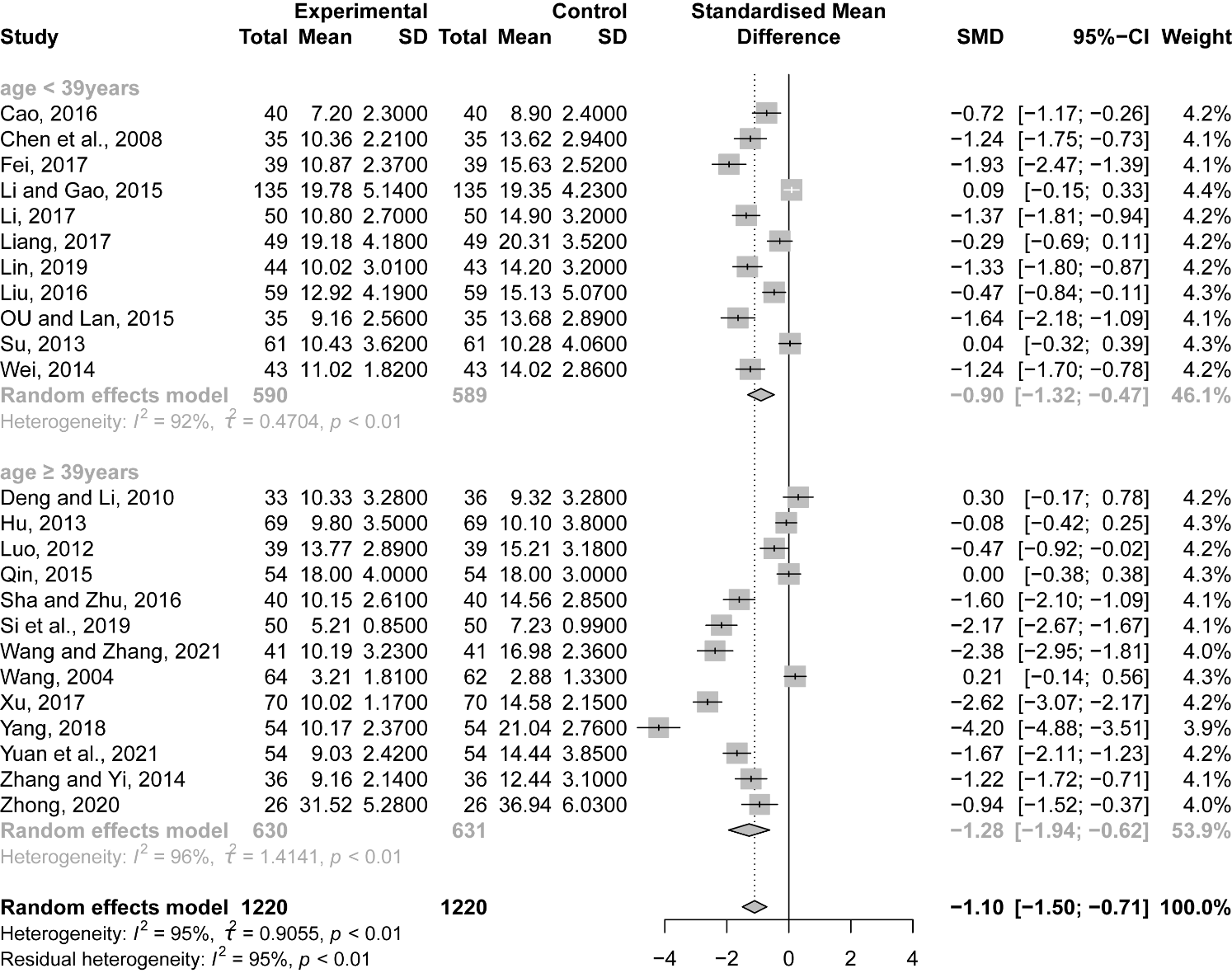
**

**6.41Sensitivity analyses of the MF.**

**
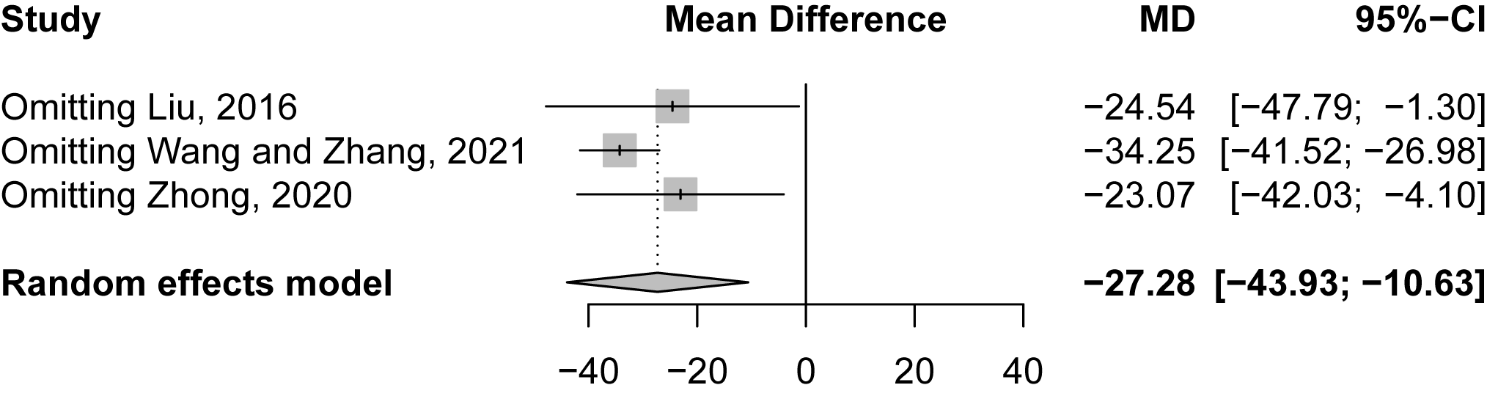
**

**6.42 Funnel plot of CER

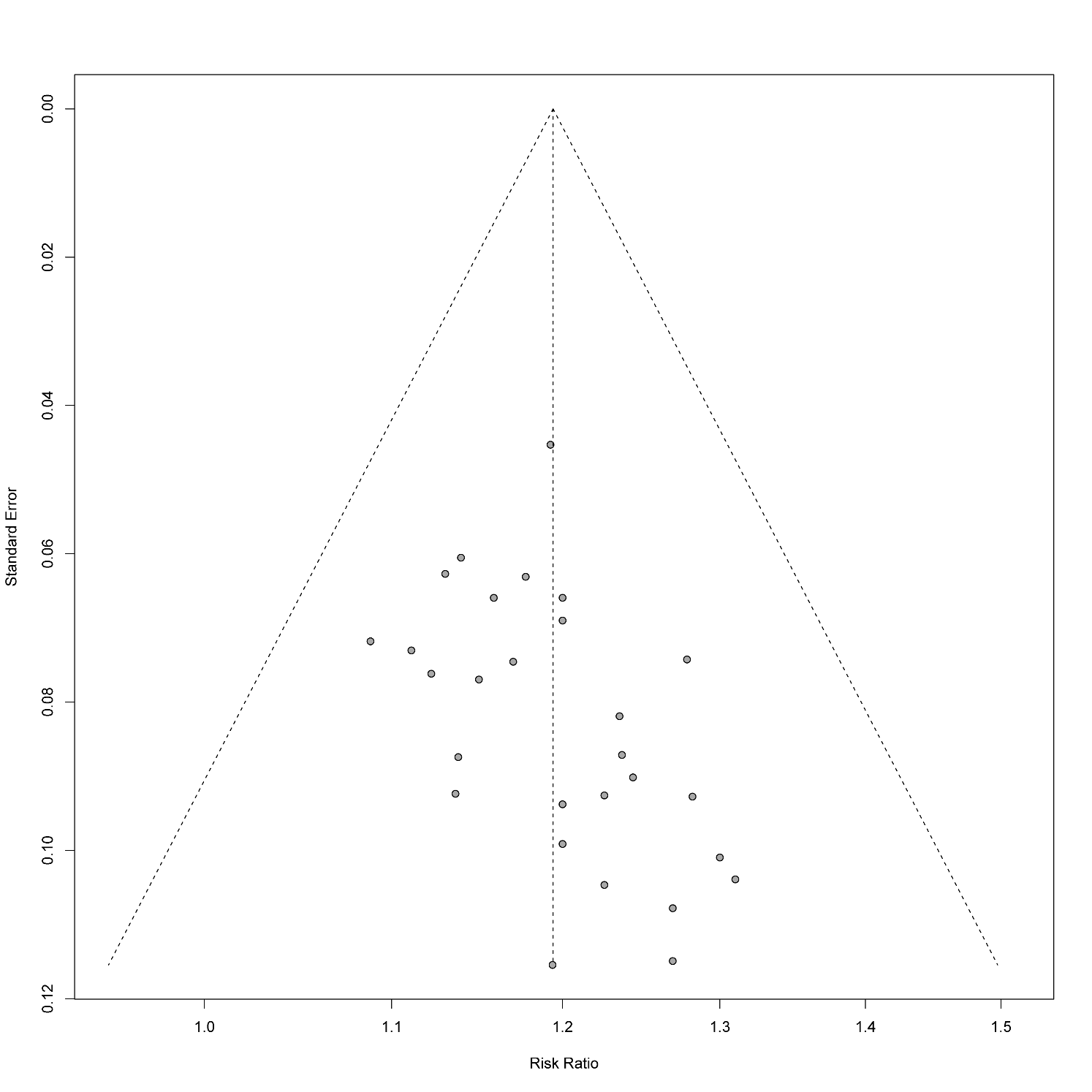
**

**6.43 Funnel plot of UFV

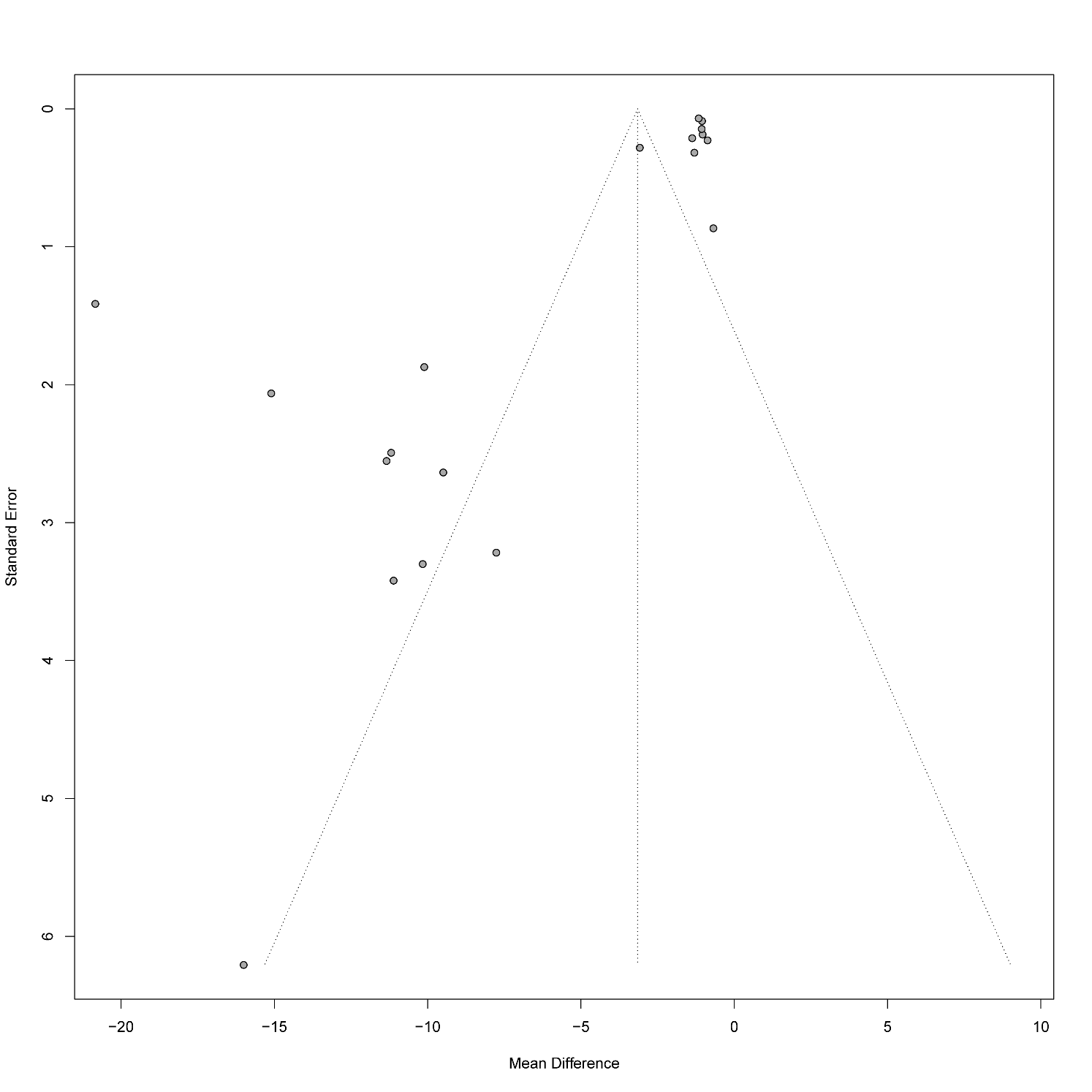
**

**6.44 Trim and fill of UFV Funnel plot**

**
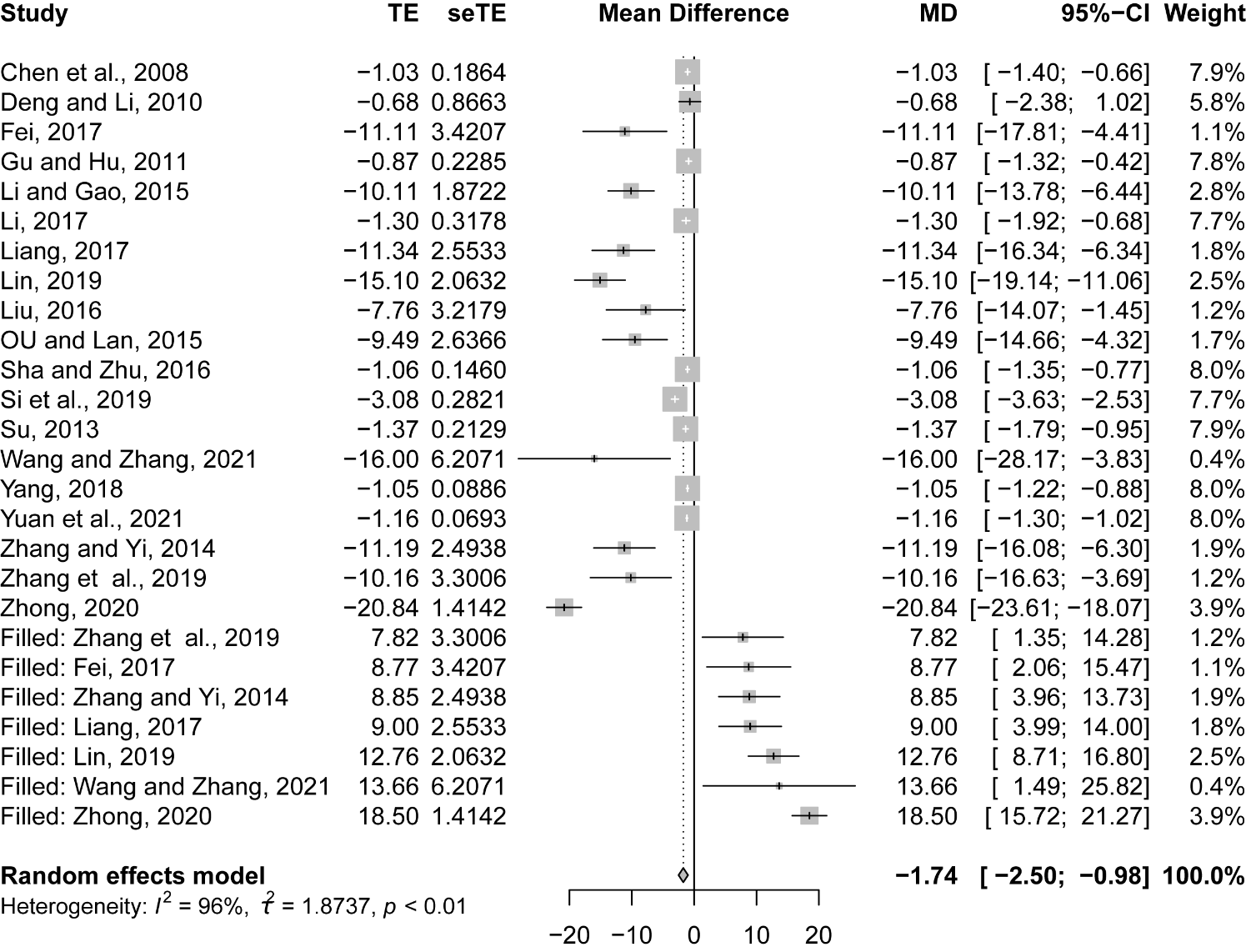
**

**6.45 Funnel plot of ADR

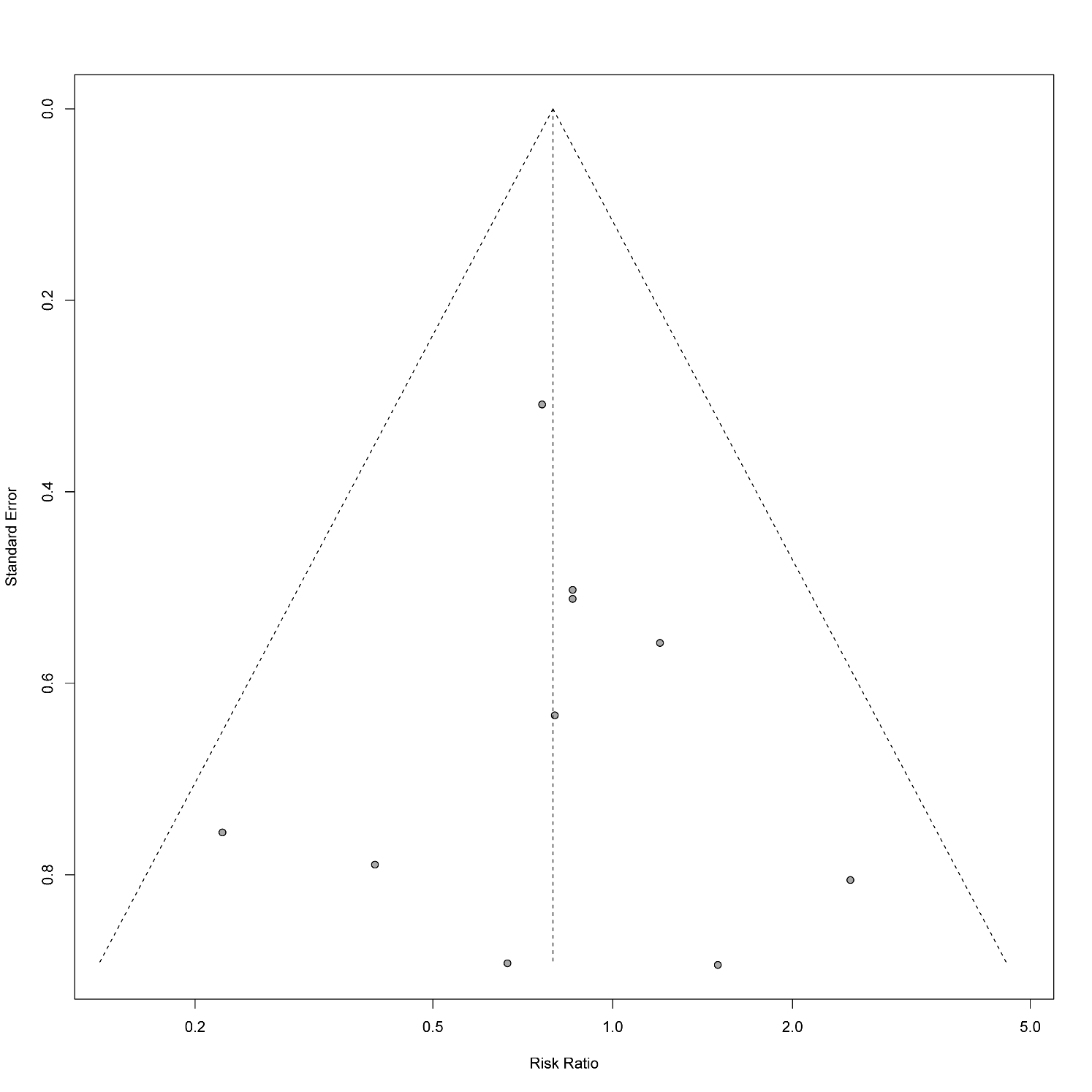
**

**6.46 Funnel plot of FSH

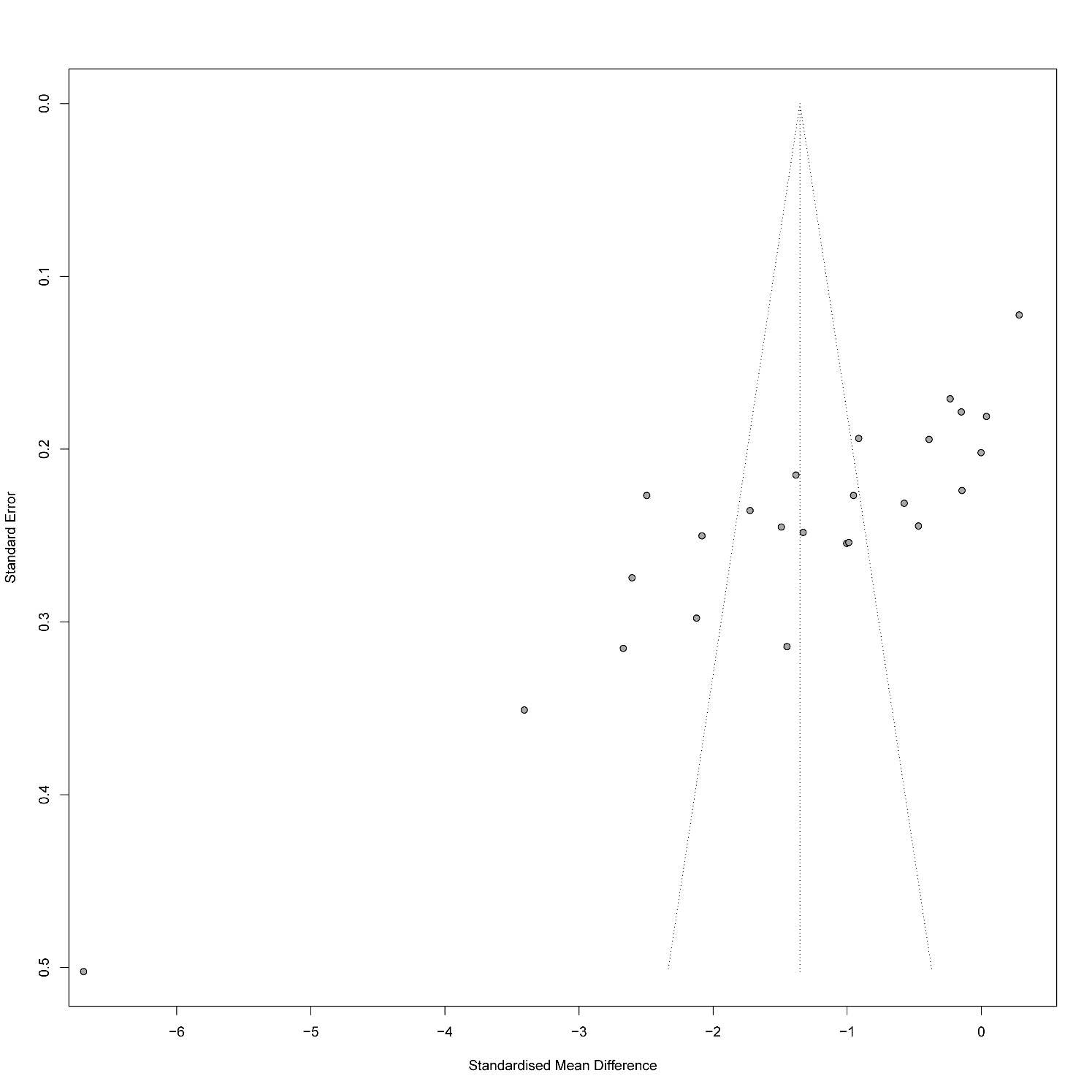
**

**6.47 Trim and fill of FSH Funnel plot**

**
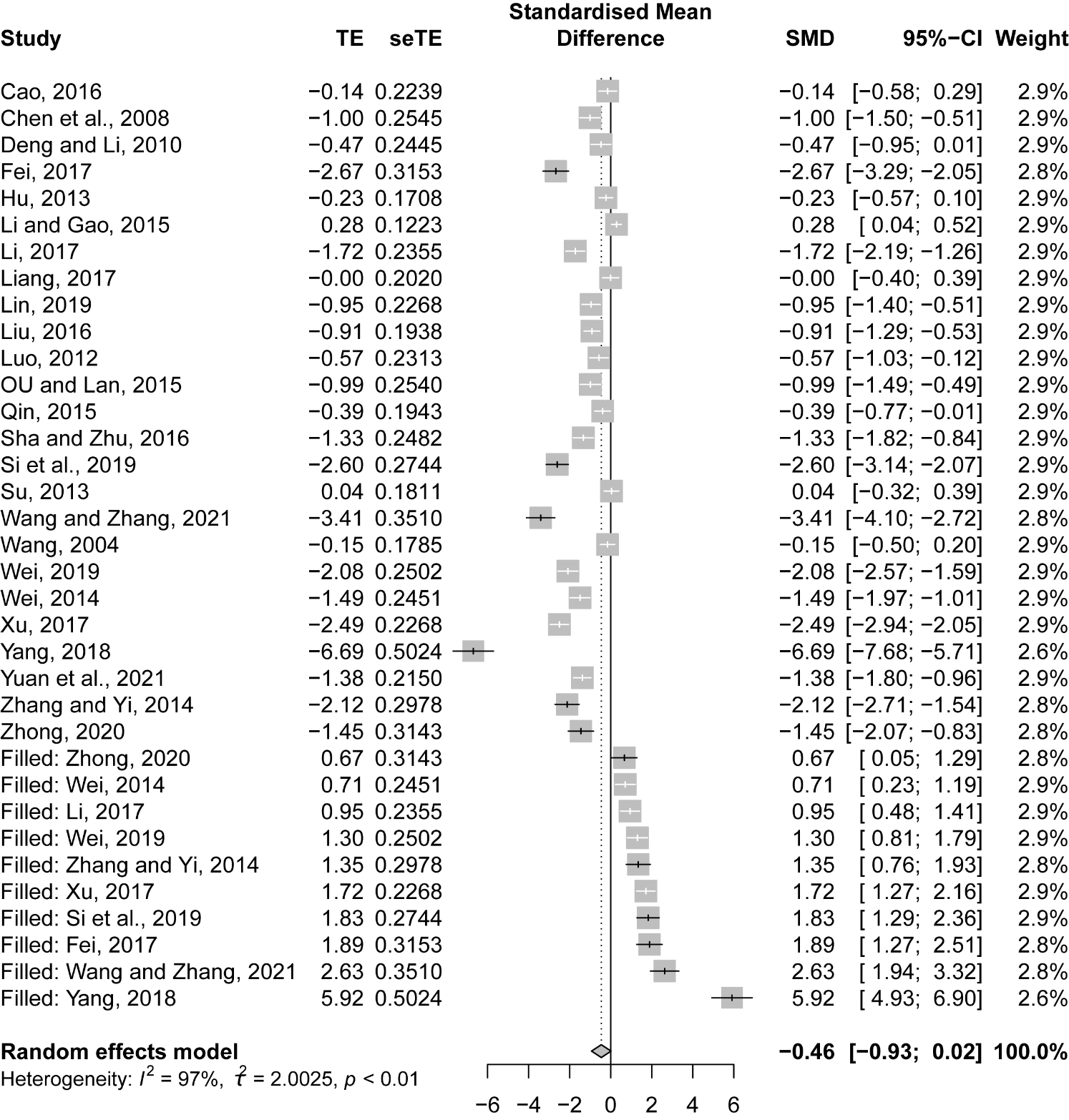
**

**6.48 Funnel plot of E_2_

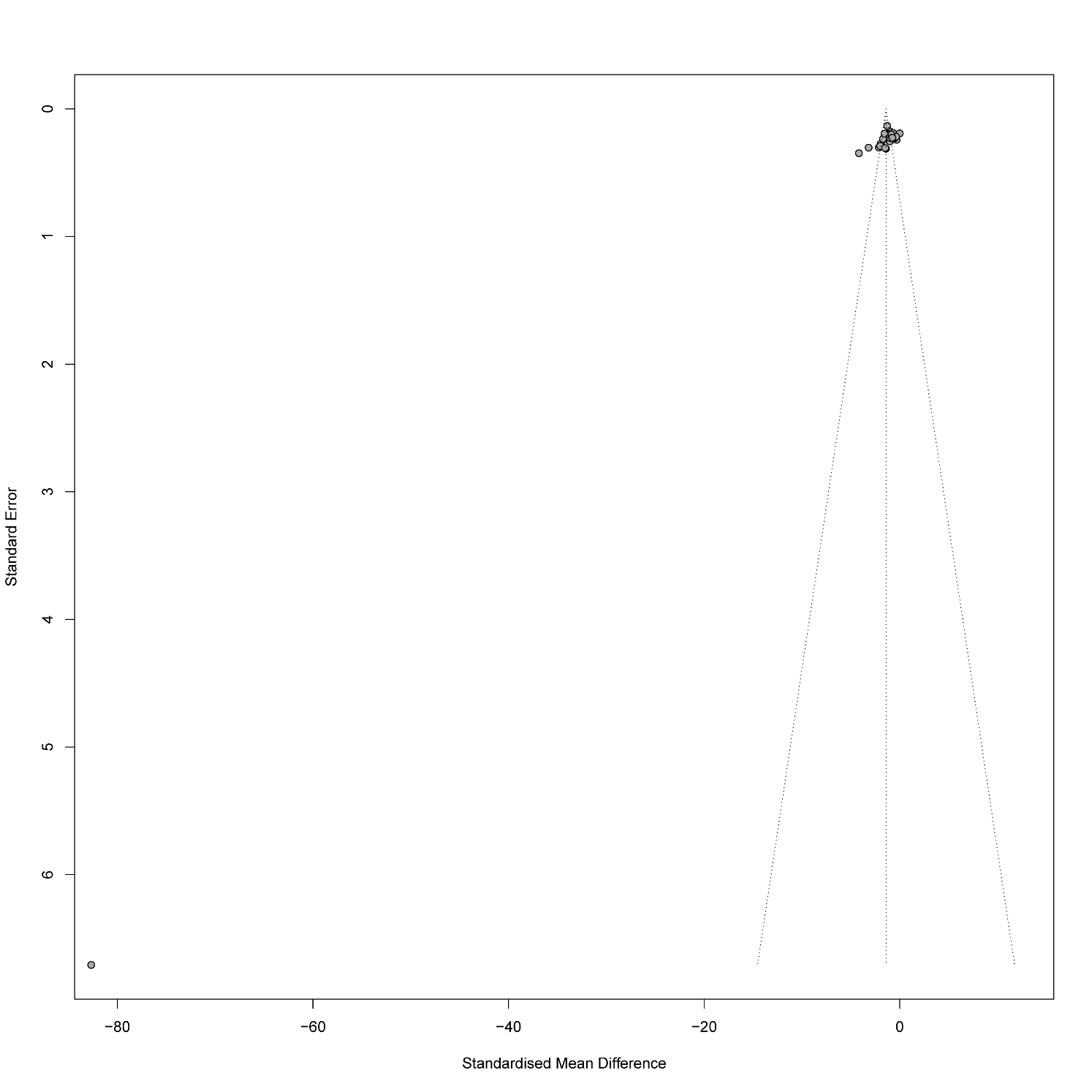
**

**6.49 Trim and fill of E_2_ Funnel plot**

**
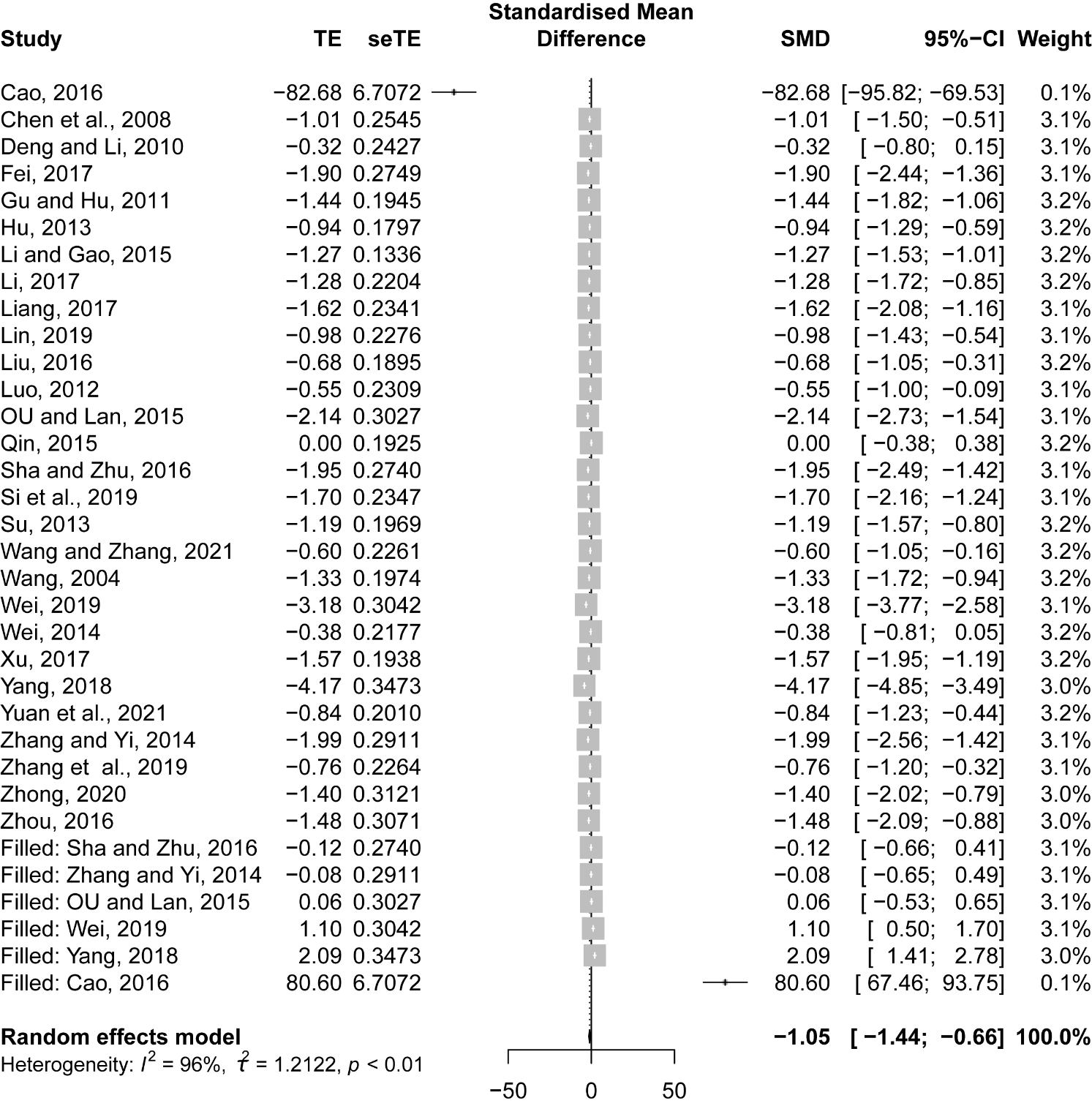
**

**6.50 Funnel plot of P

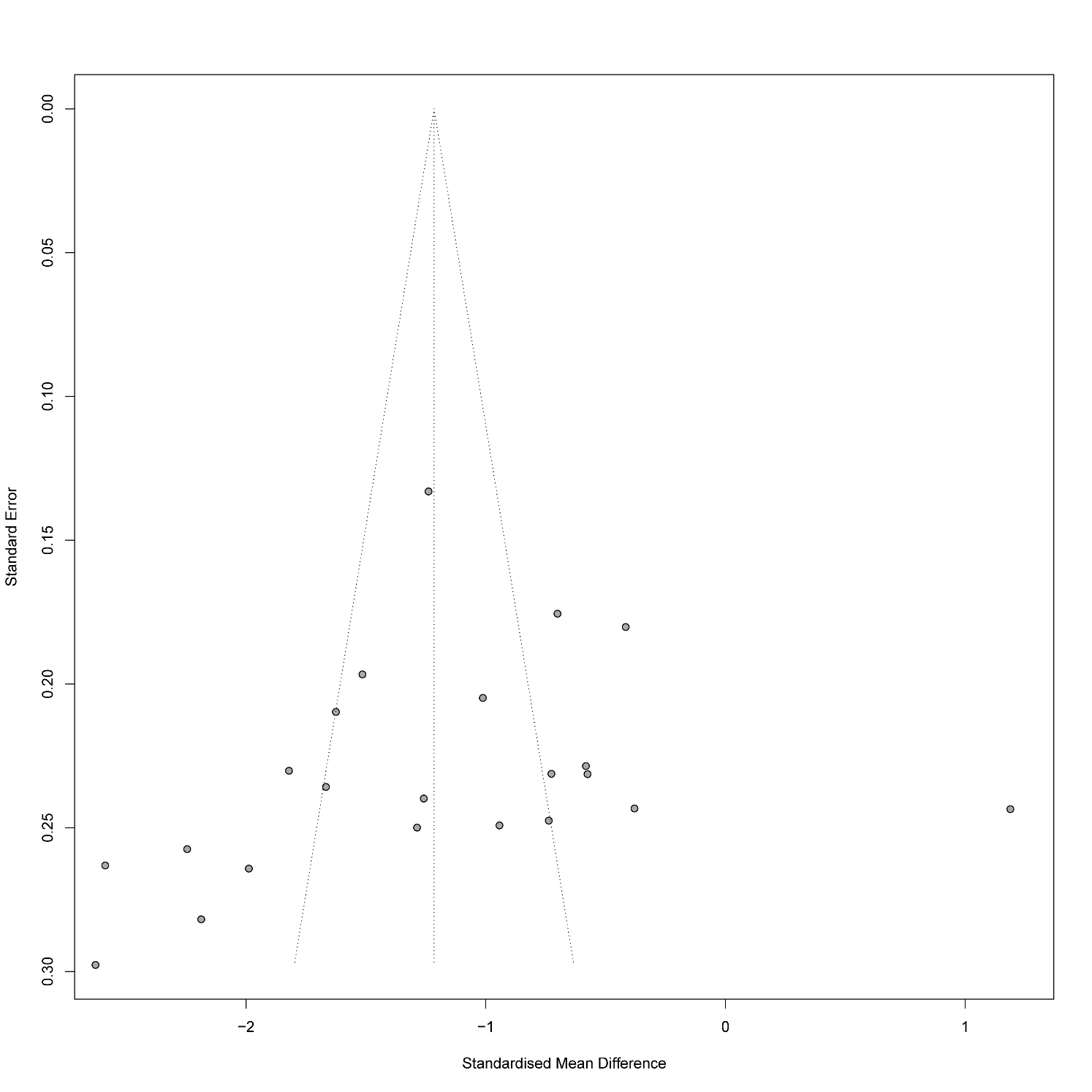
**

**6.51 Funnel plot of LH**

**
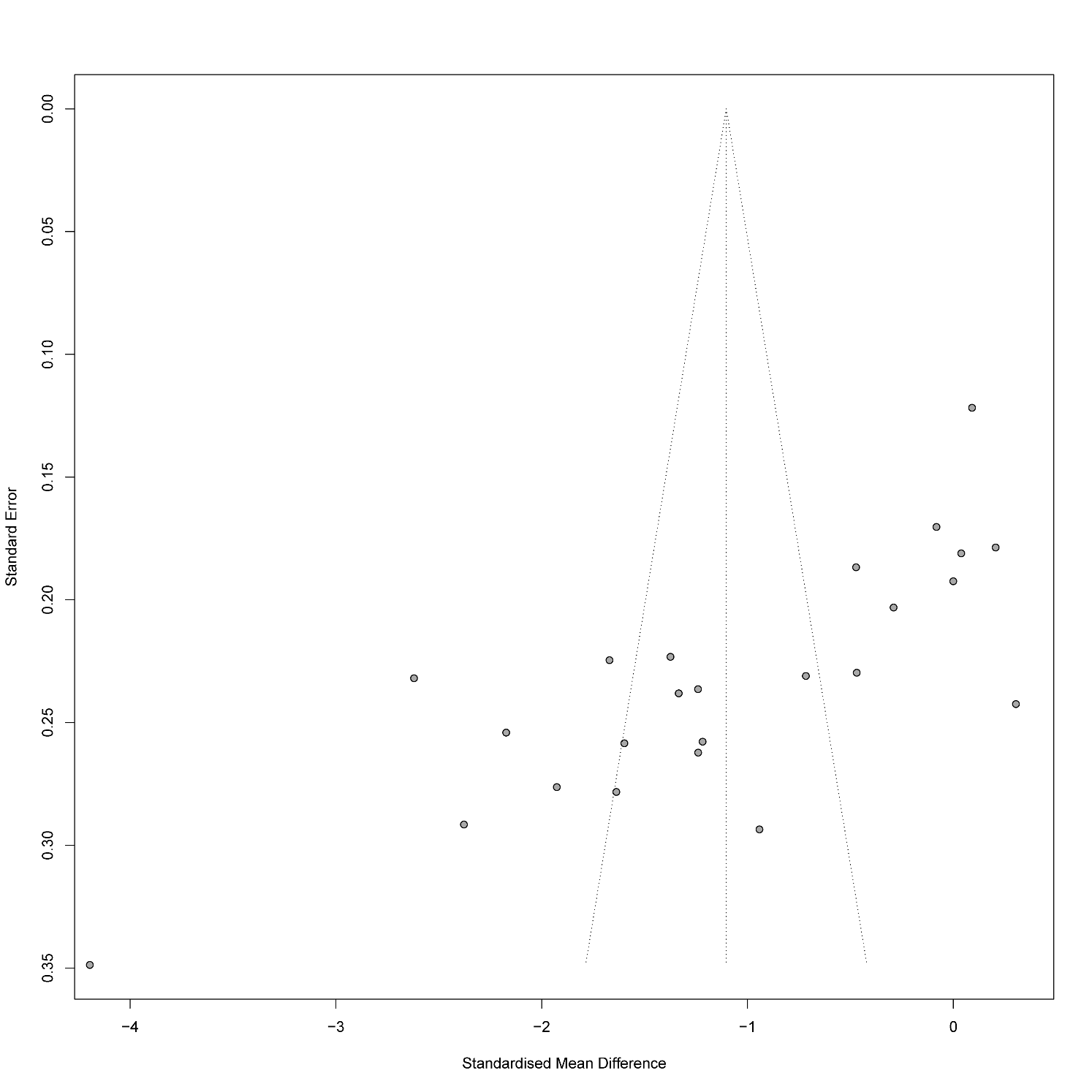
**

**6.52 Trim and fill of LH Funnel plot**

**
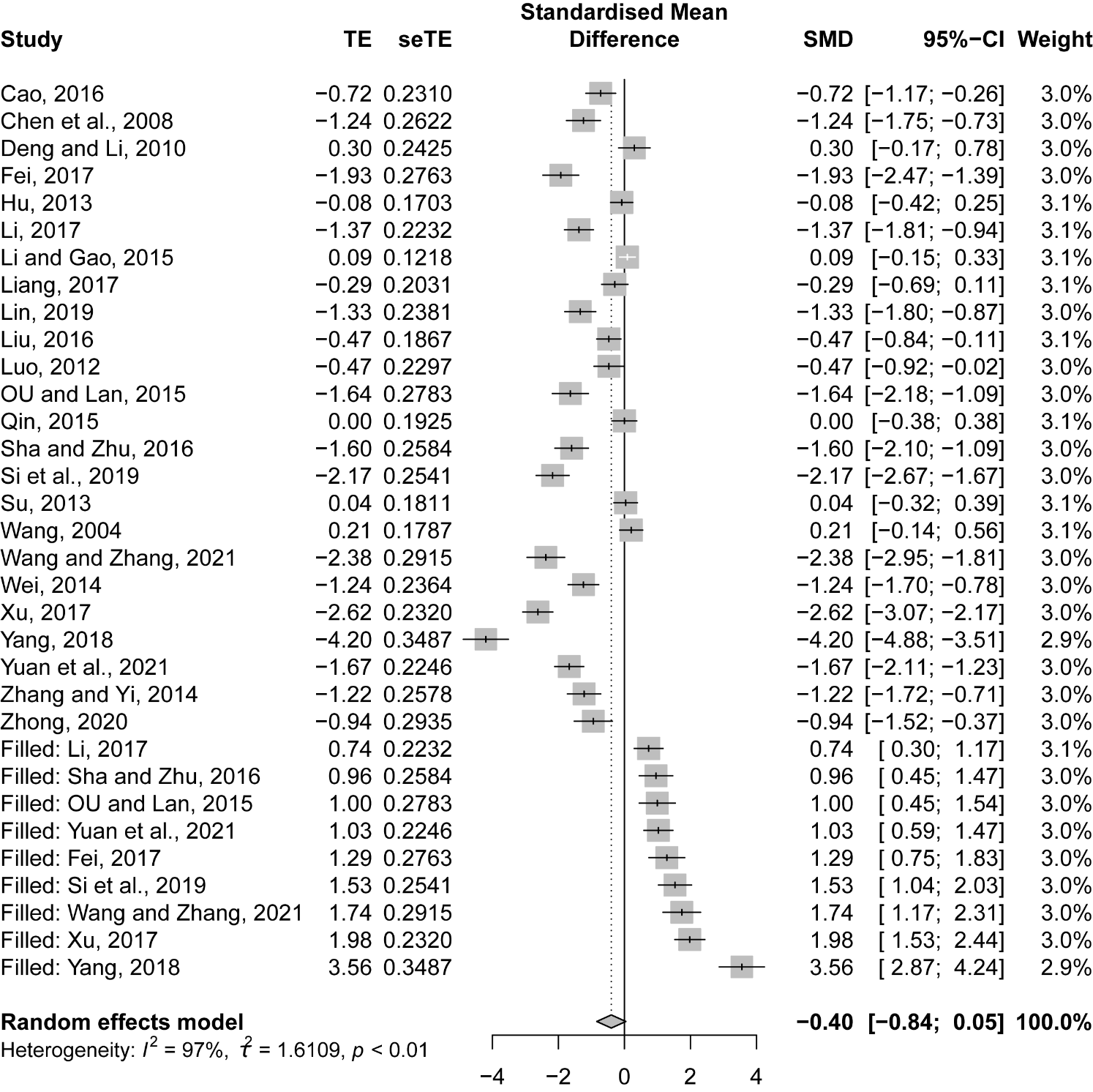
**
